# Supplementary material for: Multiscale spatial mapping of cell populations across anatomical sites in healthy human skin and basal cell carcinoma
Source: Proc Natl Acad Sci U S A. 2024 Jan 2;121(2):e2313326120. doi: 10.1073/pnas.2313326120 (PMC10786309; doi:10.1073/pnas.2313326120)
Supplement: Supplementary file 1 — Appendix 01 (PDF) [file pnas.2313326120.sapp.pdf]

Title Page

Multi-scale spatial mapping of cell populations across anatomical sites in healthy human skin and basal cell carcinoma

Authors: Clarisse Ganier[a,1], Pavel Mazin[b], Gabriel Herrera-Oropeza[c], Xinyi Du-Harpur[a,d], Matthew Blakeley[a], Jeyrroy Gabriel[a], Alexander V. Predeus[b], Batuhan Cakir[b], Martin Prete[b], Nasrat Harun[a], Jean-Francois Darrigrand[a], Alexander Haiser[a], Saranya Wyles[e], Tanya Shaw[f], Sarah A. Teichmann[b,g], Muzlifah Haniffa[b,h,i], Fiona M. Watt[a,j,1,2], Magnus D. Lynch[a,k,1,2]

Affiliations:

[a]Centre for Gene Therapy and Regenerative Medicine, King's College London, Guy's Hospital, London SE1 9RT, United Kingdom

[b]Wellcome Sanger Institute, Wellcome Genome Campus, Hinxton, Cambridge CB10 1SA, United Kingdom

[c]Centre for Developmental Neurobiology, Institute of Psychiatry, Psychology and Neuroscience, King's College London, London SE1 1UL, United Kingdom

[d]The Francis Crick Institute, 1 Midland Road, London NW1 1AT, United Kingdom

[e]Department of Dermatology, Mayo Clinic, 200 First St. SW, Rochester, MN 55905, United States of America

[f]Centre for Inflammation Biology & Cancer Immunology, King's College London, London SE1 1UL, United Kingdom

[g]Theory of Condensed Matter Group, Cavendish Laboratory/ Department of Physics, University of Cambridge, JJ Thomson Ave, Cambridge CB3 0HE, United Kingdom

[h]Biosciences Institute, Newcastle University, Newcastle upon Tyne NE2 4HH, UK

[i]Department of Dermatology and NIHR Newcastle Biomedical Research Centre, Newcastle Hospitals NHS Foundation Trust, Newcastle upon Tyne NE1 4LP, United Kingdom

[j]Directors' Unit, EMBL, Meyerhofstr. 1, 69117 Heidelberg, Germany

[k]St John's Institute of Dermatology, King's College London, Guy's Hospital, London SE1 9RT, United Kingdom

[1]To whom correspondence may be addressed: C.G.; F.M.W. or M.D.L

Email: ✉ clarisse.ganier@kcl.ac.uk, ✉ fiona.watt@kcl.ac.uk, or ✉ magnus.lynch@kcl.ac.uk.

[2]F.M.W. and M.D.L. contributed equally to this work.

Key words: human cell atlas; skin; basal cell carcinoma; single cell RNA sequencing; fibroblasts

Supplementary Materials and Methods

Histology

Skin tissue samples were embedded in Tissue-Tek O.C.T. (Life Technologies, Waltham, MA, USA) and stored at –80°C prior to sectioning. Skin sections were sectioned at 10–16 µm using a Thermo Cryostar Nx70 (Thermo Fisher Scientific, Waltham, MA, USA) and placed onto SuperFrost Plus glass slides (Thermo Fisher Scientific, Waltham, MA, USA, ref J2800AMN2). Sections were stained with Harris's hematoxylin solution for 20s at room temperature and were then rinsed in tap water. Next, 0.225% Acid Alcohol (acetic acid and ethanol) in water was used to differentiate the tissue for 10s. Then, skin sections were rinsed with tap water. In the bluing step, the tissue was soaked in Scott's tap water (ref: 3802900, Leica Biosystems, Milton keynes, UK) for 10s and then rinsed with tap water. Staining was performed with 0.125% eosin Y ethanol solution for 10s. Finally, skin sections were dehydrated in IMS for 20s, then cleared in Xylene 30s and mounted with CV mounting medium (ref : 14046430011, Leica biosystem, Germany).

Epidermal thickness (µm) was quantified using a length measuring tool in Fiji software (1). At least 20 measurements were performed per H&E image. The numbers of hair follicles, sebaceous glands and eccrine sweat glands were manually counted per mm length of epidermis. The mean values for epidermal thickness were calculated for each area and plotted on a graph for statistical analysis.

Immunohistochemistry

Skin tissue sections were embedded and sectioned as described above. Sections were fixed in 4% paraformaldehyde for 5 minutes, permeabilized in 0.2% Triton (Sigma-Aldrich, St. Louis, MO, USA, ref T92841L) in PBS for 15 minutes and then blocked in blocking buffer solution containing 10% serum, 0.2% fish skin gelatin, 0.1% BSA, and 0.5% Tween-20 (all from Sigma-Aldrich, St. Louis, MO, USA) in PBS and labelled with primary antibodies diluted in blocking buffer overnight at 4°C. Sections were washed with PBS and then labelled with secondary antibodies (all from Thermo Fisher Scientific, Waltham, MA, USA) for 1 hour at room temperature, washed with PBS, and mounted with ProLong™ Gold Antifade Mountant with DAPI (Thermo Fisher Scientific, Waltham, MA, USA, ref P36931).

RNAscope

Skin tissue sections were embedded and sectioned as described above. RNAscope experiments were performed using the RNAscope Multiplex Fluorescent Detection Kit v2 (ACDBio, Newark, California, USA, cat. no. 323100) according to the manufacturer's instructions. RNA integrity on the skin sections was assessed by RNAscope with housekeeping control probes (high (UBC), medium (PPIB) and low (POLR2A) expressors). Probes against targeted human mRNA molecules were used (all from ACDBio catalog probes, Newark, California, USA). Opal dyes (Akoya Biosciences, Marlborough, Massachusetts, USA) were used at a dilution of 1:1,000 for the fluorophore step to develop each channel: Opal 520 Reagent Pack (FP1487001KT), Opal 570 Reagent Pack (FP1488001KT) and Opal 650 Reagent Pack (FP1496001KT). Nuclei were counterstained with 4',6-diamidino-2-phenylindole and mounted using ProLong Gold Antifade Mountant (ThermoFisher, Canoga Park, California, cat. no. P36930). Slides were imaged with a Nikon A1 upright confocal microscope (Nikon, Tokyo, Japan) using a 20 dry lens and were further processed using Fiji software (62).

Optical Coherence Tomography Imaging

In vivo two-dimensional (2D) and three-dimensional (3D) angiographic optical coherence tomography (OCT) images were acquired using a commercially available OCT scanner (VivoSight Dx; Michelson Diagnostics, Kent, UK) on 16 healthy individuals and 11 patients. Images were obtained by placing the hand-held probe directly on the skin (scan area : 6 mm × 6 mm) using a fitted plastic spacer for stability and carefully avoiding compression of the skin. A full scan took 2 minutes. Angiographic OCT scanning generated greyscale images showing the structure of the skin and red areas show the vasculature based on blood flow motion in dermal vessels detected by angiographic OCT acquisition (SI Appendix, Fig. 1B). 3D reconstructions from 500 image frames were generated using Imaris software version 9 (Oxford instrument) for all samples. Total vascular density was analysed using the surface area modality in Imaris software version 9. Papillary and reticular vascular density were calculated using the threshold of mean pixel value in binarized max projection OCT frames.

Tissue dissociation and single cell RNA sequencing (scRNAseq)

Whole skin biopsies were kept on ice immediately after resection for no longer than 2hrs before processing. Characteristics of healthy donors and BCC patients (conditions, location, age, gender, number of cells) are summarised in SI Appendix, Fig. 2A. Biopsies were either directly processed for

dissociation into cell suspension or kept in MACS Tissue Storage Solution (Miltenyi Biotec, Surrey, U.K, cat. no. 130-100-008) overnight. The enzymatic and mechanical dissociation of whole skin biopsies was performed using the Whole Skin Dissociation kit for human material (Miltenyi Biotec, Surrey, U.K, cat. no. 130-101-540). Larger specimens were reduced in size by pre-cutting into several small pieces prior to enzymatic digestion and the quantity of enzymes used was adjusted according to the size of the sample. Biopsies were incubated with the enzymes for 3 hours in a shaking water bath at 37°C. Cell suspensions were then filtered through a 70µm cell strainer and centrifuged at 1500rpm for 10 minutes at 4°C. The collected cells were then frozen in 10%DMSO in Fetal Bovine Serum (FBS) and stored in liquid nitrogen.

To enrich epithelial populations, we microdissected pilosebaceous unit (PSU) cells from a healthy scalp skin donor. After treating the skin with Dispase (Sigma, Gillingham, Dorset, U.K.) overnight at 4°C, PSUs were microdissected under a dissecting stereomicroscope. PSUs and residual epidermis (IFE) were then separated into two tubes and digested with a mixture of trypsin-EDTA (Gibco, Paisley, U.K) and Versene (Gibco, 1.33:1) for 5 minutes at 37°C. Cold DMEM with 10% FBS was added and the suspension was then passed sequentially through 70µm cell strainers in Falcon tubes. The collected cells were then frozen.

For library preparation, cells were thawed and resuspended in PBS with 2% FBS and DAPI (0.05mg/ml). Dead cells were removed through flow cytometry of the final cell suspension. Immediately after flow cytometry, ~20,000 to 100,000 live single cells were loaded onto a Chromium chip and libraries were prepared through droplet encapsulation on the Chromium controller (10X Genomics; Pleasanton, CA, USA) using the Single Cell 3' reagent kits following the manufacturer's protocol. Library quantification was performed using the Qubit dsDNA HS Assay Kit (Life Technologies, Waltham, MA, USA), and cDNA integrity was assessed using D1000 ScreenTapes (Agilent Technologies). Libraries were sequenced using an Illumina HiSeq 4000 device. Using the Single Cell 3' kit provided by 10X genomics, it is estimated that approximately 10% – 20% of total transcripts are reverse transcribed.

Data Analysis of scRNAseq

Sequencing data was analysed with Cell Ranger, v6.1.1 (10X Genomics). Downstream analyses were completed with Seurat (version 4.1.1). In order to combine our sequenced facial skin cells with body skin cells, we took advantage of publicly available scRNAseq datasets from Tabib *et al.* (2) and Solé-Boldo *et al.* (3). In order to characterise the epithelial populations of human skin, we combined our

dataset from PSUs and IFE with a publicly available healthy scalp skin dataset (4). These datasets were combined using the merge() function from Seurat (Satija Lab, New York Genome Center, New York) and then a Seurat object was created with "min. cells = 3" and "min.features = 200." Cells with more than 5,000 expressed genes were removed to eliminate possible cell doublets, and cells with more than 5% mitochondrial reads were discarded to eliminate apoptotic cells. Seurat reference-based integration protocols were applied, which permitted the assembly of multiple, distinct single-cell RNA-sequencing data sets into an integrated reference and the correction of batch effects from interindividual differences. First, preprocessing of the data included log normalization of the unique molecular identifier counts and identification of the 2,000 most variable genes per sample. Then, using the FindIntegrationAnchors() function with default parameters and 20 canonical correlation analysis dimensions, integration anchors were identified across all samples. These anchors were used to integrate the data (using the IntegrateData() function, with the first 20 correlation analysis dimensions and default parameters). The integrated data were used for cell clustering and visualization with Seurat, which used the 2,000 most variable genes of the integrated data set as input. Next, data were scaled using the ScaleData() function. Principal component analysis (PCA) dimensions were calculated with the RunPCA() function. Unsupervised clustering of the data was performed with the FindNeighbors() and FindClusters() functions. The first 20 PCA dimensions were used for the FindNeighbors() function to construct a shared nearest-neighbour graph of all the data. Then, the cells were clustered using the function FindClusters() with a shared nearest-neighbour modularity optimization-based clustering algorithm with a resolution of 0.65. Finally, the RunUMAP() function was used with default parameters and 20 PCA dimensions for visualization. FindAllMarkers() uses a Wilcoxon rank-sum test to identify the representative genes of each cluster. These representative genes were used to establish the cell identity of each cluster, in addition to well-known markers defined in the literature for human skin cell types. The average expression of a particular set of marker genes was used for cell type identification and projected into a Uniform Manifold Approximation and Projection (UMAP).

Differentially expressed genes were identified using the Wilcoxon sum rank test with a fold change cut-off of 0.25 (natural log scale) and only positive markers were returned. Genes that were detected in a minimum of 10% of cells in either of the two compared clusters were used.

Gene ontology (GO) term enrichment analysis was performed using ShinyGO 0.77 (5), inputting all significantly differentially expressed genes and assessing enrichment of Biological Process GO terms.

Putative cell-cell interactions were identified using CellPhoneDB (version 3.0.2; database v4.0.0) (6). CellPhoneDB uses a curated database of ligands, receptors and their interactions to identify ligand-receptor pairs that are highly expressed, and exhibit cell type specificity. We used fine-resolution cell types and “statistical_analysis” methods to identify the cell type-specific interactions. Interactions with p values of 0.05 or less, and mean normalized expression of 0.5 or higher, were considered in the further analysis. Microenvironments defined from the spatial transcriptomics experiments were used to refine the results of the analysis. Custom visualisations of specific interacting cell type pairs were generated using R/ggplot2.

We performed pseudotemporal trajectory and pseudotime using Monocle 3 v0.1.3 (7). The UMAP space from the Seurat package was used as an input of the reduced dimensional function in Monocle 3.

Spatial transcriptomics (10X Visium)

Fresh frozen OCT-embedded skin biopsies were cryosectioned at 10µm, placed onto Visium slides (10X Genomics) and processed according to the manufacturer’s protocol. The full list of healthy donors and BCC patients (conditions, location, age, gender, number of cells) are summarised in SI Appendix, Fig. 4A. Prior to library generation, optimal RNA integrity on the skin sections was assessed by RNAscope using three housekeeping genes with high (UBC) and medium (PPIB) expression (SI Appendix, Fig. 4B). We also validated the integrity of the Visium library after sequencing: all samples presented good RNA quality (SI Appendix, Fig. 4C). However, we excluded 2 of the 32 10X Visium samples from further analysis due to poor tissue attachment to the slide.

Visium spatial gene expression slides and reagents were used according to manufacturer instructions (10X Genomics). Optimal permeabilization time for 10 µm skin sections was 20 minutes. cDNA libraries were quality controlled using the Agilent Bioanalyser. The cDNA libraries were sequenced on the Illumina HiSeq 4000 system, targeting 300 million reads per section with parameters 28cy R1, 8cy i7 index, 0cy i5 index, 91cy read 2. For each frozen section, FASTQ files were manually aligned with corresponding H&E images and then analyzed with Space Ranger version 1.3.0 which uses the STAR genome aligner version v.2.5.1b and the human reference genome: Homo_sapiens (1000Genomes_hs37d5 + ensembl_75_transcriptome) [star] and Homo_sapiens (GRCh38_15_plus_hs38d1 + ensembl_90_transcriptome) [star].

Data Analysis of 10X Visium Samples

Space ranger outputs were imported into R via Seurat V.4.1.1. All samples were merged to enable a comprehensive analysis. Spots with more than 30% of mitochondrial genes and less than 200 genes per spot were filtered out. Additionally, necrotic or damaged areas were identified by a dermatologist experienced in the interpretation of skin frozen sections (M.D.L) and excluded from further analysis.

Raw counts were normalized with the SCTransform() function. Dimensionality and clustering steps were the same as in the standard scRNAseq workflow. Data were visualised in UMAP space using DimPlot() or overlaid on the H&E images using SpatialDimPlot() or SpatialFeaturePlot() functions. In order to deconvolute scRNAseq clusters into 10X Visium sections, we used *cell2location v0.1* (8). This algorithm comprises two steps. First, we trained a negative binomial regression model to estimate reference transcriptomic profiles for all the cell types profiled with scRNA-seq in the organ. We excluded very lowly expressed genes using the filtering strategy recommended by cell2location authors (cell_count_cutoff=5, cell_percentage_cutoff SFRP2+ fibroblasts=0.03, nonz_mean_cutoff=1.12). Donor information was included as a categorical covariate. Training lasted for 250 epochs and reached convergence according to manual inspection. Next, we estimated the abundance of cell types in the ST slides using reference transcriptomic profiles of different cell types. All slides were analysed jointly. The following cell2location hyperparameters were used: (1) expected cell abundance (N_cells_per_location) = 30; (2) regularisation strength of detection efficiency effect (detection_alpha) = 20. The training was stopped after 50,000 iterations. All other parameters were used at default settings. Cell2location estimates the posterior distribution of cell abundance of every cell type in every spot. Posterior distribution was summarised as 5% quantile, representing the value of cell abundance for which the model has high confidence.

To identify microenvironments of colocalizing cell types, we used non-negative matrix factorisation (NMF). We first normalized the matrix of estimated cell type abundances by dividing it by per-spot total abundances. The resulting matrix Xn of dimensions $n \times c$, where n is the total number of spots in the Visium slides and c is the number of cell types in the reference, was decomposed as Xn = WZ, where W is a $n \times d$ matrix of latent factor values for each spot and Z is a $d \times c$ matrix representing the fraction of abundance of each cell type attributed to each latent factor. Here latent factors correspond to tissue microenvironments defined by a set of colocalized cell types. We used the NMF package (9) for R, setting the number of factors d = Hi NN and using the default algorithm (10). NMF coefficients were normalized by a per-factor maximum. We ran NMF 100 times and constructed the coincidence

matrix. Then we selected the best run based on lower mean silhouette calculated on coincidence matrix. If more than one run had minimal mean silhouette, we selected the one with smaller deviance (as reported by nmf function).

For celltype abundance correlation analysis we used per-spot normalized Xn matrix. PCC was calculated for each celltype pair and each sample.

In situ sequencing (ISS)

Fresh frozen OCT-embedded skin biopsies were cryosectioned as 10µm sections and placed onto SuperFrost Plus glass slides (Thermo Fisher Scientific, Waltham, MA, USA) and stored for less than a week at -80˚C prior to high sensitivity library preparation. Sections were fixed with 4% formaldehyde (Merck, Gillingham, Dorset, U.K, ref. F1635) for 30 minutes and then permeabilized for 90 seconds with 0.1 mg/mL pepsin (Merck, Gillingham, Dorset, U.K, ref. 10108057001) in 0.1M HCl (VWR, Leicestershire, U.K, ref. 20255.290). RNA integrity and assay conditions were assessed using MALAT1 (high expressor) and RPLP0 (medium expressor) housekeeping genes only (SI Appendix, Fig. 4E). For library preparation, chimeric padlock probes (targeting directly RNA and containing an anchor sequence as well as a gene-specific barcode) for a custom panel of 81 genes (SI Appendix, Table 2) as well as of 90 genes from 2 pre-defined immune panels (SI Appendix, Table 3, 4) were hybridized overnight at 37°C, then ligated before the rolling circle amplification was performed overnight at 30°C using the High Sensitivity Library Preparation kit for CARTANA technology (10xGenomics) following the manufacturer’s instructions. All incubations were performed in SecureSealTM chambers (Grace Biolabs, Bend, OR, USA). To quench autofluorescence background, TrueView (SP-8400 VectorLabs) was used for 2 minutes at room temperature. Slow Fade Antifade Mountant (Thermo Fisher Scientific, Waltham, MA, USA, ref S36936) was used for optimal handling and imaging of tissue sections. Quality control of the library preparation was performed by applying anchor probes to detect simultaneously all rolling circle amplification products from all genes in all panels. Anchor probes were labelled probes with Cy5 fluorophore (excitation at 650 nm and emission at 670 nm).

All samples passed quality control and were sent to CARTANA Sweden (a subsidiary of 10X Genomics), for *in situ* barcode sequencing, imaging and data processing. Briefly, adapter probes and sequencing pools (containing 4 different fluorescent labels: Alexa Fluor® 488, Cy3, Cy5 and Alexa Fluor® 750) were hybridized to the padlock probes to detect the gene-specific barcodes, through a sequence specific signal for each gene specific rolling circle amplification product. This was followed by imaging. Six iterations were performed to permit decoding of all genes in the panel. Raw data consisting of 40x

images from 5 fluorescent channels (DAPI, Alexa Fluor® 488, Cy3, Cy5 and Alexa Fluor® 750) were taken as z-stacks and flattened to 2D using maximum intensity projection. After image processing and decoding, the results were summarized in a csv file and gene plots were generated using MATLAB.

Data Analysis for ISS

Analysis of ISS data was performed for each sample following the SSAM pipeline (v.1.0.2) (11) with minor modifications to the code. Gene reads were loaded and their coordinates transformed from pixels into micrometers (0.16 µm per pixel) to create a SSAM dataset object. The locations of the gene reads were converted into mRNA density through a Kernel Density Estimation (KDE) using a Gaussian kernel and a bandwidth of 2.5. Local maxima of the mRNA densities were found using a total gene expression threshold of 0, a per gene expression threshold of 0, and a search size of 3. Local maxima were employed to calculate the variance stabilization parameters with the sctransform package (v0.3.4) (12), which were subsequently used for gene expression normalization. Cell type identification was performed following SSAM guided analysis and using either the merged face and body or face and BCC processed scRNAseq data as the reference dataset. The raw gene counts for both reference datasets were normalized using sctransform and the average gene expression per cell type was calculated. Local maxima vectors were mapped to the cell type clusters with the most similar gene expression in the scRNAseq reference dataset, using a correlation threshold of 0.15 and a threshold of vector normalisation of 0.0015.

Key Resources Table

Reagent or Resource	Source	Identifier
**Antibodies**		
Cytokeratin 17 Monoclonal Antibody (E3)	Thermo Fisher Scientific	MA5-13539
CLDN5 polyclonal antibody	ABCAM	AB131259
Donkey anti-rabbit IgG (H+L) Alexa Fluor Plus 647	Invitrogen	A-32795
Donkey anti-mouse IgG (H+L) Alexa Fluor Plus 594	Invitrogen	A-32744
Goat anti-chicken IgY (H+L) Alexa Fluor 647	Invitrogen	A-21449
Goat anti-mouse IgY (H+L) Alexa Fluor 647	Invitrogen	A-11001
Vimentin monoclonal antibody	Leica	NCL-L-VIM-572
TAGLN polyclonal antibody	Abcam	ab10135
RGS5 polyclonal antibody	Abcam	ab196799
CD146 (NCAM) Mouse monoclonal	Abcam	ab24577
**Critical Commercial Assays**		
Chromium Single Cell 30 Library & Gel Bead Kit v2	10x Genomics	PN-120267
RNAscope® Multiplex Fluorescent Reagent Kit	Bio-Techne	323100
Opal 520 Reagent Pack	Akoya Biosciences	FP1487001KT
Opal 570 Reagent Pack	Akoya Biosciences	FP1488001KT
Opal 650 Reagent Pack	Akoya Biosciences	FP1496001KT
**RNAscope probes**		
HS-ACTA2	Bio-Techne	311811

HS-APOD	Bio-Techne	445171
HS-DES	Bio-Techne	403041
HS-POSTN	Bio-Techne	409181
HS-PPIB - RNAscope® 3-plex Positive Control Probe	Bio-Techne	320861
HS-PTGDS	Bio-Techne	431471
HS-SFRP2	Bio-Techne	476341
Hs-UBC - RNAscope® 3-plex Positive Control Probe	Bio-Techne	320861
**Software and Algorithms**		
Cell2location	(8)	https://github.com/BayraktarLab/cell2location
Cell Ranger Analysis Pipeline v6.1.1	10X Genomics	https://10xgenomics.com/
CellPhoneDB	(6)	https://github.com/ventolab/CellphoneDB/
ShinyGO 0.77	(5)	http://bioinformatics.sdstate.edu/go/
MATLAB R2022a	MathWorks	https://mathworks.com/
MATLAB script to decode intensity information in situ sequencing data	(13)	https://github.com/Moldia/in_situ_seq
sctransform v0.3.4	(12)	https://github.com/satijalab/sctransform
Seurat v4.1.1	(14)	https://satijalab.org/seurat/install.html
Monocle 3	(7)	https://github.com/cole-trapnell-lab/monocle3
Space Ranger Analysis Pipeline v1.3.0	10X Genomics	https://10xgenomics.com/
SSAM v1.0.2	(11)	https://github.com/HiDiHlabs/ssam

Additional Acknowledgements

We thank Marina de Paula-Silva and Christina Philippeos at King's College London at KCL for their help with skin sample collection and sectioning. We thank Shahnawaz Ali, Kalle Sipilä, Matteo Vietri Rudan and Miguel Bernabé-Rubio at KCL and Anais Julien at the Karolinska institutet, Sweden, for helpful discussions. We thank Christos Tziotzios for collaboration in sourcing skin scalp samples. We thank Eva Wozniak and Paul Stevens at the Genome Centre, Blizard Institute, QMUL, and Han Lu and Dasha Freydina at the Genomics Centre, KCL, for assistance with library preparation. We thank the genomic platform and the cellular genetics informatics team at the Wellcome Sanger Institute, Hinxton, in particular Elena Prigmore, Stijn van Dongen and Steven Leonard for sequencing the libraries. We thank Morgane Rouault, Dabashish Chitnis and Hao Xu at 10X genomics, Sweden, for the sequencing and decoding for our ISS slides. We thank staff at the Advanced Sequencing Facility at the Francis Crick Institute, particularly Robert Goldstone and Amelia Edwards. We thank all members of the Dermatological Surgery Unit, Guy's and St Thomas' NHS Foundation Trust, in particular Raj Mallipeddi, Faisal Ali, Jack Mann and Clare Kiely, for assistance with recruitment of patients. Finally, we would like to thank all the patients who contributed skin samples, without which this project would not have been possible.

Supplementary Figure Legends

Supplementary Figure 1

Variation in skin morphology and skin vasculature across anatomical sites

A) Histological sections of body (upper panel) and facial (lower panel) healthy skin (H&E stain, representative examples are shown).

B) Quantification of differences in epidermal thickness and density of sebaceous glands, hair follicles and eccrine sweat glands per mm length of epidermis. Significant differences (One way ANOVA-test) are indicated (**<0.01,****<0.0001).

C) Optical coherence tomography (OCT) imaging of skin microstructure *in vivo* is compared for body (upper panel) and facial (lower panel) skin. The intensity of the skin reflectance is shown in greyscale and speckle contrast imaging of blood vessels in red.

D) Quantification of skin vasculature density across a total of 6 anatomical sites: 3 from the body (Abdomen, Forearm, Back) and 3 from the face (Forehead, Nose and Ear) across 16 individuals (upper panel). Comparison of vasculature density in papillary and reticular dermis across a total of 6 anatomical sites (lower panel). Each filled circle represents the result of a single observation. Statistically significant differences (One way ANOVA-test) are indicated (* <0.05, **<0.01, ***<0.001, ****<0.0001).

E) Further vasculature network architecture quantification was performed on face skin areas only, because the vascular network was continuous, permitting quantification of segment length, number of segments, tube thickness and number of branching points. No statistically significant differences were found (One way ANOVA-test).

Supplementary Figure 2

Single cell RNA sequencing of human healthy skin and BCC

A) The source and demographics of the skin samples incorporated into our combined single cell RNA sequencing dataset are described in this table.

B) UMAP plot illustrating integrated single cell sequencing from all the donors. Color indicates the donor source of the cell.

C) Quality control plots showing percent of UMI counts in mitochondrial genes per scRNAseq sample (upper panel) and frequency distribution of UMI counts (log1p-transformed) per scRNAseq sample (lower panel).

D) UMAP plots showing the contribution of each condition (BCC from the face, healthy face and body) to the distinct clusters identified in the integrated dataset. Color indicates the identity of the scRNAseq clusters.

E) Dot Plot showing marker genes specific to each skin cluster. For each cluster, the percentage of cells expressing the marker (diameter) and the average log2 normalized expression (color) is shown.

F) Quantification of chondrocytes (upper panel) and skeletal muscle cells (lower panel) according to facial anatomical sites. Since chondrocytes were only detected in an ear skin sample and skeletal muscle cells in a forehead skin sample statistical significance could not be calculated.

Supplementary Figure 3

Developmental gene signatures in human skin cells vary across anatomical sites

A) Expression of selected somatic mesoderm Hox genes (upper panel) and neural crest mesenchymal genes (lower panel) in our integrated scRNAseq are illustrated in dot plots for which the diameter of the circle corresponds to the percentage of cells expressing the gene and the color reflects the average log2 normalized expression.

B) Average expression of selected somatic mesoderm Hox genes (upper panel) and neural crest mesenchymal genes (lower panel) in global ST profiling.

C, D) Expression of selected neural crest mesenchymal genes in scRNAseq data according to cell type (C) and expression of selected somatic mesoderm Hox genes in scRNAseq data according to cell type (D). Average log2 normalized expression for each cell type is compared between body (blue), face (red) and BCC (green) conditions. Hox genes were preferentially expressed in body fibroblasts, pericytes, SMC, chondrocytes and VEC. Mesenchymal neural crest genes were preferentially expressed in facial (healthy and BCC) fibroblasts, pericytes, SMC and chondrocytes. Other populations, including melanocytes, Schwann cells and keratinocytes, exhibited less striking differences in marker expression between body, face and BCC face. Skin immune cell populations did not exhibit differential expression of these genes.

Supplementary Figure 4

Spatial transcriptomics and *in situ* sequencing of human healthy skin and BCC

A) The source and demographics of the skin tissue sections used for ST (10X Visium) (black and red) and ISS (red) are described in this table.
B) Multiplex RNAscope of housekeeping gene expression (*PPIB* and *UBC*) was performed on all the skin sections selected for ST by 10X Visium in order to check the presence of high quality RNA throughout tissue sections. One representative sample is shown (healthy face sample).
C) Quality control plots showing percent of UMI counts in mitochondrial genes per ST sample (upper panel) and frequency distribution of UMI counts (log1p-transformed) per ST sample (lower panel).
D) UMI counts in body, face and BCC face ST skin sections used for 10X Visium protocol. Three representative samples are shown.
E) Prior to final library preparation for ISS, RNA integrity and assay conditions were assessed using *MALAT1* (high expressor, not shown) and RPLP0 (medium expressor, shown here) housekeeping genes. Three representative samples are shown.
F) Skin specific 165 gene panel in all the sections used for ISS. Each gene is represented by different color dots. Three representative samples are shown.

Supplementary Figure 5

Mapping of the main human skin cell types using global spatial transcriptomics and targeted *in situ* sequencing

A) Using the cell2location pipeline, scRNAseq clusters corresponding to the epidermis (suprabasal & basal keratinocytes), dermal stroma (fibroblasts) and endothelium (endothelial cells, pericytes and SMC) were projected onto the global ST skin sections isolated from the same skin biopsies.
B) Three representative ISS images from body, face and BCC face representing the main cell types in human skin, highlighting tissue architecture and demonstrating the sensitivity of ISS technology. *KRT14, KRT5, KRT10* and *KRT1* marked the epidermis and healthy epithelial structures and tumours within the dermis (blue). *EMB, MGST1, KRT19* and *DCD* marked the appendages such as sebaceous glands and sweat glands (pink). *DCN, COL1A1* and *PDGFRA* marked the dermal stroma (green). *PECAM, CLDN5, ACKR1* and *ACTA2* marked the dermal stroma. DAPI labelled nuclei (gray).
C) Using the SSAM pipeline, the same scRNAseq clusters with the same color code were projected onto the same ISS skin sections demonstrating single cell spatial mapping.

Supplementary Figure 6

Additional analysis of pericyte populations in healthy skin and BCC

A) OCT imaging of vascular network of human BCC *in vivo*. En-face view showing speckle contrast imaging of blood vessels in red via 3D computational reconstructions. Image size 6 mm × 6 mm; scale bar: 500 µm.

B) Gene ontology terms of different peri-vascular subpopulations (*RGS5*+ pericytes, *TAGLN*+ pericytes, SMC).

C) Expression of markers specific to the different pericyte subpopulations in global ST sections of human skin from face and body areas and BCC. Three representative samples are shown.

D) Spatial localisation of ISS reads specific to the different pericyte subpopulations in sections of human skin from face and body areas and BCC. Three representative samples are shown.

E) Heatmap showing the total number of interactions between *RGS5+, TAGLN+* pericytes, SMC and VEC in the scRNAseq dataset obtained with CellPhoneDB, a package looking for ligand–receptor interactions in scRNAseq data in combination with predicted microenvironment from ST data (methods). Scale ranges from dark-blue (0 interactions) to dark red (the maximum number interactions found; > 100).

F) Dotpots representing selected ligand–receptor interactions between pairs of *RGS5*+ pericytes, *TAGLN*+ pericytes and VEC. Only the interaction pairs that were most likely based on predicted microenvironment from ST data are shown. Results are not symmetrical; for example: *RGS5*+ pericytes | VEC with *CCL2_ACKR1* means CCL2 ligand is expressed in *RGS5*+ pericytes, and *ACKR1* receptor in VEC; for VEC | *RGS5*+ pericyte pair, the roles are switched. Thus, each pair is present twice. The total number of interactions found were filtered compared to heatmaps, and only include interactions for which at least one pair of cell types had mean expression of >= 0.5.

Supplementary Figure 7

Additional analysis of fibroblast populations in healthy skin and BCC

A) Gene ontology terms of different fibroblast subpopulations (*APOD*+ fibroblasts, *SFRP2*+ fibroblasts, *PTGDS*+ fibroblasts, *POSTN*+ fibroblasts).

B) Expression of markers specific to the different fibroblast subpopulations in global ST skin sections of human skin from face and body areas and BCC. Three representative samples are shown.

C) Spatial localisation of *ISS* reads specific to the different fibroblast subpopulations in sections of human skin from face and body areas and BCC. Three representative samples are shown.

D) Heatmap showing the total number of interactions between *APOD*+, *SFRP2*+, *PTGDS*+, *POSTN*+ fibroblasts, VEC and Basal K in the scRNAseq dataset obtained with CellPhoneDB from scRNAseq data

in combination with predicted microenvironment from ST data (methods). Scale ranges from dark-blue (0 interactions) to dark red (the maximum number interactions found; > 150).

E) Dotpots representing selected ligand–receptor interactions between pairs of APOD+, PTGDS+, POSTN+ fibroblasts and VEC. Only the interaction pairs that are most likely based on predicted microenvironment from ST data are shown. Results are not symmetrical; for example: APOD+ fibroblasts|VEC with CCL2_ACKR1 means CCL2 ligand is expressed in APOD+ fibroblasts and ACKR1 receptor in VEC; for VEC | APOD+ fibroblast pair, the roles are switched. Thus, each pair is present twice. The total number of interactions found were filtered compared to heatmaps, and only include interactions for which at least one pair of cell types had mean expression of >= 0.5.

Supplementary Figure 8

Additional analysis of keratinocyte populations in healthy skin and BCC

A) Microdissected interfollicular epidermis (IFE, light orange) and pilosebaceous units (PSU, dark orange) from healthy scalp skin were integrated in our combined epidermal dataset. We also integrated our sequenced cells with a publicly available healthy scalp skin dataset (gray, left panel). In order to perform high resolution clustering, we used the publicly available dataset as a template for the initial steps of clustering. Quality control plots showing percent of UMI counts in mitochondrial genes and frequency distribution of UMI counts (log1p-transformed) per scRNAseq sample conditions (right panel).

B) UMAP plots showing the contribution of our previous keratinocyte clusters such as basal and suprabasal in the combined epidermal dataset.

C) UMAP plots showing the contribution of each condition (BCC from the face, healthy face and body) in the distinct clusters identified in the integrated epidermal dataset. Color indicates the identity of the scRNAseq clusters.

D) Volcano plot highlighting differential expression of PTCH1/2, HHIP (hedgehog signalling pathway), KRT17, EPCAM, IGKC and TP63 in BCC basal keratinocytes compared to healthy basal keratinocytes.

E) Prediction of epidermal cell populations in global ST skin sections of human skin (face and body) and in BCC with cell2location (Methods). Three representative samples are shown. Skin architecture (H&E) is shown in grey; signal intensity is shown by the different colors indicated in each scRNAseq cluster.

F) UMAP plots showing average log2 normalized KRT17 expression in our epidermal combined dataset.

G) Expression of KRT17 in ST skin sections of facial, body skin and BCC. Signal intensity corresponds to color. Three representative samples are shown.

H) Monocle trajectory analysis of our epidermal combined dataset.

Supplementary Figure 9

Stem cell marker expression in the epithelial cell populations in healthy skin and BCC

A) Dotplot representing the expression of selected stem cell markers in the epithelial scRNAseq dataset.

B) Differential expression of *LGR5* in the epithelial scRNAseq dataset in BCC and healthy skin (face and body).

Supplementary Table Legends

Supplementary Table 1

Top fifty differentially expressed genes within all skin and BCC cell clusters

Table showing significantly differentially expressed genes (top50) in each cluster identified through unsupervised clustering in all healthy skin cells (face and body) and BCC cells. Sorted from the highest average log2 normalized gene expression and adjusted p-value.

Supplementary Table 2

List of custom genes for ISS

List of the 81 custom panel genes, specific to skin subpopulations identified by scRNAseq, for the ISS.

Supplementary Table 3

List of predefined Immune General gene panel for ISS

List of the predefined panel Immune General (l1A 0.2) genes for the ISS.

Supplementary Table 4

List of predefined Immune Regulation gene panel for ISS

List of the predefined panel Immune Regulation (I3D 0.2) genes for the ISS.

Supplementary Table 5

Top fifty differentially expressed genes in epidermal cell clusters

Table showing significantly differentially expressed genes (top50) in each cluster identified through unsupervised clustering in epidermal cells in healthy skin (face and body) and BCC. Sorted from the highest average log2 normalized gene expression and adjusted p-value.

Supplementary References

1. J. Schindelin, *et al.*, Fiji: an open-source platform for biological-image analysis. *Nat. Methods* **9**, 676–682 (2012).

2. T. Tabib, C. Morse, T. Wang, W. Chen, R. Lafyatis, SFRP2/DPP4 and FMO1/LSP1 Define Major Fibroblast Populations in Human Skin. *J. Invest. Dermatol.* **138**, 802–810 (2018).

3. L. Solé-Boldo, *et al.*, Single-cell transcriptomes of the human skin reveal age-related loss of fibroblast priming. *Commun Biol* **3**, 188 (2020).

4. J. B. Cheng, *et al.*, Transcriptional Programming of Normal and Inflamed Human Epidermis at Single-Cell Resolution. *Cell Rep.* **25**, 871–883 (2018).

5. S. X. Ge, D. Jung, R. Yao, ShinyGO: a graphical gene-set enrichment tool for animals and plants. *Bioinformatics* **36**, 2628–2629 (2020).

6. M. Efremova, M. Vento-Tormo, S. A. Teichmann, R. Vento-Tormo, CellPhoneDB: inferring cell-cell communication from combined expression of multi-subunit ligand-receptor complexes. *Nat. Protoc.* **15**, 1484–1506 (2020).

7. C. Trapnell, *et al.*, The dynamics and regulators of cell fate decisions are revealed by pseudotemporal ordering of single cells. *Nat. Biotechnol.* **32**, 381–386 (2014).

8. V. Kleshchevnikov, *et al.*, Cell2location maps fine-grained cell types in spatial transcriptomics. *Nat. Biotechnol.* **40**, 661–671 (2022).

9. R. Gaujoux, C. Seoighe, A flexible R package for nonnegative matrix factorization. *BMC Bioinformatics* **11**, 1–9 (2010).

10. J.-P. Brunet, P. Tamayo, T. R. Golub, J. P. Mesirov, Metagenes and molecular pattern discovery using matrix factorization. *Proc. Natl. Acad. Sci. U. S. A.* **101**, 4164–4169 (2004).

11. J. Park, *et al.*, Author Correction: Cell segmentation-free inference of cell types from in situ transcriptomics data. *Nat. Commun.* **12**, 4103 (2021).

12. C. Hafemeister, R. Satija, Normalization and variance stabilization of single-cell RNA-seq data using regularized negative binomial regression. *Genome Biol.* **20**, 1–15 (2019).

13. R. Ke, *et al.*, In situ sequencing for RNA analysis in preserved tissue and cells. *Nat. Methods* **10**, 857–860 (2013).

14. A. Butler, P. Hoffman, P. Smibert, E. Papalexi, R. Satija, Integrating single-cell transcriptomic data across different conditions, technologies, and species. *Nat. Biotechnol.* **36**, 411–420 (2018).

Supplementary Figure 1

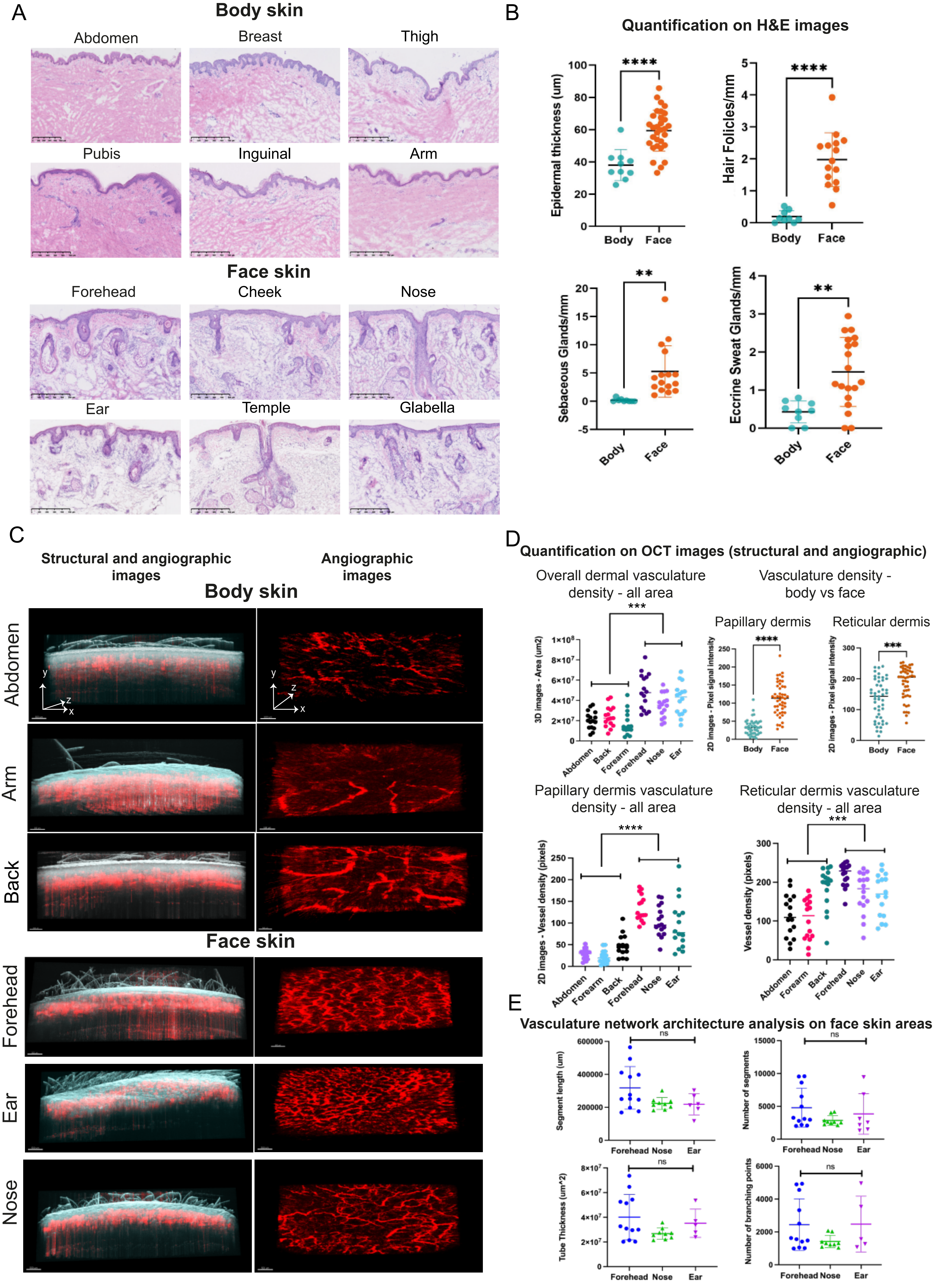

Supplementary Figure 2

A

	Name	Disease/ Healthy	Face/Body	Age	Gender	Location	Fitz-patrick scale	N of cells
Sequenced in this study	face_ear1	Healthy	Face	77	Male	Ear	I/II	6278
	face_nose1	Healthy	Face	56	Male	Nose	I/II	6618
	face_cheek1	Healthy	Face	85	Male	Cheek	I/II	6761
	face_forehead1	Healthy	Face	85	Male	Forehead	I/II	9499
	face_forehead2	Healthy	Face	70	Male	Forehead	I/II	1945
	face_cheek2	Healthy	Face	62	Male	Cheek	I/II	1177
	face_ear2	Healthy	Face	86	Male	Ear	I/II	3088
	face_temple1	Healthy	Face	59	Male	Temple	I/II	5105
	face_forehead3	Healthy	Face	80	Male	Forehead	I/II	8039
	face_temple2	Healthy	Face	78	Male	Temple	I/II	1344
	face_cheek3b	Healthy	Face	81	Female	Cheek	I/II	4010
	face_forehead4	Healthy	Face	90	Male	Forehead	I/II	6861
	face_cheek4	Healthy	Face	90	Male	Cheek	I/II	9250
	face_forehead5	Healthy	Face	73	Male	Forehead	I/II	5755
	bcc_cheek1	BCC	Face	39	Male	Cheek	I/II	16303
	bcc_cheek2	BCC	Face	85	Male	Cheek	I/II	5207
	bcc_ear1	BCC	Face	70	Male	Ear	I/II	5573
	bcc_ear2	BCC	Face	69	Male	Ear	I/II	6694
	bcc_forehead1	BCC	Face	73	Male	Forehead	I/II	10224
	bcc_nose1	BCC	Face	91	Male	Nose	I/II	6250
	bcc_temple1	BCC	Face	51	Male	Temple	I/II	6028
	bcc_temple2	BCC	Face	59	Male	Temple	I/II	2830
Publicly available	body_sole-boldo GSE130973_ filtered	Healthy	Body	25 – 70	5 male	Inguinal	n/a	14033
	body_tabib Control_Human _Skin_6People. csv	Healthy	Body	23 – 66	3 female donors & 3 male donors	Arm	n/a	6529

B

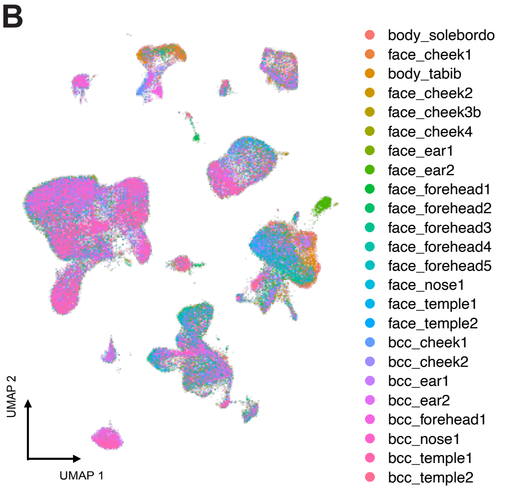

C

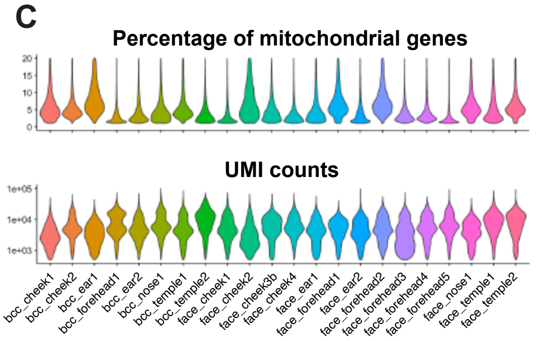

D

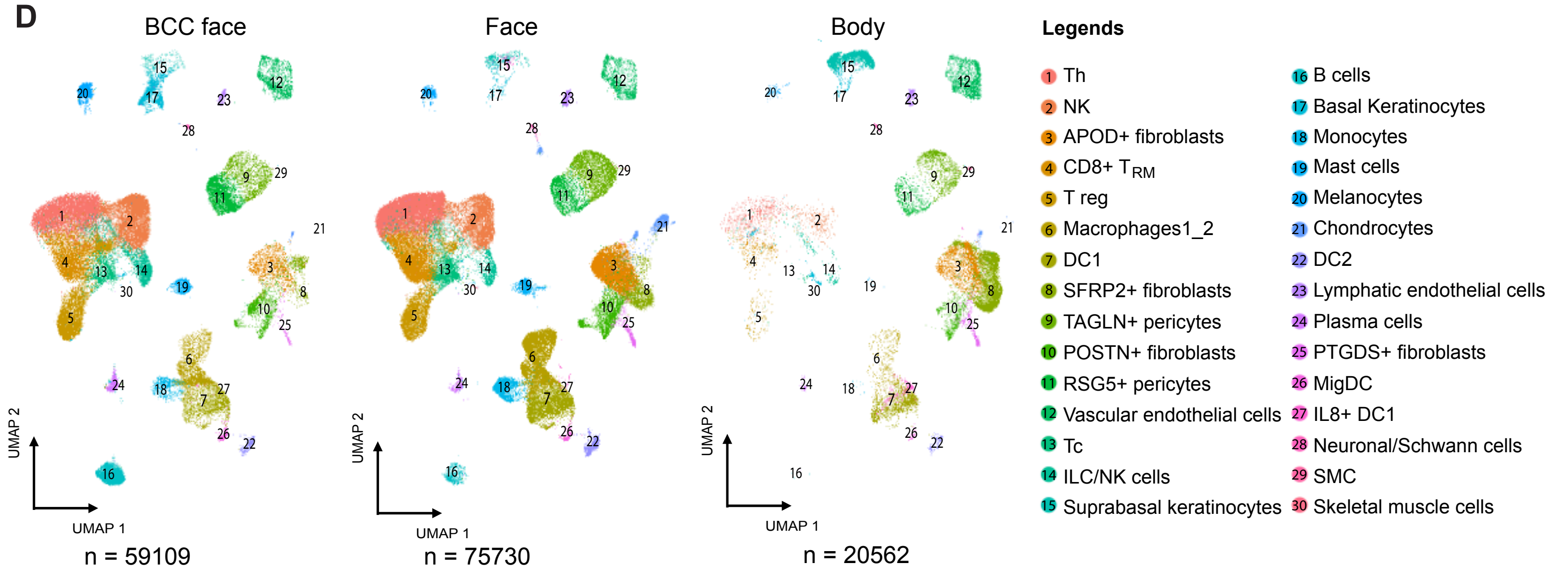

E

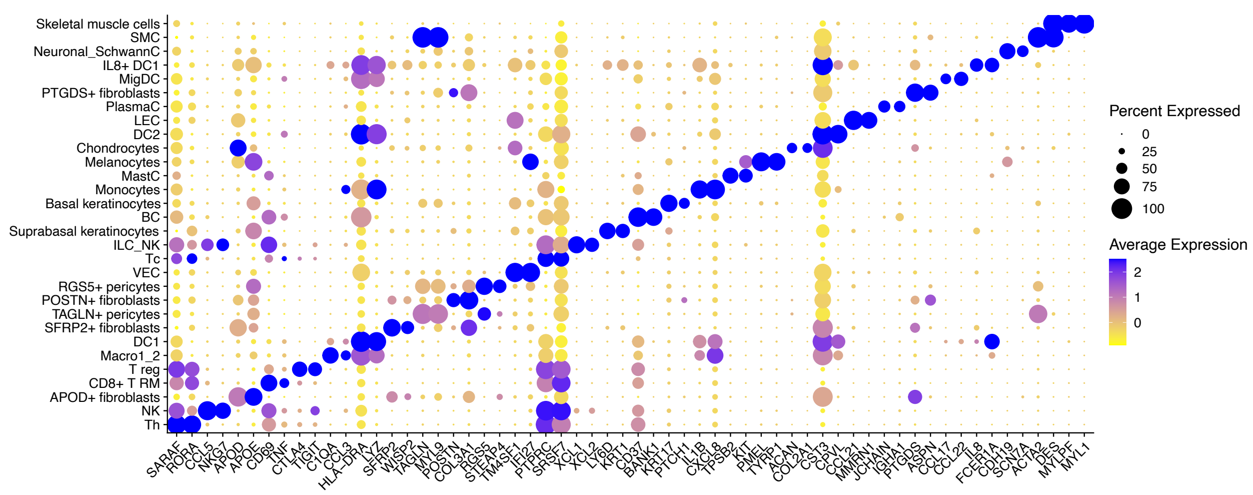

F

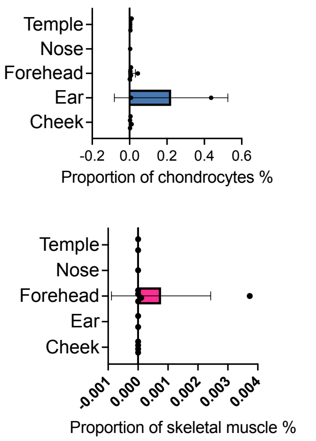

Supplementary Figure 3

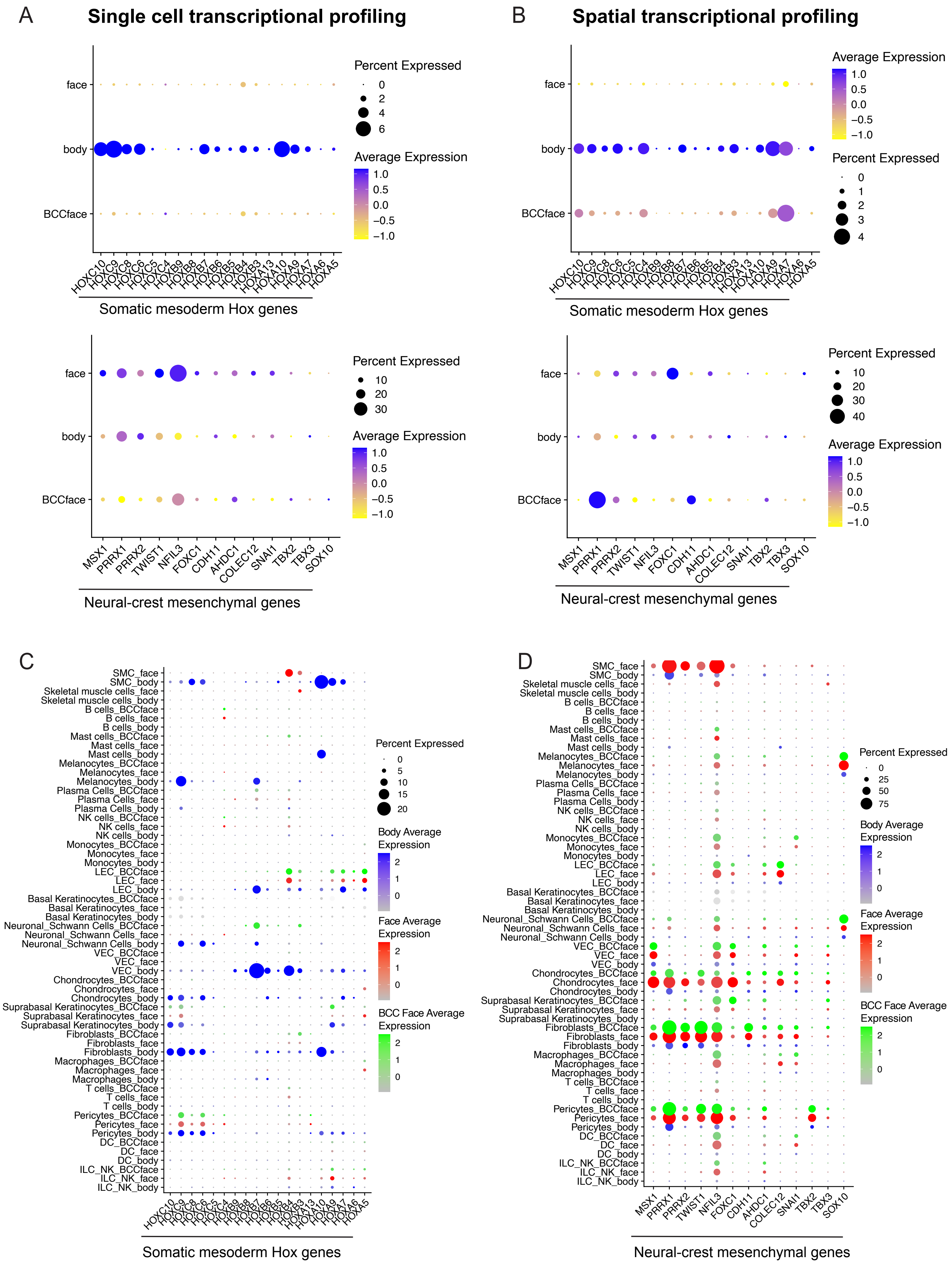

Supplementary Figure 4 :

A 10X visium and *In situ* sequencing samples

Name	Disease/ Healthy	Face/ Body	Age	Gender	Location	Fitz-patrick scale	N of spots	N of ISS reads
face_temple1a	Healthy	Face	68	Male	Temple	I/II	986	
face_temple1b	Healthy	Face	68	Male	Temple	I/II	924	
face_temple2a	Healthy	Face	33	Female	Temple	I/II	1520	
face_temple2b	Healthy	Face	33	Female	Temple	I/II	1484	
body_back1a	Healthy	Body	60	Male	Back	I/II	1424	
body_inguinal1a	Healthy	Body	55	Male	Inguinal	I/II	1297	
face_nose1a	Healthy	Face	77	Male	Nose	I/II	1546	
face_glabella1	Healthy	Face	68	Male	Glabella	I/II	1574	947395
face_forehead1a	Healthy	Face	55	Male	Forehead	I/II	1013	
face_cheek1	Healthy	Face	39	Male	Cheek	I/II	2025	1048575
body_abdomen1b	Healthy	Body	47	Male	Abdomen	I/II	1491	
body_thigh1b	Healthy	Body	53	Male	Thigh	I/II	1766	310938
body_pubis1	Healthy	Body	52	Male	Pubis	I/II	1630	469219
body_inguinal2	Healthy	Body	63	Male	Inguinal	I/II	1945	
face_forehead2	Healthy	Face	73	Male	Forehead	I/II	1957	650238
face_forehead1b	Healthy	Face	55	Male	Forehead	I/II	958	
face_nose1b	Healthy	Face	77	Male	Nose	I/II	1639	971193
face_cheek2	Healthy	Face	76	Male	Cheek	I/II	1375	
body_breast1	Healthy	Body	42	Female	Breast	I/II	2086	310513
body_back1b	Healthy	Body	60	Male	Back	I/II	2447	
body_inguinal1b	Healthy	Body	55	Male	Inguinal	I/II	2222	
face_scalp1	Healthy	Face	68	Male	Scalp	I/II	1342	
bcc_face_cheek1	BCC	Face	39	Male	Cheek	I/II	811	768408
bcc_face_forehead1	BCC	Face	73	Male	Forehead	I/II	1032	1048576
bcc_ face_cheek2	BCC	Face	85	Male	Cheek	I/II	1868	2170139
bcc_ face_nose1	BCC	Face	91	Male	Nose	I/II	1179	3001719
bcc_ face_nose2	BCC	Face	72	Female	Nose	I/II	1324	
bcc_face_ear1	BCC	Face	90	Male	Ear	I/II	1123	
bcc_face_forehead2	BCC	Face	64	Female	Forehead	I/II	907	
bcc_face_nose3	BCC	Face	82	Female	Nose	I/II	927	

B face_forehead1a

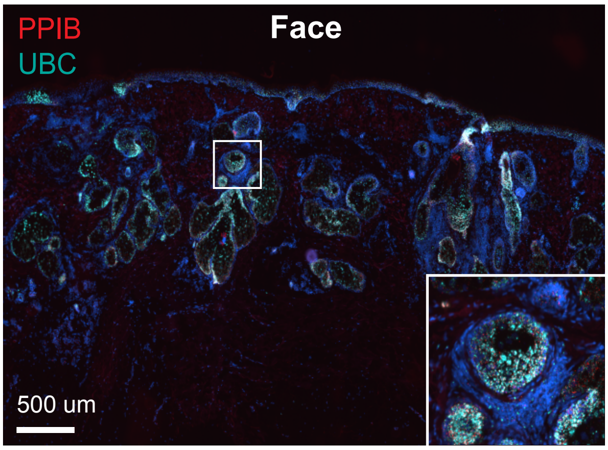

C

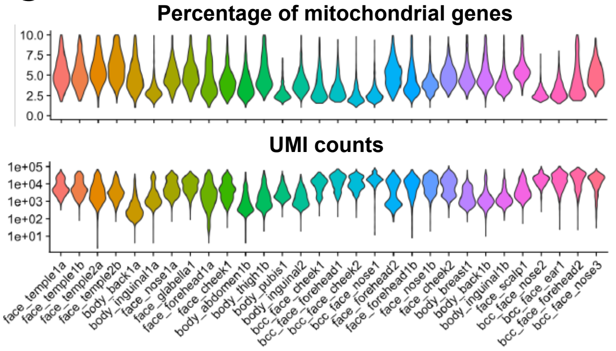

D

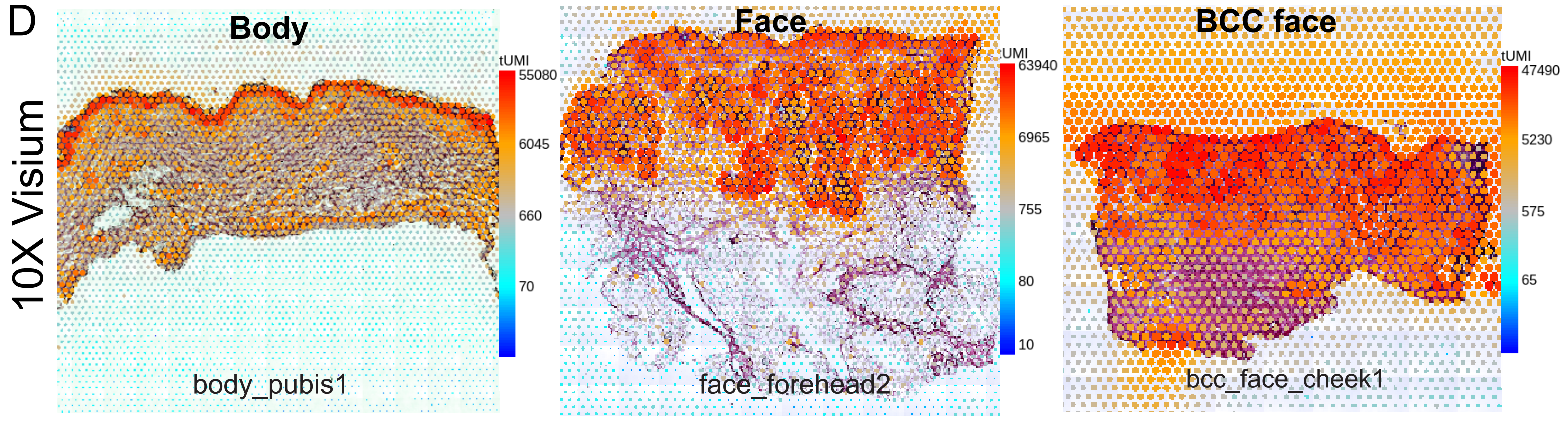

E

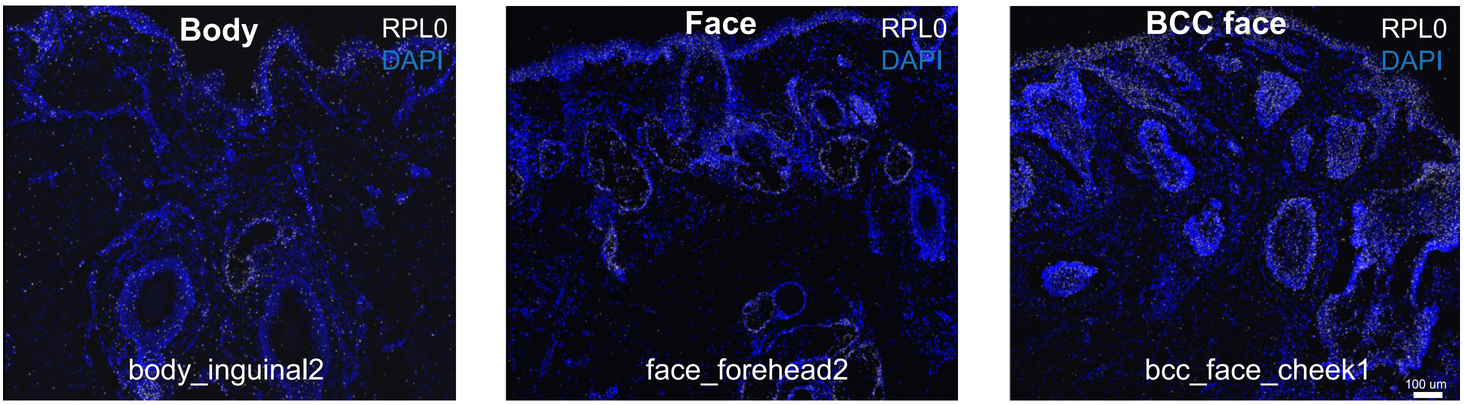

F

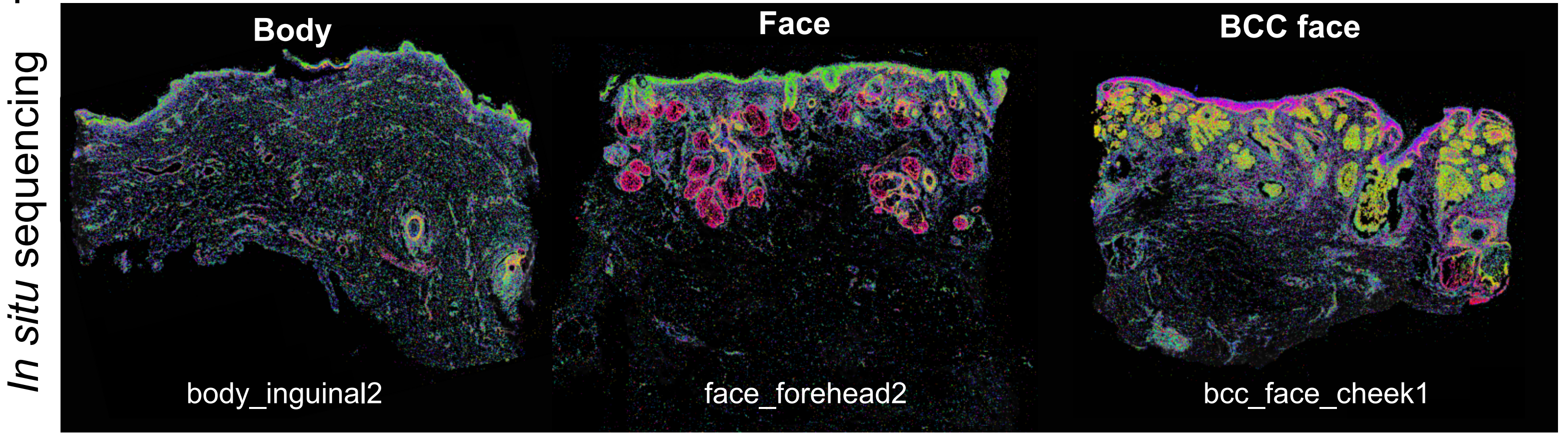

Supplementary Figure 5

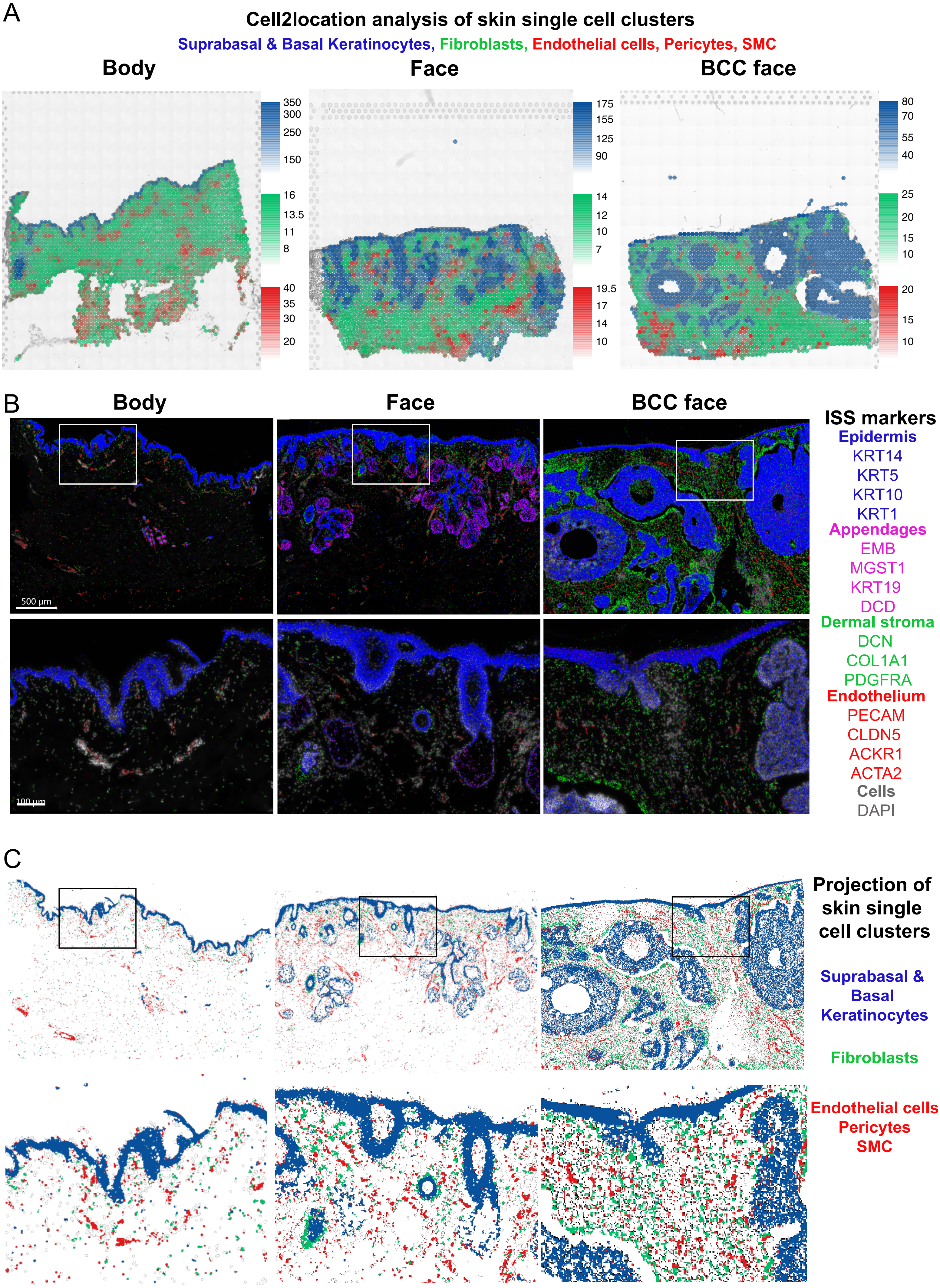

Supplementary Figure 6

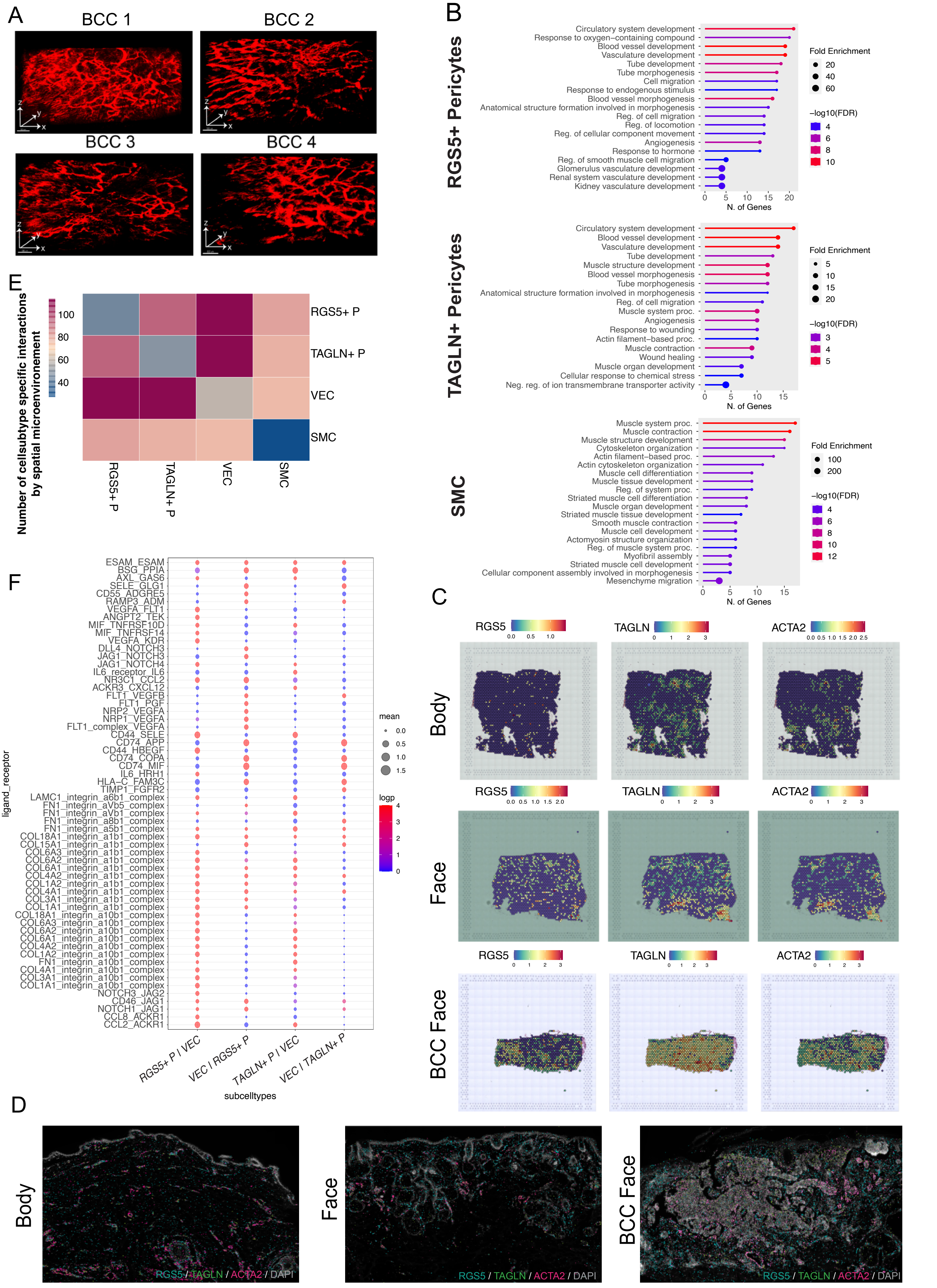

Supplementary Figure 7

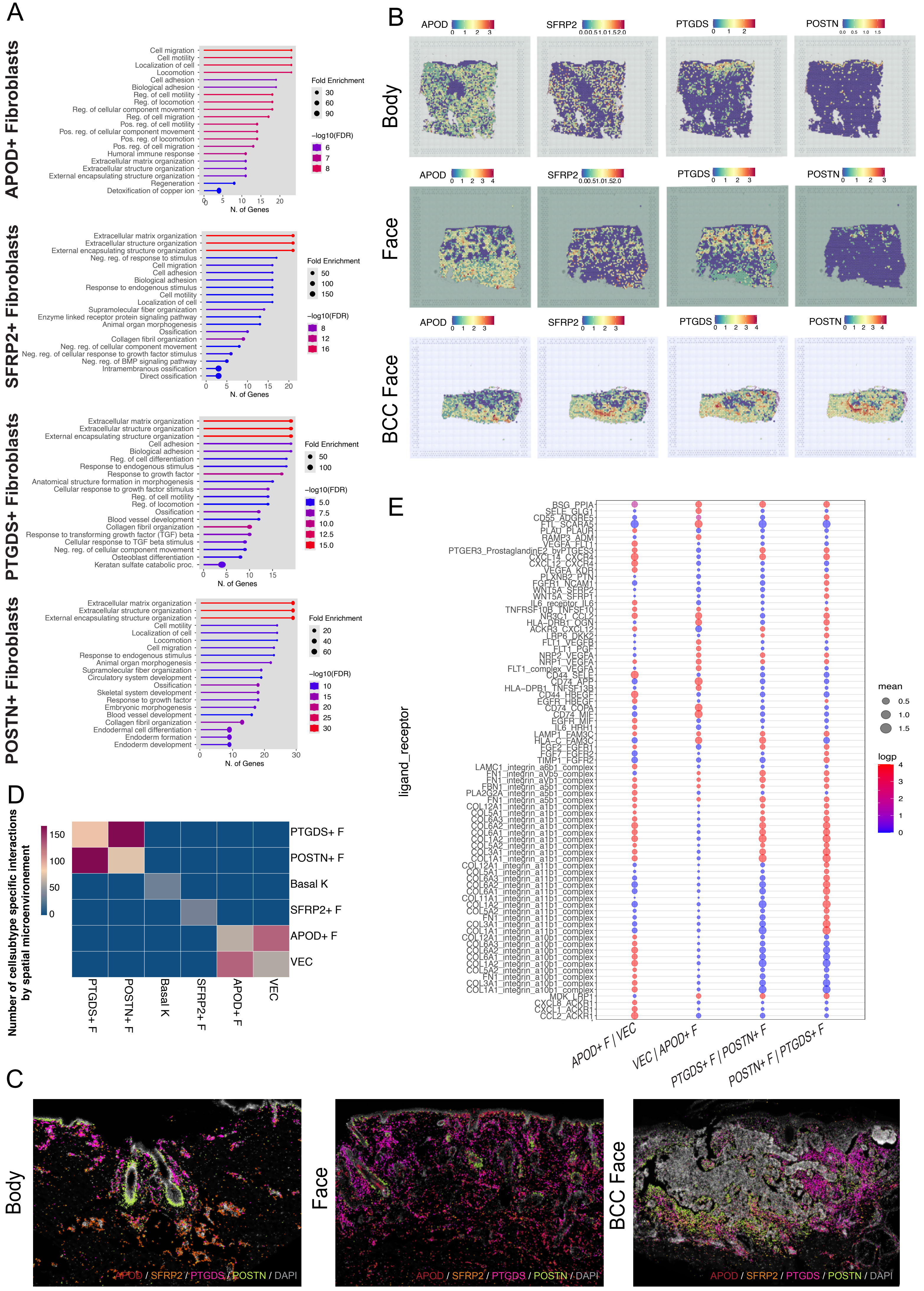

Supplementary Figure 8

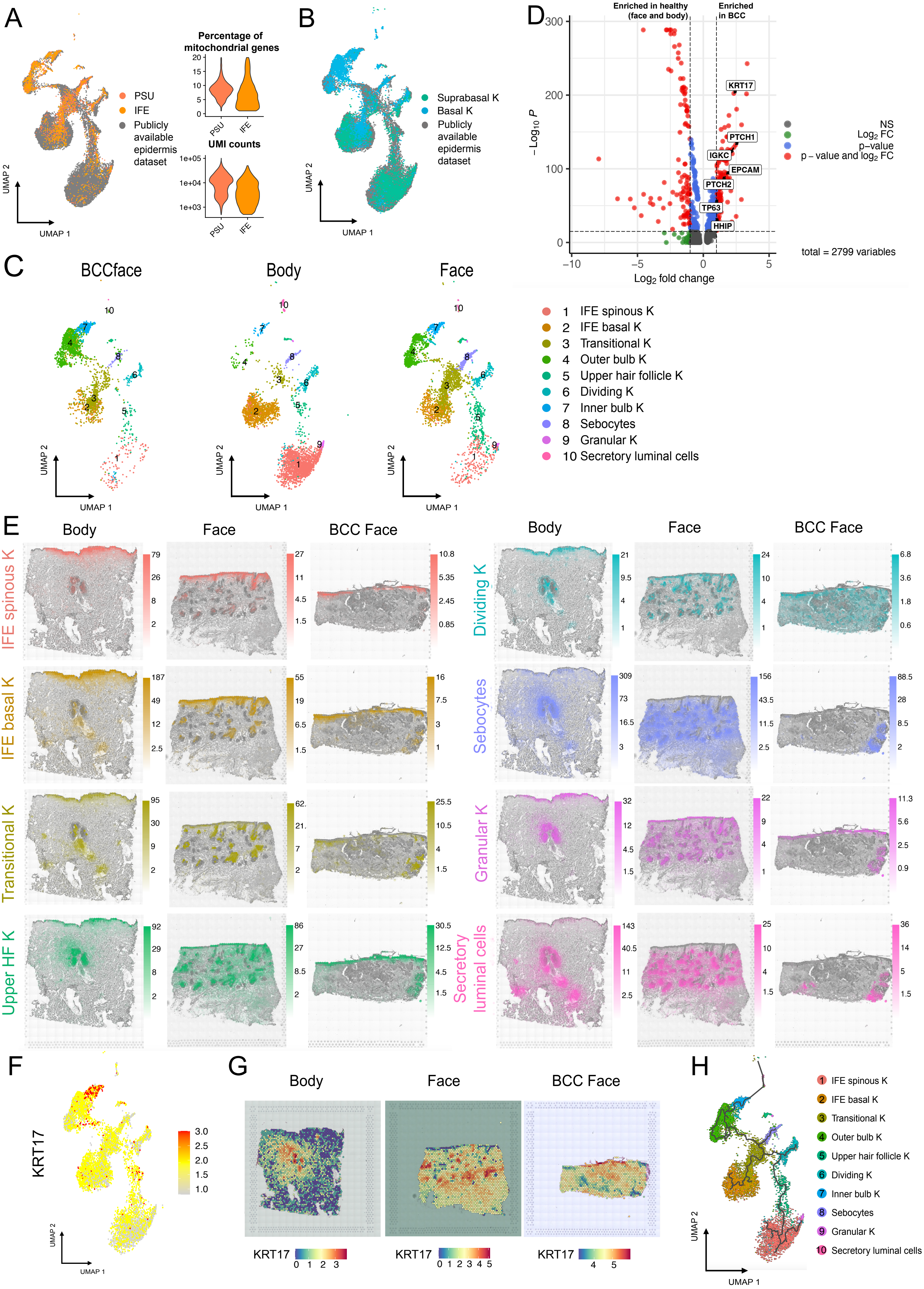

Supplementary Figure 9

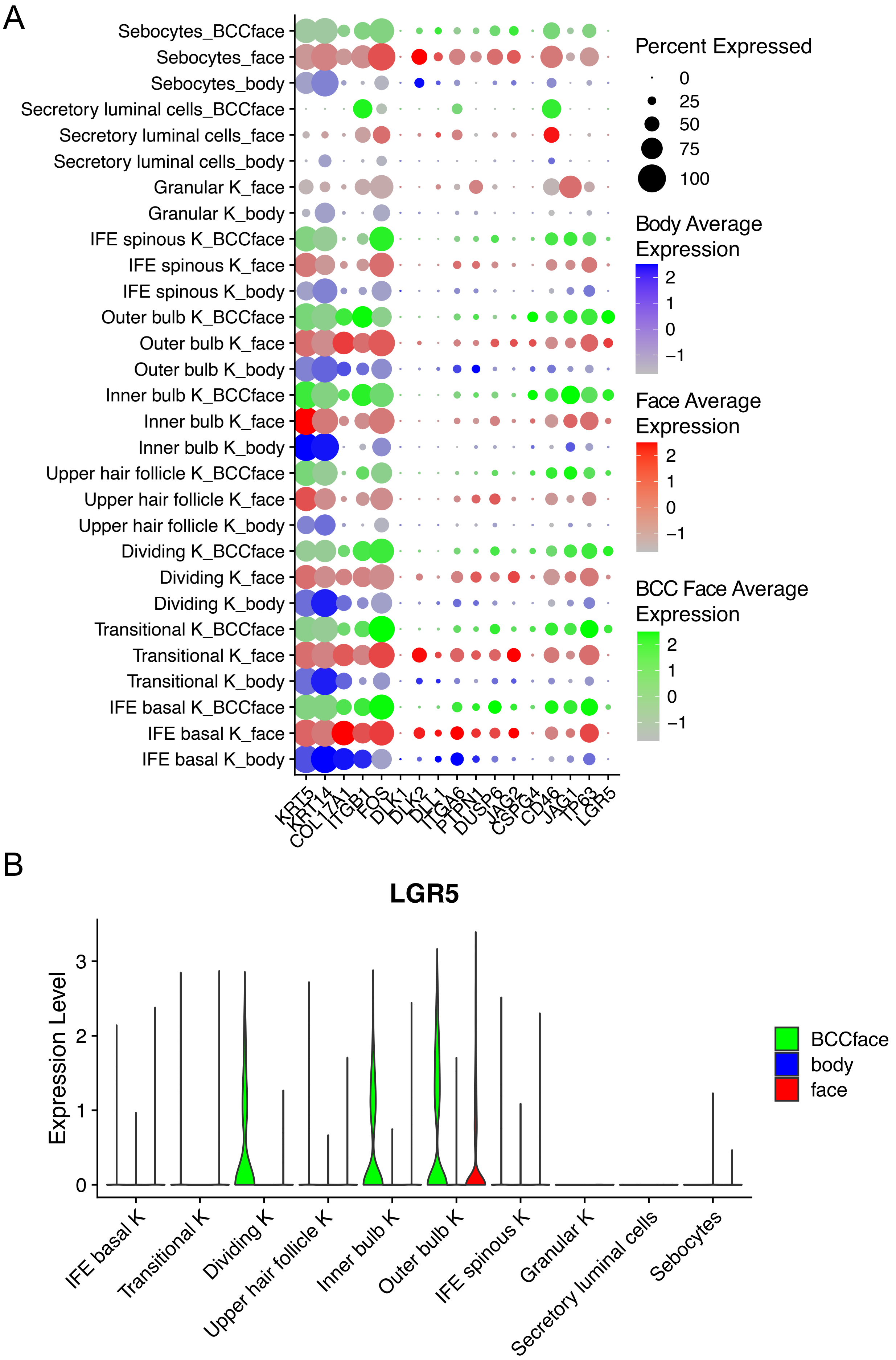

Supplementary Table 1: Top fifty differentially expressed genes within all skin and BCC cell clusters

		p_val	avg_log2FC	pct.1	pct.2	p_val_adj	cluster	gene
1	IL7R	0	1.97390835409899	0.903	0.287	0	Th	IL7R
2	RORA	0	1.78427339536122	0.873	0.318	0	Th	RORA
3	TRAT1	0	1.58858723592029	0.624	0.102	0	Th	TRAT1
4	SARAF	0	1.5805225021889	0.918	0.484	0	Th	SARAF
5	KLRB1	0	1.54937084956506	0.409	0.084	0	Th	KLRB1
6	SPOCK2	0	1.5472782586616	0.804	0.205	0	Th	SPOCK2
7	NR3C1	0	1.43211040116228	0.815	0.437	0	Th	NR3C1
8	ZFP36L2	0	1.42382464647342	0.937	0.694	0	Th	ZFP36L2
9	PTPRC	0	1.37676234586136	0.974	0.506	0	Th	PTPRC
10	CREM	0	1.35835503713398	0.97	0.622	0	Th	CREM
11	ANKRD12	0	1.35602929586347	0.845	0.567	0	Th	ANKRD12
12	EML4	0	1.32925458940398	0.866	0.407	0	Th	EML4
13	EZR	0	1.30160326372498	0.949	0.585	0	Th	EZR
14	TUBA4A	0	1.28612416612016	0.784	0.311	0	Th	TUBA4A
15	LEPROTL1	0	1.26719588332785	0.771	0.27	0	Th	LEPROTL1
16	MBP	0	1.26620303547671	0.701	0.285	0	Th	MBP
17	ARHGDIB	0	1.24990951909301	0.895	0.441	0	Th	ARHGDIB
18	ALOX5AP	0	1.21591901599327	0.637	0.205	0	Th	ALOX5AP
19	CXCR6	0	1.20783289285518	0.429	0.086	0	Th	CXCR6
20	CNOT6L	0	1.18948545440541	0.86	0.376	0	Th	CNOT6L
21	TGFB1	0	1.18929172913255	0.752	0.352	0	Th	TGFB1
22	CD40LG	0	1.17528665431534	0.468	0.066	0	Th	CD40LG
23	CLEC2D	0	1.16005964234168	0.797	0.274	0	Th	CLEC2D
24	CDC42SE2	0	1.15230544954736	0.856	0.388	0	Th	CDC42SE2
25	SAMSN1	0	1.11897963951115	0.871	0.396	0	Th	SAMSN1
26	CCR6	0	1.11451662115856	0.483	0.094	0	Th	CCR6
27	SRGN	0	1.10890894377614	0.995	0.657	0	Th	SRGN
28	LTB	0	1.10108926101575	0.671	0.21	0	Th	LTB
29	FAM107B	0	1.09453665877959	0.828	0.476	0	Th	FAM107B
30	BCL11B	0	1.09336970671836	0.61	0.173	0	Th	BCL11B
31	CLDND1	0	1.08798799028251	0.72	0.362	0	Th	CLDND1
32	CYTIP	0	1.0850147081047	0.891	0.413	0	Th	CYTIP

33	STK4	0	1.07945710041434	0.945	0.512	0	Th	STK4
34	IKZF3	0	1.07820997453739	0.467	0.148	0	Th	IKZF3
35	CD96	0	1.07614100971626	0.538	0.137	0	Th	CD96
36	FAM129A	0	1.07570906144977	0.602	0.241	0	Th	FAM129A
37	CCDC107	0	1.07332571598371	0.639	0.285	0	Th	CCDC107
38	TRBC2	0	1.06755852386419	0.596	0.197	0	Th	TRBC2
39	GLIPR1	0	1.06598926818202	0.592	0.242	0	Th	GLIPR1
40	DUSP4	0	1.05022829785822	0.757	0.353	0	Th	DUSP4
41	CRYBG1	0	1.03896634887751	0.577	0.188	0	Th	CRYBG1
42	CXCR4	0	1.0231639738175	0.886	0.461	0	Th	CXCR4
43	FYB1	0	1.01166016752636	0.648	0.241	0	Th	FYB1
44	CD2	0	1.00881854239204	0.766	0.261	0	Th	CD2
45	STK17B	0	1.00396189992063	0.86	0.444	0	Th	STK17B
46	SELENOK	0	1.00030216000477	0.89	0.586	0	Th	SELENOK
47	PDE4D	0	0.994709088383684	0.507	0.205	0	Th	PDE4D
48	CD6	0	0.990296000597219	0.446	0.094	0	Th	CD6
49	TRBC1	0	0.986057478786573	0.481	0.158	0	Th	TRBC1
50	FNBP1	0	0.980793641356541	0.816	0.455	0	Th	FNBP1
51	CCL5	0	3.59017509070143	0.914	0.087	0	NK	CCL5
52	NKG7	0	3.02762660588989	0.771	0.047	0	NK	NKG7
53	IFNG	0	2.87714324132015	0.509	0.059	0	NK	IFNG
54	GZMK	0	2.68745626632778	0.63	0.021	0	NK	GZMK
55	CCL4	0	2.49775004545892	0.533	0.082	0	NK	CCL4
56	CST7	0	2.24026755222372	0.776	0.19	0	NK	CST7
57	CRTAM	0	2.23948303572465	0.475	0.03	0	NK	CRTAM
58	CCL4L2	0	2.18803866414469	0.267	0.032	0	NK	CCL4L2
59	PIK3R11	0	2.00133126351292	0.741	0.318	0	NK	PIK3R1
60	GZMB	0	1.99096727640418	0.357	0.027	0	NK	GZMB
61	CD8A	0	1.94916236722377	0.537	0.032	0	NK	CD8A
62	GZMA	0	1.83815111737555	0.43	0.039	0	NK	GZMA
63	SH2D1A	0	1.77841148555605	0.559	0.1	0	NK	SH2D1A
64	GZMH	0	1.77120461816818	0.381	0.013	0	NK	GZMH
65	DUSP2	0	1.7024951647228	0.911	0.455	0	NK	DUSP2
66	CLEC2B	0	1.57361356619945	0.803	0.411	0	NK	CLEC2B

67	RUNX31	0	1.49580168739309	0.778	0.364	0	NK	RUNX3
68	TNFRSF9	0	1.46918531159195	0.428	0.121	0	NK	TNFRSF9
69	TUBA4A1	0	1.46557605559193	0.745	0.347	0	NK	TUBA4A
70	PTPRC1	0	1.37930959450765	0.948	0.54	0	NK	PTPRC
71	CD8B	0	1.37807156896911	0.355	0.02	0	NK	CD8B
72	CMC1	0	1.37338277999919	0.309	0.075	0	NK	CMC1
73	AKNA	0	1.36884664412926	0.496	0.143	0	NK	AKNA
74	PPP2R5C1	0	1.34654542176203	0.752	0.384	0	NK	PPP2R5C
75	CEMIP21	0	1.34033583967449	0.653	0.332	0	NK	CEMIP2
76	RNF19A1	0	1.31819418429932	0.75	0.394	0	NK	RNF19A
77	EOMES	0	1.30971832977382	0.313	0.01	0	NK	EOMES
78	CXCR41	0	1.30843943247273	0.872	0.491	0	NK	CXCR4
79	CD3D1	0	1.22489631603925	0.666	0.252	0	NK	CD3D
80	SRSF71	0	1.21733204975847	0.922	0.716	0	NK	SRSF7
81	CLEC2D1	0	1.21653335755195	0.724	0.316	0	NK	CLEC2D
82	GZMM	0	1.21357985454709	0.396	0.065	0	NK	GZMM
83	SYTL31	0	1.19416959039844	0.582	0.224	0	NK	SYTL3
84	CD7	0	1.19115459344965	0.465	0.14	0	NK	CD7
85	TRGC2	0	1.18849855791401	0.291	0.032	0	NK	TRGC2
86	ENC1	0	1.18597310318275	0.287	0.027	0	NK	ENC1
87	PTPN221	0	1.17754958269377	0.51	0.17	0	NK	PTPN22
88	LEPROTL11	0	1.16406975712403	0.666	0.314	0	NK	LEPROTL1
89	CD691	0	1.14837293540131	0.708	0.311	0	NK	CD69
90	HMGB21	0	1.14549833055886	0.625	0.38	0	NK	HMGB2
91	LINC018711	0	1.13951240497189	0.322	0.082	0	NK	LINC01871
92	ITM2A1	0	1.13514820539254	0.539	0.266	0	NK	ITM2A
93	CNOT6L1	0	1.13054520649577	0.764	0.417	0	NK	CNOT6L
94	CD3G1	0	1.12618176280686	0.48	0.158	0	NK	CD3G
95	FYN1	0	1.12399675959275	0.69	0.357	0	NK	FYN
96	METRNL	0	1.09823817273292	0.593	0.36	0	NK	METRNL
97	TNFSF9	0	1.09203417300739	0.289	0.087	0	NK	TNFSF9
98	SAMD3	0	1.0838011557729	0.28	0.02	0	NK	SAMD3
99	RASGRP11	0	1.07601770387019	0.465	0.174	0	NK	RASGRP1
100	LAG3	0	1.06284521885183	0.319	0.039	0	NK	LAG3

101	CFD	0	3.46326653256385	0.972	0.293	0	APOD+ 1	CFD
102	APOD	0	3.12279080852043	0.931	0.181	0	APOD+ 1	APOD
103	APOE	0	3.10536583546416	0.849	0.271	0	APOD+ 1	APOE
104	PTGDS	0	3.04498968972274	0.661	0.101	0	APOD+ 1	PTGDS
105	CXCL12	0	2.88099768784352	0.853	0.131	0	APOD+ 1	CXCL12
106	CCDC80	0	2.8634112955035	0.946	0.177	0	APOD+ 1	CCDC80
107	GSN	0	2.80577361896323	0.926	0.329	0	APOD+ 1	GSN
108	MGP	0	2.76268416334678	0.949	0.256	0	APOD+ 1	MGP
109	CFH	0	2.68339796510441	0.869	0.124	0	APOD+ 1	CFH
110	DCN	0	2.64377587388065	0.99	0.281	0	APOD+ 1	DCN
111	FGF7	0	2.64043942626301	0.738	0.073	0	APOD+ 1	FGF7
112	TNFAIP6	0	2.63282028338304	0.615	0.094	0	APOD+ 1	TNFAIP6
113	CXCL14	0	2.60647605117058	0.925	0.217	0	APOD+ 1	CXCL14
114	MEG3	0	2.52757386846696	0.786	0.114	0	APOD+ 1	MEG3
115	MT2A	0	2.47764503976724	0.911	0.629	0	APOD+ 1	MT2A
116	CXCL1	0	2.47629879179988	0.351	0.09	0	APOD+ 1	CXCL1
117	NNMT	0	2.38902292506913	0.877	0.202	0	APOD+ 1	NNMT
118	MT1M	0	2.37389178670868	0.605	0.134	0	APOD+ 1	MT1M
119	MEDAG	0	2.28470146526534	0.535	0.045	0	APOD+ 1	MEDAG
120	RND3	0	2.21048858636457	0.779	0.174	0	APOD+ 1	RND3
121	PLA2G2A	0	2.1943587446302	0.347	0.029	0	APOD+ 1	PLA2G2A
122	CCL2	0	2.16620066810846	0.746	0.226	0	APOD+ 1	CCL2
123	C1S	0	2.16177044970569	0.813	0.128	0	APOD+ 1	C1S
124	C11orf96	0	2.1539235265234	0.814	0.247	0	APOD+ 1	C11orf96
125	C3	0	2.04620038413225	0.61	0.05	0	APOD+ 1	C3
126	C1R	0	2.03292166777641	0.799	0.135	0	APOD+ 1	C1R
127	MT1X	0	1.99174816120853	0.672	0.257	0	APOD+ 1	MT1X
128	IGFBP7	0	1.99070929746555	0.924	0.311	0	APOD+ 1	IGFBP7
129	FBLN1	0	1.952398474032	0.789	0.118	0	APOD+ 1	FBLN1
130	SERPING1	0	1.92689230208521	0.828	0.175	0	APOD+ 1	SERPING1
131	CXCL2	0	1.88243827636348	0.726	0.278	0	APOD+ 1	CXCL2
132	DPT	0	1.87200397998351	0.63	0.064	0	APOD+ 1	DPT
133	EGR1	0	1.84642794378123	0.727	0.239	0	APOD+ 1	EGR1
134	CYR61	0	1.83495654775602	0.686	0.199	0	APOD+ 1	CYR61

135	CEBPD	0	1.81789582241969	0.881	0.395	0	APOD+ f	CEBPD
136	GEM	0	1.81593185269626	0.788	0.283	0	APOD+ f	GEM
137	SERPINF1	0	1.80873583677775	0.773	0.178	0	APOD+ f	SERPINF1
138	CEBPB	0	1.79509538825895	0.881	0.472	0	APOD+ f	CEBPB
139	UAP1	0	1.77236063841996	0.688	0.188	0	APOD+ f	UAP1
140	HAS2	0	1.73940549158905	0.459	0.048	0	APOD+ f	HAS2
141	CTSL	0	1.73538367613274	0.726	0.25	0	APOD+ f	CTSL
142	MT1A	0	1.72629736907598	0.509	0.106	0	APOD+ f	MT1A
143	TWIST1	0	1.72230607541304	0.613	0.126	0	APOD+ f	TWIST1
144	COL6A2	0	1.68648687809844	0.886	0.233	0	APOD+ f	COL6A2
145	RARRES2	0	1.67591140782214	0.617	0.08	0	APOD+ f	RARRES2
146	IGFBP5	0	1.66694329672807	0.649	0.149	0	APOD+ f	IGFBP5
147	LUM	0	1.6550516481048	0.704	0.135	0	APOD+ f	LUM
148	FST	0	1.6523865827751	0.363	0.045	0	APOD+ f	FST
149	MFAP4	0	1.6375968046332	0.656	0.097	0	APOD+ f	MFAP4
150	EFEMP1	0	1.5998316706513	0.555	0.048	0	APOD+ f	EFEMP1
151	CD692	0	2.0710463587906	0.798	0.311	0	T RM	CD69
152	TNF	0	1.77820005381196	0.434	0.126	0	T RM	TNF
153	DNAJB1	0	1.54334508624975	0.947	0.738	0	T RM	DNAJB1
154	KLF6	0	1.48387861813511	0.902	0.701	0	T RM	KLF6
155	PTGER41	0	1.47027377410847	0.674	0.344	0	T RM	PTGER4
156	BTG2	0	1.41345622048063	0.749	0.502	0	T RM	BTG2
157	CD40LG1	0	1.38351357457581	0.438	0.101	0	T RM	CD40LG
158	PLIN21	0	1.34611437974733	0.549	0.392	0	T RM	PLIN2
159	HSPA81	0	1.32523493342323	0.951	0.846	0	T RM	HSPA8
160	KLRB11	0	1.29962266438229	0.373	0.114	0	T RM	KLRB1
161	HSPA1A1	0	1.27722662285554	0.97	0.864	0	T RM	HSPA1A
162	DDIT4	0	1.27532568812622	0.709	0.502	0	T RM	DDIT4
163	HSPA1B1	0	1.26541133887579	0.915	0.686	0	T RM	HSPA1B
164	CXCR42	0	1.24422050070666	0.808	0.502	0	T RM	CXCR4
165	UBC	0	1.2345458578343	0.981	0.944	0	T RM	UBC
166	CD522	0	1.22295332353711	0.727	0.373	0	T RM	CD52
167	HSP90AA11	0	1.22076009604185	0.987	0.957	0	T RM	HSP90AA1
168	TNFSF14	0	1.21734524096046	0.337	0.088	0	T RM	TNFSF14

169	SLC2A31	0	1.19169241642402	0.844	0.656	0	T RM	SLC2A3
170	DUSP21	0	1.1700625386471	0.781	0.473	0	T RM	DUSP2
171	ANKRD281	0	1.16700806122335	0.525	0.275	0	T RM	ANKRD28
172	IL7R1	0	1.09423722250549	0.783	0.347	0	T RM	IL7R
173	ZC3HAV12	0	1.09326791745485	0.683	0.459	0	T RM	ZC3HAV1
174	LTB1	0	1.0912366215252	0.582	0.255	0	T RM	LTB
175	SRSF72	0	1.07898063414532	0.891	0.722	0	T RM	SRSF7
176	TSPYL21	0	1.07624399703965	0.64	0.452	0	T RM	TSPYL2
177	TXNIP	0	1.07533728235028	0.654	0.443	0	T RM	TXNIP
178	RGS2	0	1.07059304254586	0.679	0.423	0	T RM	RGS2
179	TNFAIP3	0	1.04991184610705	0.73	0.552	0	T RM	TNFAIP3
180	DNAJA11	0	1.04871986091578	0.875	0.778	0	T RM	DNAJA1
181	ICOS2	0	1.0423672032098	0.428	0.171	0	T RM	ICOS
182	PRDM11	0	1.03551572541977	0.569	0.317	0	T RM	PRDM1
183	DUSP42	0	1.03315186049289	0.612	0.398	0	T RM	DUSP4
184	DUSP51	0	1.03168877335626	0.538	0.333	0	T RM	DUSP5
185	TAGAP1	0	1.02974357574827	0.476	0.21	0	T RM	TAGAP
186	RGCC2	0	1.02313991345021	0.621	0.426	0	T RM	RGCC
187	RGS11	0	1.01814641549303	0.642	0.393	0	T RM	RGS1
188	UBE2S1	0	1.00062622078582	0.616	0.433	0	T RM	UBE2S
189	CITED2	0	0.998035299736573	0.419	0.23	0	T RM	CITED2
190	CD22	0	0.996463342805441	0.702	0.308	0	T RM	CD2
191	DOK21	0	0.957216080729092	0.513	0.249	0	T RM	DOK2
192	CGAS	0	0.955749396925654	0.378	0.161	0	T RM	CGAS
193	PPP1R22	0	0.943834569661462	0.665	0.47	0	T RM	PPP1R2
194	AC058791.1	0	0.932216841281692	0.597	0.358	0	T RM	AC058791.1
195	ITK2	0	0.929110978949825	0.453	0.177	0	T RM	ITK
196	CACYBP1	0	0.90400349421489	0.695	0.519	0	T RM	CACYBP
197	RHOH2	0	0.902775174284441	0.568	0.286	0	T RM	RHOH
198	CLK1	0	0.889080522980747	0.767	0.583	0	T RM	CLK1
199	DEDD21	0	0.880004965558498	0.443	0.224	0	T RM	DEDD2
200	BATF1	0	0.874521918143521	0.439	0.19	0	T RM	BATF
201	CTLA4	0	2.3928394240666	0.722	0.071	0	T reg	CTLA4
202	TIGIT1	0	2.07956020337325	0.647	0.091	0	T reg	TIGIT

203	IL323	0	2.02346148788152	0.837	0.391	0	T reg	IL32
204	CARD16	0	1.99891389757546	0.657	0.192	0	T reg	CARD16
205	PMAIP1	0	1.96834648975399	0.706	0.269	0	T reg	PMAIP1
206	TNFRSF41	0	1.93438227394699	0.546	0.125	0	T reg	TNFRSF4
207	TNFRSF182	0	1.91572855626894	0.716	0.218	0	T reg	TNFRSF18
208	IKZF2	0	1.86797227960874	0.517	0.053	0	T reg	IKZF2
209	ICOS3	0	1.82047511088914	0.66	0.153	0	T reg	ICOS
210	TNFRSF91	0	1.80288891444374	0.579	0.114	0	T reg	TNFRSF9
211	TBC1D4	0	1.75717594124963	0.563	0.076	0	T reg	TBC1D4
212	BATF2	0	1.67781631742143	0.649	0.173	0	T reg	BATF
213	IL2RA	0	1.63115268532808	0.421	0.053	0	T reg	IL2RA
214	DUSP43	0	1.53226125594729	0.818	0.382	0	T reg	DUSP4
215	TRBC23	0	1.53052230053567	0.65	0.226	0	T reg	TRBC2
216	CLEC2D2	0	1.51998555449234	0.789	0.318	0	T reg	CLEC2D
217	TRAC3	0	1.51779442373243	0.66	0.203	0	T reg	TRAC
218	TRBC13	0	1.50853224408966	0.517	0.181	0	T reg	TRBC1
219	LTB2	0	1.49404759131245	0.628	0.252	0	T reg	LTB
220	CD23	0	1.49109846091097	0.785	0.301	0	T reg	CD2
221	GBP5	0	1.48736133707176	0.44	0.063	0	T reg	GBP5
222	LAYN	0	1.44611661955576	0.374	0.035	0	T reg	LAYN
223	CD71	0	1.42648070059318	0.554	0.138	0	T reg	CD7
224	CD27	0	1.37472121948881	0.435	0.052	0	T reg	CD27
225	NSD32	0	1.34010356221502	0.766	0.425	0	T reg	NSD3
226	RGS12	0	1.31585882694688	0.738	0.386	0	T reg	RGS1
227	UGP21	0	1.31031766307353	0.608	0.361	0	T reg	UGP2
228	CD3D3	0	1.29954821112546	0.703	0.255	0	T reg	CD3D
229	RTKN2	0	1.29437920267859	0.313	0.052	0	T reg	RTKN2
230	CORO1B	0	1.27519524425267	0.446	0.137	0	T reg	CORO1B
231	TTN	0	1.26795606385453	0.403	0.073	0	T reg	TTN
232	GK	0	1.25616968255922	0.462	0.14	0	T reg	GK
233	CYTIP3	0	1.25455454206102	0.843	0.456	0	T reg	CYTIP
234	SOD13	0	1.25433167648544	0.856	0.654	0	T reg	SOD1
235	RAB11FIP12	0	1.25398405953189	0.619	0.272	0	T reg	RAB11FIP1
236	LINC01943	0	1.25171748032319	0.365	0.04	0	T reg	LINC01943

237	ZC3H12D1	0	1.19808798373732	0.479	0.121	0	T reg	ZC3H12D
238	SPOCK23	0	1.19755675945918	0.702	0.262	0	T reg	SPOCK2
239	MAGEH1	0	1.19667996833465	0.291	0.067	0	T reg	MAGEH1
240	CD3E3	0	1.1948590885405	0.642	0.216	0	T reg	CD3E
241	FOXP3	0	1.19158109225392	0.302	0.004	0	T reg	FOXP3
242	CD283	0	1.17694477628922	0.567	0.175	0	T reg	CD28
243	STAM1	0	1.17460456059009	0.504	0.155	0	T reg	STAM
244	HTATIP2	0	1.15661943664758	0.374	0.093	0	T reg	HTATIP2
245	AC133644.2	0	1.14706930809551	0.254	0.016	0	T reg	AC133644.2
246	SMAP22	0	1.14534865479664	0.673	0.344	0	T reg	SMAP2
247	CTSC	0	1.14035344448927	0.521	0.268	0	T reg	CTSC
248	ZNF292	0	1.13809748033052	0.538	0.25	0	T reg	ZNF292
249	HLA-A2	0	1.13347999641952	0.967	0.866	0	T reg	HLA-A
250	PPP1R23	0	1.12122350424523	0.738	0.464	0	T reg	PPP1R2
251	HMOX1	0	3.96446107524358	0.708	0.155	0	Macro1_	HMOX1
252	RNASE1	0	3.75881997188123	0.631	0.06	0	Macro1_	RNASE1
253	C1QA	0	3.36358367497106	0.777	0.046	0	Macro1_	C1QA
254	CXCL8	0	3.26651970326482	0.793	0.252	0	Macro1_	CXCL8
255	CCL3	0	3.04676317704746	0.447	0.075	0	Macro1_	CCL3
256	CTSL1	0	3.01304703247624	0.823	0.246	0	Macro1_	CTSL
257	CTSB	0	2.99278054360039	0.908	0.226	0	Macro1_	CTSB
258	MMP9	0	2.94059416501109	0.664	0.079	0	Macro1_	MMP9
259	CXCL31	0	2.90882739275502	0.758	0.19	0	Macro1_	CXCL3
260	FCGR2A	0	2.70665709595213	0.838	0.074	0	Macro1_	FCGR2A
261	FTL	0	2.66915601039518	0.998	0.96	0	Macro1_	FTL
262	C5AR1	0	2.66187194663018	0.802	0.052	0	Macro1_	C5AR1
263	EREG	0	2.5865199648891	0.379	0.073	0	Macro1_	EREG
264	AIF1	0	2.58027743683602	0.85	0.105	0	Macro1_	AIF1
265	SDS	0	2.54915378841449	0.52	0.026	0	Macro1_	SDS
266	HLA-DRA	0	2.54411560399545	0.983	0.468	0	Macro1_	HLA-DRA
267	C1QB	0	2.53567501250212	0.633	0.028	0	Macro1_	C1QB
268	SELENOP1	0	2.52250019139752	0.576	0.161	0	Macro1_	SELENOP
269	C1QC	0	2.47184377021033	0.652	0.024	0	Macro1_	C1QC
270	CTSZ	0	2.44947922401187	0.909	0.285	0	Macro1_	CTSZ

271	TYROBP	0	2.43487866191131	0.915	0.136	0	Macro1_	TYROBP
272	FGL21	0	2.39951190585251	0.793	0.21	0	Macro1_	FGL2
273	F13A1	0	2.39757718476139	0.581	0.044	0	Macro1_	F13A1
274	FCER1G	0	2.37328110225703	0.886	0.118	0	Macro1_	FCER1G
275	CD74	0	2.36779431500958	0.985	0.567	0	Macro1_	CD74
276	LGMN	0	2.34918098939848	0.637	0.108	0	Macro1_	LGMN
277	IL1B	0	2.30794045647015	0.472	0.114	0	Macro1_	IL1B
278	HLA-DPA1	0	2.30137692529804	0.955	0.377	0	Macro1_	HLA-DPA1
279	MS4A7	0	2.28285670679374	0.717	0.043	0	Macro1_	MS4A7
280	CXCL21	0	2.27435691252748	0.829	0.273	0	Macro1_	CXCL2
281	FNIP2	0	2.25427069215203	0.722	0.124	0	Macro1_	FNIP2
282	HLA-DRB1	0	2.24779921162008	0.958	0.427	0	Macro1_	HLA-DRB1
283	MRC1	0	2.24407989581235	0.648	0.036	0	Macro1_	MRC1
284	MS4A6A	0	2.23103073442602	0.773	0.068	0	Macro1_	MS4A6A
285	PLEK	0	2.20085211603927	0.787	0.126	0	Macro1_	PLEK
286	CD68	0	2.20002560714069	0.808	0.087	0	Macro1_	CD68
287	LYZ	0	2.16251407258767	0.759	0.16	0	Macro1_	LYZ
288	PSAP1	0	2.14464225513254	0.918	0.415	0	Macro1_	PSAP
289	IL1RN	0	2.1397725889558	0.578	0.071	0	Macro1_	IL1RN
290	INSIG1	0	2.13111250133455	0.845	0.358	0	Macro1_	INSIG1
291	TGFBI	0	2.08504330081769	0.743	0.12	0	Macro1_	TGFBI
292	PLAUR1	0	2.07941073989758	0.874	0.293	0	Macro1_	PLAUR
293	HLA-DQA1	0	2.05824577659951	0.879	0.226	0	Macro1_	HLA-DQA1
294	FOLR2	0	2.05250482885	0.524	0.013	0	Macro1_	FOLR2
295	HLA-DPB1	0	2.01619850131759	0.944	0.387	0	Macro1_	HLA-DPB1
296	GPR1831	0	1.9824690340457	0.902	0.429	0	Macro1_	GPR183
297	SAT1	0	1.98216215019905	0.986	0.836	0	Macro1_	SAT1
298	HLA-DRB5	0	1.96281394520703	0.676	0.207	0	Macro1_	HLA-DRB5
299	MMP19	0	1.93112504840608	0.472	0.046	0	Macro1_	MMP19
300	C15orf48	0	1.91246177892027	0.559	0.116	0	Macro1_	C15orf48
301	HLA-DRA1	0	3.51381982801513	0.997	0.474	0	DC1	HLA-DRA
302	G0S21	0	3.41129125961984	0.719	0.182	0	DC1	G0S2
303	HLA-DPB11	0	3.40308286979823	0.989	0.392	0	DC1	HLA-DPB1
304	HLA-DQA11	0	3.28057867007514	0.976	0.229	0	DC1	HLA-DQA1

305	HLA-DPA11	0	3.27975830821445	0.988	0.383	0	DC1	HLA-DPA1
306	HLA-DRB11	0	3.26634776166907	0.99	0.432	0	DC1	HLA-DRB1
307	LYZ1	0	3.08558663191892	0.91	0.16	0	DC1	LYZ
308	C15orf481	0	2.93095038912384	0.746	0.112	0	DC1	C15orf48
309	CD741	0	2.92875963713173	0.996	0.572	0	DC1	CD74
310	HLA-DQB11	0	2.90277589876416	0.964	0.29	0	DC1	HLA-DQB1
311	HLA-DRB51	0	2.7682291409221	0.785	0.208	0	DC1	HLA-DRB5
312	FCER1A1	0	2.72234743355066	0.73	0.035	0	DC1	FCER1A
313	CST32	0	2.44012314808838	0.976	0.496	0	DC1	CST3
314	CD831	0	2.35116337827933	0.879	0.236	0	DC1	CD83
315	CXCL81	0	2.23835400230716	0.7	0.265	0	DC1	CXCL8
316	IL1R21	0	2.21537846770206	0.681	0.06	0	DC1	IL1R2
317	CTSZ1	0	2.21411521738248	0.885	0.294	0	DC1	CTSZ
318	TYROBP1	0	2.15272905738606	0.922	0.146	0	DC1	TYROBP
319	INSIG11	0	2.14883606682868	0.846	0.365	0	DC1	INSIG1
320	LST11	0	2.08085914351176	0.818	0.11	0	DC1	LST1
321	HLA-DMA1	0	2.0583672334134	0.867	0.158	0	DC1	HLA-DMA
322	FCER1G1	0	2.04726893008108	0.871	0.13	0	DC1	FCER1G
323	IL1B1	0	2.03417141106605	0.624	0.111	0	DC1	IL1B
324	HLA-DQA21	0	2.02976041693227	0.581	0.074	0	DC1	HLA-DQA2
325	AREG1	0	2.02123494764863	0.488	0.164	0	DC1	AREG
326	CLEC10A1	0	2.01712027341704	0.681	0.036	0	DC1	CLEC10A
327	GPR1832	0	2.01507634959092	0.912	0.435	0	DC1	GPR183
328	AIF11	0	1.97846441184739	0.826	0.116	0	DC1	AIF1
329	SAT11	0	1.96248218366643	0.981	0.838	0	DC1	SAT1
330	GPAT31	0	1.95919703813739	0.637	0.102	0	DC1	GPAT3
331	LGALS2	0	1.89683889448822	0.633	0.038	0	DC1	LGALS2
332	CCR7	0	1.88968511476788	0.468	0.087	0	DC1	CCR7
333	CPVL1	0	1.87193416746427	0.666	0.065	0	DC1	CPVL
334	PPIF1	0	1.84764059181754	0.678	0.182	0	DC1	PPIF
335	PLAUR2	0	1.84006302303698	0.875	0.301	0	DC1	PLAUR
336	PLEK1	0	1.81736961179159	0.757	0.136	0	DC1	PLEK
337	SERPINB11	0	1.71706716219337	0.622	0.226	0	DC1	SERPINB1
338	FABP51	0	1.69771915002549	0.653	0.281	0	DC1	FABP5

339	AC020656.1	0	1.66509386918112	0.464	0.064	0	DC1	AC020656.1
340	SERPINB91	0	1.64386913162982	0.791	0.369	0	DC1	SERPINB9
341	CD1C	0	1.63575946851879	0.52	0.015	0	DC1	CD1C
342	CA2	0	1.62471559245509	0.419	0.054	0	DC1	CA2
343	BASP11	0	1.60615207253928	0.751	0.2	0	DC1	BASP1
344	CSF2RA1	0	1.55622573558726	0.627	0.054	0	DC1	CSF2RA
345	RALA1	0	1.5400859513926	0.719	0.245	0	DC1	RALA
346	FTH12	0	1.52717815661834	0.997	0.983	0	DC1	FTH1
347	MS4A6A1	0	1.52440832354331	0.631	0.085	0	DC1	MS4A6A
348	HLA-DQB2	0	1.52188486249954	0.42	0.025	0	DC1	HLA-DQB2
349	SGK11	0	1.51016562783498	0.696	0.271	0	DC1	SGK1
350	GPR157	0	1.50174482615567	0.465	0.04	0	DC1	GPR157
351	SFRP21	0	4.11726694515631	0.837	0.087	0	SFRP2+	SFRP2
352	DCN1	0	3.87451889153788	0.991	0.295	0	SFRP2+	DCN
353	FBLN11	0	3.68021131521306	0.869	0.127	0	SFRP2+	FBLN1
354	CXCL141	0	3.66493446641538	0.927	0.231	0	SFRP2+	CXCL14
355	CFD1	0	3.38214841661242	0.935	0.309	0	SFRP2+	CFD
356	MMP21	0	3.17075367651847	0.861	0.118	0	SFRP2+	MMP2
357	COL1A21	0	3.13135321143601	0.941	0.253	0	SFRP2+	COL1A2
358	GSN1	0	3.01042203762743	0.929	0.34	0	SFRP2+	GSN
359	PLAC91	0	2.93858030121234	0.859	0.16	0	SFRP2+	PLAC9
360	LUM1	0	2.86246337694966	0.826	0.14	0	SFRP2+	LUM
361	COL1A1	0	2.82220812319606	0.863	0.243	0	SFRP2+	COL1A1
362	COL3A11	0	2.77459195300157	0.775	0.197	0	SFRP2+	COL3A1
363	WISP21	0	2.7252916775885	0.571	0.041	0	SFRP2+	WISP2
364	IGFBP61	0	2.70470735350393	0.572	0.081	0	SFRP2+	IGFBP6
365	CTSK1	0	2.66482267108736	0.777	0.109	0	SFRP2+	CTSK
366	MFAP41	0	2.6633590530971	0.742	0.104	0	SFRP2+	MFAP4
367	COL6A21	0	2.65883675538538	0.944	0.243	0	SFRP2+	COL6A2
368	SLPI	0	2.58951634641883	0.333	0.029	0	SFRP2+	SLPI
369	SERPINF11	0	2.57643442723845	0.822	0.188	0	SFRP2+	SERPINF1
370	SEPP1	0	2.43285429633306	0.573	0.052	0	SFRP2+	SEPP1
371	IGFBP51	0	2.39261953248687	0.701	0.156	0	SFRP2+	IGFBP5
372	SELM	0	2.38098193243538	0.574	0.053	0	SFRP2+	SELM

373	C1R1	0	2.36433881713363	0.749	0.151	0	SFRP2+	C1R
374	CD9	0	2.35484443510765	0.803	0.312	0	SFRP2+	CD9
375	PPAP2B	0	2.33823378160485	0.484	0.026	0	SFRP2+	PPAP2B
376	CCDC801	0	2.3230100045433	0.851	0.197	0	SFRP2+	CCDC80
377	PI161	0	2.31887665140152	0.453	0.051	0	SFRP2+	PI16
378	C1S1	0	2.30705527211699	0.799	0.142	0	SFRP2+	C1S
379	COL6A11	0	2.28952833017695	0.816	0.21	0	SFRP2+	COL6A1
380	CTHRC1	0	2.28815094861611	0.518	0.053	0	SFRP2+	CTHRC1
381	FBLN21	0	2.27014448740534	0.667	0.079	0	SFRP2+	FBLN2
382	SOD31	0	2.23652392152467	0.825	0.171	0	SFRP2+	SOD3
383	SPARC	0	2.22823616360622	0.802	0.249	0	SFRP2+	SPARC
384	TIMP21	0	2.19682638682034	0.801	0.263	0	SFRP2+	TIMP2
385	FSTL11	0	2.1767259584359	0.737	0.149	0	SFRP2+	FSTL1
386	MFAP5	0	2.1709957894626	0.438	0.048	0	SFRP2+	MFAP5
387	PTGDS1	0	2.15063827817237	0.443	0.123	0	SFRP2+	PTGDS
388	CFH1	0	2.13653299582202	0.718	0.146	0	SFRP2+	CFH
389	THBS21	0	2.09137424859864	0.61	0.069	0	SFRP2+	THBS2
390	CYBRD11	0	2.08677879028965	0.647	0.103	0	SFRP2+	CYBRD1
391	SERPING11	0	2.01630515606189	0.774	0.191	0	SFRP2+	SERPING1
392	S100A62	0	1.98086404134164	0.996	0.877	0	SFRP2+	S100A6
393	LEPR	0	1.97719607538867	0.407	0.033	0	SFRP2+	LEPR
394	GNB2L1	0	1.96845979622325	0.634	0.101	0	SFRP2+	GNB2L1
395	TPPP3	0	1.94619348548657	0.448	0.062	0	SFRP2+	TPPP3
396	TNFAIP61	0	1.94364056020819	0.533	0.108	0	SFRP2+	TNFAIP6
397	NBL11	0	1.91066220347745	0.621	0.157	0	SFRP2+	NBL1
398	DPT1	0	1.90841968752234	0.548	0.08	0	SFRP2+	DPT
399	MGP1	0	1.87844611315908	0.81	0.277	0	SFRP2+	MGP
400	PPIC	0	1.84598097495565	0.506	0.056	0	SFRP2+	PPIC
401	TAGLN	0	4.43656894906895	0.972	0.117	0	TAGLN+	TAGLN
402	ACTA2	0	4.27077942773467	0.882	0.071	0	TAGLN+	ACTA2
403	TPM2	0	3.96088452617759	0.954	0.127	0	TAGLN+	TPM2
404	MYL9	0	3.84280185093923	0.945	0.131	0	TAGLN+	MYL9
405	C11orf961	0	3.41310348685049	0.945	0.255	0	TAGLN+	C11orf96
406	ADIRF2	0	3.30958282820359	0.978	0.257	0	TAGLN+	ADIRF

407	MYH11	0	3.2107971518011	0.819	0.023	0	TAGLN+	MYH11
408	SPARCL11	0	3.01037772752229	0.937	0.172	0	TAGLN+	SPARCL1
409	ADAMTS11	0	2.90404680717784	0.844	0.162	0	TAGLN+	ADAMTS1
410	RGS5	0	2.8709683337411	0.62	0.059	0	TAGLN+	RGS5
411	MT1A1	0	2.79410799353959	0.615	0.111	0	TAGLN+	MT1A
412	CRISPLD22	0	2.72986179918426	0.788	0.131	0	TAGLN+	CRISPLD2
413	IL61	0	2.67107573078146	0.513	0.12	0	TAGLN+	IL6
414	DSTN2	0	2.6440177061596	0.952	0.448	0	TAGLN+	DSTN
415	AVPR1A	0	2.57011885056351	0.573	0.024	0	TAGLN+	AVPR1A
416	MCAM	0	2.54978815878258	0.848	0.113	0	TAGLN+	MCAM
417	CCL22	0	2.49887419981743	0.741	0.24	0	TAGLN+	CCL2
418	RERGL	0	2.48586858421709	0.481	0.003	0	TAGLN+	RERGL
419	IGFBP72	0	2.47149368373905	0.982	0.324	0	TAGLN+	IGFBP7
420	CALD12	0	2.45520949523911	0.969	0.285	0	TAGLN+	CALD1
421	TPM11	0	2.45245259244734	0.856	0.172	0	TAGLN+	TPM1
422	CRYAB2	0	2.42487479238731	0.788	0.22	0	TAGLN+	CRYAB
423	CYCS	0	2.25642756330058	0.788	0.594	0	TAGLN+	CYCS
424	ADRA2A	0	2.17740446348908	0.552	0.034	0	TAGLN+	ADRA2A
425	NET1	0	2.16970819875838	0.681	0.083	0	TAGLN+	NET1
426	ID41	0	2.13770416796733	0.71	0.118	0	TAGLN+	ID4
427	TINAGL1	0	2.08063578092805	0.715	0.039	0	TAGLN+	TINAGL1
428	GJA4	0	2.06119112418312	0.513	0.033	0	TAGLN+	GJA4
429	NDUFA4L2	0	2.05243650855901	0.594	0.047	0	TAGLN+	NDUFA4L2
430	CPE2	0	2.04519835836905	0.824	0.119	0	TAGLN+	CPE
431	ADAMTS41	0	2.02770479605289	0.545	0.117	0	TAGLN+	ADAMTS4
432	PDK41	0	1.98313163642168	0.441	0.11	0	TAGLN+	PDK4
433	KCNE41	0	1.98009430904981	0.507	0.064	0	TAGLN+	KCNE4
434	CEBPD2	0	1.9548249401102	0.879	0.407	0	TAGLN+	CEBPD
435	MFGE81	0	1.95244141848278	0.772	0.124	0	TAGLN+	MFGE8
436	SORBS2	0	1.95121661449067	0.519	0.014	0	TAGLN+	SORBS2
437	MYLK	0	1.9388774372074	0.732	0.062	0	TAGLN+	MYLK
438	LBH3	0	1.90537249873344	0.805	0.278	0	TAGLN+	LBH
439	BCAM	0	1.89574242926351	0.706	0.062	0	TAGLN+	BCAM
440	EPAS1	0	1.89217531913076	0.771	0.15	0	TAGLN+	EPAS1

441	NOTCH3	0	1.88204431651817	0.683	0.05	0	TAGLN+	NOTCH3
442	CSRP1	0	1.86864918065811	0.604	0.105	0	TAGLN+	CSRP1
443	RASD12	0	1.84216723652302	0.522	0.152	0	TAGLN+	RASD1
444	GADD45B3	0	1.83822061866049	0.916	0.591	0	TAGLN+	GADD45B
445	ZFHX31	0	1.82257485736552	0.694	0.117	0	TAGLN+	ZFHX3
446	PDGFA	0	1.80758788598461	0.656	0.089	0	TAGLN+	PDGFA
447	NR2F21	0	1.78736905437284	0.691	0.106	0	TAGLN+	NR2F2
448	RHOB2	0	1.78609169265661	0.697	0.213	0	TAGLN+	RHOB
449	RGS162	0	1.77885432480735	0.698	0.213	0	TAGLN+	RGS16
450	ATF32	0	1.76955074282661	0.783	0.34	0	TAGLN+	ATF3
451	COL1A11	0	4.4436121906627	0.935	0.251	0	POSTN+	COL1A1
452	COL3A12	0	3.89630155252647	0.899	0.203	0	POSTN+	COL3A1
453	COL1A22	0	3.72659731013533	0.961	0.264	0	POSTN+	COL1A2
454	POSTN	0	3.41060282189462	0.657	0.054	0	POSTN+	POSTN
455	COL6A12	0	2.89247808837419	0.919	0.217	0	POSTN+	COL6A1
456	SPARC2	0	2.78908074104753	0.841	0.257	0	POSTN+	SPARC
457	COL6A22	0	2.63729085630616	0.939	0.255	0	POSTN+	COL6A2
458	COL6A32	0	2.59406613067044	0.786	0.132	0	POSTN+	COL6A3
459	LUM2	0	2.46111912373621	0.826	0.151	0	POSTN+	LUM
460	MEG32	0	2.38104670135235	0.823	0.137	0	POSTN+	MEG3
461	COL5A22	0	2.19164105069816	0.642	0.08	0	POSTN+	COL5A2
462	CTGF3	0	2.12228523036517	0.615	0.153	0	POSTN+	CTGF
463	HTRA12	0	2.10053633943176	0.682	0.12	0	POSTN+	HTRA1
464	ASPN	0	2.09345001456348	0.499	0.031	0	POSTN+	ASPN
465	MFAP51	0	2.0659487727879	0.565	0.051	0	POSTN+	MFAP5
466	COL11A1	0	1.87253552087119	0.396	0.015	0	POSTN+	COL11A1
467	DIO21	0	1.87074087018723	0.505	0.071	0	POSTN+	DIO2
468	COL5A11	0	1.84865153679926	0.479	0.055	0	POSTN+	COL5A1
469	CCDC802	0	1.84425795859932	0.817	0.21	0	POSTN+	CCDC80
470	TWIST12	0	1.828934266552	0.691	0.142	0	POSTN+	TWIST1
471	VCAN2	0	1.79511628662708	0.725	0.146	0	POSTN+	VCAN
472	MMP22	0	1.78799344649128	0.693	0.137	0	POSTN+	MMP2
473	GPC31	0	1.78672431142997	0.443	0.042	0	POSTN+	GPC3
474	CYR613	0	1.78572945125332	0.698	0.216	0	POSTN+	CYR61

475	COL12A13	0	1.77412945957082	0.575	0.086	0	POSTN+	COL12A1
476	FN12	0	1.76368693555006	0.604	0.125	0	POSTN+	FN1
477	THY12	0	1.73645139371886	0.609	0.094	0	POSTN+	THY1
478	RND32	0	1.68839350716998	0.703	0.199	0	POSTN+	RND3
479	FSTL12	0	1.65486798123106	0.715	0.159	0	POSTN+	FSTL1
480	PRRX13	0	1.59425186092567	0.764	0.169	0	POSTN+	PRRX1
481	CALD13	0	1.57147305403106	0.913	0.295	0	POSTN+	CALD1
482	CDH111	0	1.5556450211262	0.556	0.049	0	POSTN+	CDH11
483	MGP2	0	1.55001335568346	0.764	0.287	0	POSTN+	MGP
484	CTHRC11	0	1.54149139959599	0.296	0.068	0	POSTN+	CTHRC1
485	DCN2	0	1.53261374451028	0.961	0.308	0	POSTN+	DCN
486	MXRA5	0	1.52325289891286	0.483	0.04	0	POSTN+	MXRA5
487	INHBA	0	1.51420946916707	0.449	0.079	0	POSTN+	INHBA
488	F2R	0	1.50838631378392	0.547	0.076	0	POSTN+	F2R
489	ID33	0	1.50476754045021	0.715	0.327	0	POSTN+	ID3
490	FBN12	0	1.48967898765698	0.548	0.092	0	POSTN+	FBN1
491	MFAP42	0	1.48113769115516	0.608	0.119	0	POSTN+	MFAP4
492	MXRA82	0	1.48080121468522	0.591	0.08	0	POSTN+	MXRA8
493	PRSS23	0	1.47553526159748	0.459	0.053	0	POSTN+	PRSS23
494	PCOLCE3	0	1.46793869525242	0.623	0.144	0	POSTN+	PCOLCE
495	TCF43	0	1.41837997465464	0.75	0.249	0	POSTN+	TCF4
496	CTSK2	0	1.41088420925052	0.64	0.125	0	POSTN+	CTSK
497	CAVIN12	0	1.4028743965305	0.625	0.167	0	POSTN+	CAVIN1
498	HES13	0	1.39241966203652	0.601	0.235	0	POSTN+	HES1
499	OGN1	0	1.38136325360001	0.379	0.047	0	POSTN+	OGN
500	NNMT2	0	1.37384783391842	0.704	0.233	0	POSTN+	NNMT
501	RGS51	0	3.95860317842631	0.824	0.053	0	RGS5+ p	RGS5
502	RGS163	0	3.2556360039452	0.802	0.211	0	RGS5+ p	RGS16
503	ID43	0	3.1135443021037	0.785	0.118	0	RGS5+ p	ID4
504	TCIM1	0	2.86470296126541	0.538	0.052	0	RGS5+ p	TCIM
505	NR2F22	0	2.84857712681214	0.825	0.103	0	RGS5+ p	NR2F2
506	CCL19	0	2.81686920271023	0.329	0.051	0	RGS5+ p	CCL19
507	STEAP4	0	2.60326150374859	0.627	0.044	0	RGS5+ p	STEAP4
508	CCL23	0	2.57671187929255	0.678	0.244	0	RGS5+ p	CCL2

509	CALD14	0	2.5013871171113	0.955	0.288	0	RGS5+ p	CALD1
510	KCNE42	0	2.49273745810784	0.584	0.062	0	RGS5+ p	KCNE4
511	CRISPLD23	0	2.43199120249369	0.73	0.136	0	RGS5+ p	CRISPLD2
512	PDGFRB4	0	2.4205324896934	0.795	0.119	0	RGS5+ p	PDGFRB
513	NDUFA4L21	0	2.32924046547758	0.554	0.051	0	RGS5+ p	NDUFA4L2
514	ADAMTS42	0	2.22961326059577	0.577	0.117	0	RGS5+ p	ADAMTS4
515	ANGPT21	0	2.19836760832914	0.65	0.049	0	RGS5+ p	ANGPT2
516	IGFBP73	0	2.17500327954868	0.924	0.329	0	RGS5+ p	IGFBP7
517	ADAMTS13	0	2.16961319769152	0.713	0.17	0	RGS5+ p	ADAMTS1
518	GJA41	0	2.14256227527685	0.493	0.035	0	RGS5+ p	GJA4
519	C11orf963	0	2.10471080885966	0.856	0.261	0	RGS5+ p	C11orf96
520	PRRX14	0	2.095435153084	0.808	0.163	0	RGS5+ p	PRRX1
521	COL18A12	0	2.08390155484381	0.668	0.115	0	RGS5+ p	COL18A1
522	SYNPO21	0	2.08272313651643	0.54	0.038	0	RGS5+ p	SYNPO2
523	SPARCL12	0	2.07109212658123	0.776	0.182	0	RGS5+ p	SPARCL1
524	PDGFA1	0	2.04026791544306	0.612	0.093	0	RGS5+ p	PDGFA
525	COL4A13	0	2.0186908162717	0.602	0.097	0	RGS5+ p	COL4A1
526	TAGLN1	0	2.00742653197557	0.727	0.13	0	RGS5+ p	TAGLN
527	CPE4	0	1.97409019244078	0.706	0.126	0	RGS5+ p	CPE
528	LHFPL63	0	1.93375411151281	0.613	0.099	0	RGS5+ p	LHFPL6
529	ABCC9	0	1.92660219393972	0.515	0.032	0	RGS5+ p	ABCC9
530	EPS81	0	1.90217532105443	0.71	0.146	0	RGS5+ p	EPS8
531	CCDC102B	0	1.90027614822953	0.463	0.017	0	RGS5+ p	CCDC102B
532	MT1A2	0	1.89905678614174	0.518	0.117	0	RGS5+ p	MT1A
533	NOTCH31	0	1.8665879022609	0.586	0.057	0	RGS5+ p	NOTCH3
534	COL4A23	0	1.85186262650269	0.595	0.09	0	RGS5+ p	COL4A2
535	EPAS11	0	1.85166901785409	0.697	0.156	0	RGS5+ p	EPAS1
536	FILIP1L3	0	1.83137207908793	0.628	0.158	0	RGS5+ p	FILIP1L
537	MAP1B4	0	1.82508076712512	0.716	0.163	0	RGS5+ p	MAP1B
538	TFPI2	0	1.82437011874023	0.644	0.121	0	RGS5+ p	TFPI
539	IFITM34	0	1.81712430775289	0.921	0.433	0	RGS5+ p	IFITM3
540	TPM13	0	1.79881618973404	0.729	0.18	0	RGS5+ p	TPM1
541	CEBPD4	0	1.7550607835055	0.834	0.411	0	RGS5+ p	CEBPD
542	MCAM1	0	1.74331536389062	0.655	0.123	0	RGS5+ p	MCAM

543	RASD14	0	1.73483376448275	0.551	0.152	0	RGS5+ p	RASD1
544	TIMP34	0	1.72552898595505	0.726	0.202	0	RGS5+ p	TIMP3
545	SPARC3	0	1.71386346596762	0.779	0.255	0	RGS5+ p	SPARC
546	MYO1B2	0	1.70735648910138	0.573	0.073	0	RGS5+ p	MYO1B
547	CARMN1	0	1.70564727931846	0.409	0.019	0	RGS5+ p	CARMN
548	IL62	0	1.66929847006895	0.366	0.128	0	RGS5+ p	IL6
549	ADIRF3	0	1.63368986999578	0.877	0.264	0	RGS5+ p	ADIRF
550	MYL91	0	1.56025812865975	0.694	0.145	0	RGS5+ p	MYL9
551	TM4SF1	0	4.62085162708587	0.925	0.108	0	VEC	TM4SF1
552	SELE	0	4.39011775581363	0.463	0.02	0	VEC	SELE
553	IFI271	0	3.52399915357541	0.921	0.163	0	VEC	IFI27
554	STC1	0	3.16584241332455	0.361	0.015	0	VEC	STC1
555	AQP11	0	3.09996075683418	0.77	0.053	0	VEC	AQP1
556	TSC22D12	0	2.82425711548053	0.843	0.258	0	VEC	TSC22D1
557	GNG112	0	2.80935447435569	0.786	0.098	0	VEC	GNG11
558	DARC	0	2.78981216775848	0.27	0.01	0	VEC	DARC
559	SPRY14	0	2.73898449186426	0.689	0.134	0	VEC	SPRY1
560	CLDN5	0	2.69004615924103	0.605	0.016	0	VEC	CLDN5
561	C2CD4B	0	2.53904623214288	0.454	0.015	0	VEC	C2CD4B
562	ACKR1	0	2.49587533605536	0.254	0.004	0	VEC	ACKR1
563	SPARCL13	0	2.46094974835291	0.893	0.18	0	VEC	SPARCL1
564	PLVAP	0	2.45363871274056	0.613	0.007	0	VEC	PLVAP
565	MCTP12	0	2.40596473072647	0.64	0.073	0	VEC	MCTP1
566	CSF3	0	2.38569801839529	0.337	0.011	0	VEC	CSF3
567	RBP7	0	2.37982791346583	0.422	0.01	0	VEC	RBP7
568	RCAN1	0	2.37226863617029	0.558	0.072	0	VEC	RCAN1
569	ADAMTS91	0	2.35785084472881	0.571	0.048	0	VEC	ADAMTS9
570	CALCRL	0	2.27574269268504	0.557	0.044	0	VEC	CALCRL
571	RAMP2	0	2.22166649535382	0.623	0.039	0	VEC	RAMP2
572	A2M3	0	2.2045663760512	0.683	0.113	0	VEC	A2M
573	PDLIM12	0	2.16595437233151	0.709	0.115	0	VEC	PDLIM1
574	EMP15	0	2.1439526008102	0.83	0.3	0	VEC	EMP1
575	SOX17	0	2.11562661990668	0.477	0.003	0	VEC	SOX17
576	ETS22	0	2.07225494578725	0.789	0.273	0	VEC	ETS2

577	FLT1	0	2.04899531923505	0.491	0.039	0	VEC	FLT1
578	CAV14	0	2.03964267521101	0.788	0.224	0	VEC	CAV1
579	SLC9A3R2	0	1.99036829030377	0.468	0.041	0	VEC	SLC9A3R2
580	IL63	0	1.98653029213192	0.424	0.127	0	VEC	IL6
581	TCF45	0	1.98358276967956	0.795	0.246	0	VEC	TCF4
582	LMCD1	0	1.94708775978173	0.6	0.083	0	VEC	LMCD1
583	EMCN	0	1.91874379429822	0.575	0.026	0	VEC	EMCN
584	ARHGAP292	0	1.90196510758064	0.607	0.077	0	VEC	ARHGAP29
585	NPDC1	0	1.86993381268945	0.605	0.052	0	VEC	NPDC1
586	CRIP23	0	1.83593813395312	0.677	0.134	0	VEC	CRIP2
587	IFITM35	0	1.81906723427023	0.939	0.435	0	VEC	IFITM3
588	PECAM1	0	1.7887405630688	0.415	0.03	0	VEC	PECAM1
589	ZNF385D	0	1.78493721542595	0.461	0.024	0	VEC	ZNF385D
590	RAMP3	0	1.77249716127934	0.444	0.008	0	VEC	RAMP3
591	CD594	0	1.76144851918594	0.798	0.322	0	VEC	CD59
592	EPAS12	0	1.74547205080907	0.669	0.159	0	VEC	EPAS1
593	ADIRF4	0	1.74233059980953	0.814	0.269	0	VEC	ADIRF
594	EGFL7	0	1.73693589993822	0.433	0.02	0	VEC	EGFL7
595	PALMD	0	1.71856596968131	0.454	0.018	0	VEC	PALMD
596	ID14	0	1.71817034223124	0.409	0.124	0	VEC	ID1
597	SOCS35	0	1.68292490136853	0.742	0.435	0	VEC	SOCS3
598	ICAM15	0	1.67386567602106	0.612	0.215	0	VEC	ICAM1
599	GJA13	0	1.66551102224218	0.539	0.12	0	VEC	GJA1
600	ECSCR	0	1.65787722445488	0.475	0.01	0	VEC	ECSCR
601	MALAT14	0	1.42981892533727	0.999	0.999	0	Tc	MALAT1
602	PTPRC4	0	1.33048269754277	0.789	0.57	0	Tc	PTPRC
603	SRSF74	0	1.32603138015463	0.755	0.734	0	Tc	SRSF7
604	MTRNR2L12	0	1.32128444509003	0.768	0.76	0	Tc	MTRNR2L12
605	STK44	1.6668	1.26528955757821	0.666	0.574	5.5000816	Tc	STK4
606	EML43	1.9011	1.3695737430568	0.543	0.474	6.2730021	Tc	EML4
607	CLEC2D3	9.6443	1.1348420445278	0.473	0.349	3.1822621	Tc	CLEC2D
608	SMCHD13	1.4454	1.48062806744763	0.508	0.471	4.7693414	Tc	SMCHD1
609	CHD24	2.0690	1.2500829241857	0.526	0.533	6.8269916	Tc	CHD2
610	HNRNPH1	1.3887	1.01268945545987	0.605	0.692	4.5821680	Tc	HNRNPH1

611	RNF2134	2.8898	1.40602373476933	0.422	0.361	9.5352056	Tc	RNF213
612	IKZF14	2.1927	1.23402158985789	0.377	0.295	7.2353222	Tc	IKZF1
613	ACAP14	5.3673	1.22193773897327	0.367	0.285	1.7710229	Tc	ACAP1
614	RORA3	9.9988	1.2149653823264	0.469	0.399	3.2992087	Tc	RORA
615	SYNE25	7.3053	1.29136598348125	0.408	0.344	2.4104794	Tc	SYNE2
616	ITK4	2.9261	1.14568259067355	0.287	0.195	9.6549731	Tc	ITK
617	NKTR4	9.2532	1.23822919414785	0.435	0.414	3.0532033	Tc	NKTR
618	FUS2	2.7927	0.939093965995657	0.603	0.704	9.2147962	Tc	FUS
619	ANKRD123	2.0730	1.33112679332914	0.553	0.61	6.8402060	Tc	ANKRD12
620	AC058791.1	7.8366	1.26614338642381	0.413	0.375	2.5857911	Tc	AC058791.1
621	PPP2R5C4	4.1470	1.1092979682747	0.439	0.416	1.3683699	Tc	PPP2R5C
622	MBNL14	7.7716	1.02917298473745	0.533	0.609	2.5643212	Tc	MBNL1
623	CNOT6L4	6.4794	0.950533119319221	0.474	0.447	2.1379502	Tc	CNOT6L
624	TSPYL23	2.6911	1.26914154375853	0.465	0.466	8.8798207	Tc	TSPYL2
625	FYB14	1.1945	1.03358691144931	0.357	0.301	3.9415318	Tc	FYB1
626	RNF19A4	4.3136	0.975515392445084	0.438	0.425	1.4233337	Tc	RNF19A
627	AC016831.5	8.0017	1.18910490997222	0.343	0.314	2.6402653	Tc	AC016831.5
628	IKZF34	4.2519	1.10355451465914	0.251	0.194	1.4029768	Tc	IKZF3
629	FYN3	1.0056	0.991301123663634	0.387	0.386	3.3181072	Tc	FYN
630	AC016831.7	4.6424	1.03904711136063	0.292	0.268	1.5318109	Tc	AC016831.7
631	DDX3Y2	3.7337	1.06690683414335	0.406	0.439	1.2319761	Tc	DDX3Y
632	CEMIP23	8.5006	1.05094561239758	0.364	0.36	2.8048651	Tc	CEMIP2
633	GLS3	8.9226	0.957371703737809	0.478	0.57	2.9441195	Tc	GLS
634	BCL11B4	9.9742	0.915275599563468	0.271	0.238	3.2910899	Tc	BCL11B
635	SLC5A33	2.7030	1.23424265592246	0.315	0.3	8.9189381	Tc	SLC5A3
636	NSD33	1.5382	1.04109276323615	0.41	0.451	5.0756328	Tc	NSD3
637	PLCG22	3.2754	1.52764706018505	0.352	0.362	1.0807600	Tc	PLCG2
638	ANKRD283	8.7701	1.22546070999504	0.297	0.293	2.8938126	Tc	ANKRD28
639	CRYBG14	1.4896	0.967207394761305	0.261	0.246	4.9153247	Tc	CRYBG1
640	N4BP2L21	1.4783	0.935113830891148	0.409	0.489	4.8780784	Tc	N4BP2L2
641	KANSL14	9.8175	0.931931345238158	0.335	0.365	3.2394064	Tc	KANSL1
642	MAF 3.00	8.1990	1.0222708273944	0.326	0.339	2.7053466	Tc	MAF
643	USP152	3.2942	0.932596735451589	0.353	0.412	1.0869799	Tc	USP15
644	ATXN13	7.4680	0.935754139378722	0.308	0.338	2.4641452	Tc	ATXN1

645	OGA2	3.9944	0.948868803438454	0.266	0.283	1.3179995	Tc	OGA
646	AAK14	6.7690	0.917990124366622	0.289	0.317	2.2335241	Tc	AAK1
647	PCSK73	2.3996	0.947016854516241	0.265	0.285	0.0007917	Tc	PCSK7
648	KMT2A3	2.9352	0.926848165190465	0.347	0.414	0.0009685	Tc	KMT2A
649	NABP14	1.7456	0.958880491542779	0.318	0.364	0.0576008	Tc	NABP1
650	RNMT2	1.6029	0.931204238404226	0.284	0.327	0.5289004	Tc	RNMT
651	XCL1	0	4.76788797356367	0.813	0.039	0	ILC_NK	XCL1
652	GNLY	0	4.42259940350476	0.651	0.038	0	ILC_NK	GNLY
653	XCL2	0	4.34564839709996	0.664	0.032	0	ILC_NK	XCL2
654	KLRD1	0	2.43467528365319	0.555	0.022	0	ILC_NK	KLRD1
655	GZMB1	0	2.314287294876	0.401	0.046	0	ILC_NK	GZMB
656	NKG71	0	2.05188206485461	0.583	0.098	0	ILC_NK	NKG7
657	CTSW1	0	1.99778309492716	0.573	0.062	0	ILC_NK	CTSW
658	KLRC1	0	1.96967188337752	0.401	0.006	0	ILC_NK	KLRC1
659	AREG2	0	1.75288835015594	0.525	0.17	0	ILC_NK	AREG
660	KLRB12	0	1.64441419028777	0.501	0.122	0	ILC_NK	KLRB1
661	CRTAM1	0	1.58970098045589	0.341	0.062	0	ILC_NK	CRTAM
662	CCL51	0	1.57397736529042	0.574	0.148	0	ILC_NK	CCL5
663	CD72	0	1.55407093485227	0.621	0.156	0	ILC_NK	CD7
664	REL3	0	1.54598069958049	0.942	0.704	0	ILC_NK	REL
665	TNFRSF184	0	1.4753321181558	0.692	0.243	0	ILC_NK	TNFRSF18
666	PIK3R13	0	1.39377717206013	0.684	0.346	0	ILC_NK	PIK3R1
667	SYTL34	0	1.39110063488152	0.652	0.245	0	ILC_NK	SYTL3
668	CLEC2B2	0	1.37856893364807	0.799	0.436	0	ILC_NK	CLEC2B
669	ADGRE52	0	1.37160871843447	0.579	0.218	0	ILC_NK	ADGRE5
670	CD694	0	1.34475891050692	0.781	0.334	0	ILC_NK	CD69
671	HOPX2	0	1.31704468072348	0.454	0.091	0	ILC_NK	HOPX
672	DUSP25	0	1.3061797289873	0.848	0.486	0	ILC_NK	DUSP2
673	IL2RB3	0	1.26581082745367	0.491	0.123	0	ILC_NK	IL2RB
674	RUNX34	0	1.25273697296389	0.787	0.39	0	ILC_NK	RUNX3
675	TRDC	0	1.2497743325698	0.27	0.007	0	ILC_NK	TRDC
676	STK17A5	0	1.21377634200358	0.731	0.407	0	ILC_NK	STK17A
677	METRNL3	0	1.20581760192425	0.659	0.373	0	ILC_NK	METRNL
678	HIST1H4C3	0	1.17996360768849	0.632	0.366	0	ILC_NK	HIST1H4C

679	FAM177A13	0	1.13486095073718	0.676	0.407	0	ILC_NK	FAM177A1
680	GPR653	0	1.13318188773008	0.536	0.194	0	ILC_NK	GPR65
681	CMC11	0	1.1139952178086	0.257	0.091	0	ILC_NK	CMC1
682	TCF71	0	1.08949206958885	0.42	0.123	0	ILC_NK	TCF7
683	B3GNT7	0	1.08418211870008	0.266	0.017	0	ILC_NK	B3GNT7
684	NFKB12	0	1.06283116110129	0.696	0.432	0	ILC_NK	NFKB1
685	IFITM26	0	1.05092774746493	0.822	0.598	0	ILC_NK	IFITM2
686	CD964	0	1.03845268024673	0.523	0.188	0	ILC_NK	CD96
687	HCST6	0	1.03700323406824	0.668	0.35	0	ILC_NK	HCST
688	MATK	0	1.03031548400639	0.332	0.037	0	ILC_NK	MATK
689	PRF1	0	1.02658405736238	0.273	0.034	0	ILC_NK	PRF1
690	TRGC1	0	1.01850038045765	0.268	0.02	0	ILC_NK	TRGC1
691	CST72	0	0.998217070942577	0.548	0.233	0	ILC_NK	CST7
692	RAB8B4	0	0.993994027188043	0.736	0.463	0	ILC_NK	RAB8B
693	SAMD31	0	0.992419004434694	0.291	0.036	0	ILC_NK	SAMD3
694	LINC018712	0	0.974949557937984	0.347	0.097	0	ILC_NK	LINC01871
695	TRGC21	0	0.968932017423875	0.277	0.049	0	ILC_NK	TRGC2
696	VPS37B4	0	0.964401131016146	0.562	0.285	0	ILC_NK	VPS37B
697	GABARAPL1	0	0.962420695517708	0.658	0.391	0	ILC_NK	GABARAPL1
698	PTPN223	0	0.948568575999723	0.496	0.191	0	ILC_NK	PTPN22
699	CD2471	0	0.942096182202864	0.328	0.111	0	ILC_NK	CD247
700	AHI1	1.1525	1.06761563360919	0.408	0.203	3.8028732	ILC_NK	AHI1
701	KRT14	0	5.58097669518395	0.932	0.208	0	Supraba	KRT14
702	DMKN	0	5.24818832326327	0.776	0.046	0	Supraba	DMKN
703	SFN	0	5.2041147068356	0.971	0.098	0	Supraba	SFN
704	KRT1	0	4.99447654884469	0.666	0.037	0	Supraba	KRT1
705	KRT10	0	4.83420579869808	0.829	0.285	0	Supraba	KRT10
706	S100A2	0	4.50959869997311	0.828	0.201	0	Supraba	S100A2
707	PERP	0	4.38228446319702	0.969	0.152	0	Supraba	PERP
708	KRTDAP	0	4.29128074936503	0.552	0.018	0	Supraba	KRTDAP
709	LY6D	0	4.05867072153456	0.779	0.024	0	Supraba	LY6D
710	S100A14	0	3.98490188238559	0.937	0.06	0	Supraba	S100A14
711	LGALS7B	0	3.9843726690773	0.871	0.031	0	Supraba	LGALS7B
712	KRT5	0	3.79796644801845	0.82	0.117	0	Supraba	KRT5

713	TACSTD2	0	3.6313619915386	0.892	0.047	0	Supraba	TACSTD2
714	MIR205HG	0	3.42796666405753	0.766	0.012	0	Supraba	MIR205HG
715	AQP3	0	3.24484602854855	0.89	0.123	0	Supraba	AQP3
716	LYPD3	0	3.12725530407241	0.71	0.069	0	Supraba	LYPD3
717	GNB2L12	0	2.82923134584601	0.828	0.108	0	Supraba	GNB2L1
718	SERPINB5	0	2.76980225550851	0.794	0.021	0	Supraba	SERPINB5
719	DSP	0	2.74616725238925	0.854	0.063	0	Supraba	DSP
720	KRT16	0	2.67089898157187	0.497	0.026	0	Supraba	KRT16
721	KRT15	0	2.61368815716436	0.408	0.052	0	Supraba	KRT15
722	SBSN	0	2.60553222156968	0.433	0.01	0	Supraba	SBSN
723	CCL27	0	2.55949552575699	0.639	0.008	0	Supraba	CCL27
724	CALML5	0	2.52491613799969	0.253	0.008	0	Supraba	CALML5
725	FGFBP1	0	2.48072555336761	0.6	0.016	0	Supraba	FGFBP1
726	FXYD3	0	2.43657337669234	0.773	0.032	0	Supraba	FXYD3
727	S100A163	0	2.33442408450881	0.799	0.093	0	Supraba	S100A16
728	DST3	0	2.25927043995058	0.633	0.213	0	Supraba	DST
729	SERPINB2	0	2.19572317217021	0.605	0.041	0	Supraba	SERPINB2
730	DSC3	0	2.11843144584711	0.704	0.025	0	Supraba	DSC3
731	LGALS7	0	2.07994577908337	0.452	0.009	0	Supraba	LGALS7
732	GLTSCR22	0	2.05392228727719	0.733	0.083	0	Supraba	GLTSCR2
733	KLF52	0	2.02957897494119	0.723	0.089	0	Supraba	KLF5
734	RNF144B2	0	2.01448333359011	0.412	0.094	0	Supraba	RNF144B
735	ERRFI13	0	1.95057230802315	0.616	0.153	0	Supraba	ERRFI1
736	ALDOA2	0	1.89689430327775	0.728	0.134	0	Supraba	ALDOA
737	CLDN1	0	1.89275923732049	0.554	0.045	0	Supraba	CLDN1
738	RPL75	0	1.82920800201564	0.985	0.904	0	Supraba	RPL7
739	IRF6	0	1.81517572655062	0.607	0.018	0	Supraba	IRF6
740	EIF4A15	0	1.79114223241456	0.9	0.487	0	Supraba	EIF4A1
741	HOPX3	0	1.76203250777694	0.585	0.089	0	Supraba	HOPX
742	MPZL2	0	1.73872142727196	0.529	0.022	0	Supraba	MPZL2
743	ATP5G22	0	1.71108795168054	0.674	0.076	0	Supraba	ATP5G2
744	CXADR	0	1.71030565034889	0.597	0.022	0	Supraba	CXADR
745	NHP2L12	0	1.70850874924694	0.625	0.053	0	Supraba	NHP2L1
746	COL17A1	0	1.70053202947764	0.419	0.023	0	Supraba	COL17A1

747	CLTB2	0	1.69853712965747	0.787	0.271	0	Supraba	CLTB
748	SPINT21	0	1.69070981792757	0.661	0.091	0	Supraba	SPINT2
749	PHLDA23	0	1.68855302828963	0.616	0.22	0	Supraba	PHLDA2
750	MYC6	0	1.67826002406291	0.73	0.25	0	Supraba	MYC
751	MS4A1	0	4.07370185859762	0.971	0.009	0	BC	MS4A1
752	BANK1	0	2.81321820058107	0.836	0.008	0	BC	BANK1
753	CD375	0	2.34098701740075	0.939	0.361	0	BC	CD37
754	LY9	0	2.33174947890381	0.672	0.093	0	BC	LY9
755	CD79A	0	2.07345726851767	0.677	0.011	0	BC	CD79A
756	MEF2C5	0	2.00135878874789	0.778	0.156	0	BC	MEF2C
757	SWAP702	0	1.82270270461117	0.684	0.121	0	BC	SWAP70
758	CCR71	0	1.82071151557367	0.493	0.096	0	BC	CCR7
759	RALGPS2	0	1.77737232413247	0.602	0.044	0	BC	RALGPS2
760	TNFRSF13B	0	1.74560390282892	0.529	0.009	0	BC	TNFRSF13B
761	IGHM	0	1.68432140810676	0.272	0.007	0	BC	IGHM
762	LINC00926	0	1.6452222931062	0.519	0.006	0	BC	LINC00926
763	CD832	0	1.60985017512494	0.818	0.253	0	BC	CD83
764	PARP143	0	1.59039397378308	0.701	0.198	0	BC	PARP14
765	XIST	0	1.58925005908667	0.418	0.051	0	BC	XIST
766	TNFRSF13C	0	1.58054834746853	0.536	0.01	0	BC	TNFRSF13C
767	LTB3	0	1.56719300342792	0.814	0.265	0	BC	LTB
768	HLA-DQA12	0	1.50114971571299	0.923	0.249	0	BC	HLA-DQA1
769	CD525	0	1.45647176592788	0.875	0.386	0	BC	CD52
770	BCL11A1	0	1.44262931128644	0.488	0.049	0	BC	BCL11A
771	IRF82	0	1.43554653067327	0.604	0.096	0	BC	IRF8
772	NFKBID3	0	1.42223879313498	0.594	0.186	0	BC	NFKBID
773	ADAM28	0	1.41917353884995	0.474	0.031	0	BC	ADAM28
774	LAPTM53	0	1.37418549361664	0.881	0.415	0	BC	LAPTM5
775	HLA-DRA2	0	1.36057838810261	0.991	0.487	0	BC	HLA-DRA
776	PHACTR12	0	1.35572901796091	0.474	0.111	0	BC	PHACTR1
777	CD70	0	1.35525248474405	0.428	0.044	0	BC	CD70
778	SMIM14	0	1.3459358868224	0.481	0.095	0	BC	SMIM14
779	BCL21	0	1.33727077572672	0.656	0.238	0	BC	BCL2
780	CD485	0	1.32677680915115	0.827	0.349	0	BC	CD48

781	CD79B	0	1.31972636454066	0.392	0.031	0	BC	CD79B
782	PRDM21	0	1.30239303439994	0.75	0.338	0	BC	PRDM2
783	CD743	0	1.30204067156564	0.992	0.583	0	BC	CD74
784	RPS294	0	1.30174436035058	0.992	0.954	0	BC	RPS29
785	HLA-DMB2	0	1.28860240237043	0.552	0.106	0	BC	HLA-DMB
786	LYN2	0	1.26779932963874	0.536	0.095	0	BC	LYN
787	ST6GAL1	0	1.25664331153522	0.448	0.04	0	BC	ST6GAL1
788	POU2F21	0	1.24119443775203	0.46	0.076	0	BC	POU2F2
789	SYNGR22	0	1.23106255780255	0.575	0.157	0	BC	SYNGR2
790	CD402	0	1.22156514708909	0.511	0.099	0	BC	CD40
791	PIKFYVE	0	1.22043971987545	0.496	0.109	0	BC	PIKFYVE
792	ARHGAP24	0	1.21916065684426	0.439	0.041	0	BC	ARHGAP24
793	TMEM156	0	1.21725404440543	0.415	0.03	0	BC	TMEM156
794	IER53	0	1.20637193034285	0.789	0.458	0	BC	IER5
795	COBLL12	0	1.20138739741695	0.469	0.067	0	BC	COBLL1
796	HLA-DQB12	0	1.15910793996264	0.894	0.309	0	BC	HLA-DQB1
797	CCDC501	0	1.15484691395671	0.499	0.135	0	BC	CCDC50
798	RIPOR2	0	1.14854969289273	0.513	0.11	0	BC	RIPOR2
799	RPS273	0	1.11985243172987	0.993	0.98	0	BC	RPS27
800	BCL2A12	0	1.11898761363154	0.599	0.191	0	BC	BCL2A1
801	KRT171	0	4.99118795194765	0.821	0.119	0	Basal ke	KRT17
802	KRT51	0	3.73005716926308	0.882	0.118	0	Basal ke	KRT5
803	DSP1	0	3.70760248015657	0.788	0.068	0	Basal ke	DSP
804	KRT151	0	3.51280923987983	0.729	0.046	0	Basal ke	KRT15
805	DST4	0	3.06078137713857	0.786	0.211	0	Basal ke	DST
806	KRT141	0	2.96370361497919	0.921	0.211	0	Basal ke	KRT14
807	SPON21	0	2.71217811707978	0.613	0.075	0	Basal ke	SPON2
808	SOX43	0	2.7016251436436	0.805	0.315	0	Basal ke	SOX4
809	DAPL11	0	2.64259691449427	0.57	0.036	0	Basal ke	DAPL1
810	EDIL3	0	2.61666353212792	0.541	0.032	0	Basal ke	EDIL3
811	GJB6	0	2.53484113617689	0.603	0.011	0	Basal ke	GJB6
812	IRX21	0	2.49357265267839	0.677	0.07	0	Basal ke	IRX2
813	S100A21	0	2.45385801934551	0.749	0.206	0	Basal ke	S100A2
814	CLDN11	0	2.28328998086465	0.618	0.046	0	Basal ke	CLDN1

815	TFAP2B	0	2.26473327329529	0.556	0.012	0	Basal ke	TFAP2B
816	PERP1	0	2.2414993571333	0.841	0.158	0	Basal ke	PERP
817	PTCH1	0	2.21392974051517	0.491	0.028	0	Basal ke	PTCH1
818	BNC2	0	2.2027161343959	0.566	0.02	0	Basal ke	BNC2
819	NFIB6	0	2.10988571016691	0.677	0.146	0	Basal ke	NFIB
820	ABI3BP2	0	2.04416150773796	0.533	0.084	0	Basal ke	ABI3BP
821	EPCAM	0	2.03166774837754	0.482	0.011	0	Basal ke	EPCAM
822	S100A141	0	2.01208435760655	0.692	0.069	0	Basal ke	S100A14
823	LMO3	0	2.01118183685133	0.385	0.01	0	Basal ke	LMO3
824	CD242	0	1.9668417785186	0.487	0.023	0	Basal ke	CD24
825	CALD15	0	1.88286714911771	0.78	0.304	0	Basal ke	CALD1
826	CXADR1	0	1.85061738922884	0.537	0.025	0	Basal ke	CXADR
827	DSC31	0	1.79216856741172	0.522	0.032	0	Basal ke	DSC3
828	MYLK2	0	1.77455547188863	0.367	0.084	0	Basal ke	MYLK
829	KTN12	0	1.7722084085142	0.823	0.527	0	Basal ke	KTN1
830	EFNA13	0	1.75108425280404	0.482	0.076	0	Basal ke	EFNA1
831	TP631	0	1.73204879350773	0.491	0.018	0	Basal ke	TP63
832	GJA15	0	1.72585055497845	0.59	0.124	0	Basal ke	GJA1
833	SOX9	0	1.71885175933958	0.45	0.025	0	Basal ke	SOX9
834	COL17A11	0	1.67167247307784	0.419	0.024	0	Basal ke	COL17A1
835	BCAM3	0	1.66486919846834	0.566	0.079	0	Basal ke	BCAM
836	ARL4A3	0	1.5816914223216	0.62	0.271	0	Basal ke	ARL4A
837	SFRP11	0	1.57972401650606	0.278	0.043	0	Basal ke	SFRP1
838	PCNT	0	1.56084516606197	0.389	0.05	0	Basal ke	PCNT
839	CHCHD7	0	1.55306910118166	0.516	0.12	0	Basal ke	CHCHD7
840	CREB51	0	1.52497248635845	0.502	0.089	0	Basal ke	CREB5
841	ITGB8	0	1.52446385816902	0.431	0.028	0	Basal ke	ITGB8
842	PTCH2	0	1.50905565636841	0.388	0.026	0	Basal ke	PTCH2
843	GJB2	0	1.49847541452479	0.409	0.03	0	Basal ke	GJB2
844	DAAM12	0	1.4833264687072	0.532	0.145	0	Basal ke	DAAM1
845	PIK3C2G	0	1.48315459962512	0.393	0.003	0	Basal ke	PIK3C2G
846	EDN1	0	1.47178377630876	0.36	0.022	0	Basal ke	EDN1
847	SOSTDC1	0	1.46033460319007	0.279	0.007	0	Basal ke	SOSTDC1
848	PALLD4	0	1.44451135742471	0.49	0.114	0	Basal ke	PALLD

849	CTSV	0	1.42979807518816	0.25	0.012	0	Basal ke	CTSV
850	CASC15	0	1.41737162054627	0.4	0.014	0	Basal ke	CASC15
851	SERPINB21	0	5.16885528980248	0.526	0.046	0	Monocyt	SERPINB2
852	IL1B2	0	4.77504233409099	0.869	0.122	0	Monocyt	IL1B
853	EREG2	0	4.56773061079668	0.911	0.076	0	Monocyt	EREG
854	THBS14	0	4.29349108588412	0.773	0.225	0	Monocyt	THBS1
855	CXCL82	0	4.12801256418339	0.949	0.273	0	Monocyt	CXCL8
856	S100A91	0	4.0957355450031	0.864	0.094	0	Monocyt	S100A9
857	S100A8	0	3.41238029005307	0.783	0.078	0	Monocyt	S100A8
858	TIMP15	0	3.33277382658996	0.968	0.488	0	Monocyt	TIMP1
859	AQP9	0	3.11503351918304	0.815	0.013	0	Monocyt	AQP9
860	BCL2A13	0	3.06632425799644	0.917	0.188	0	Monocyt	BCL2A1
861	CXCL33	0	3.06335901985058	0.788	0.214	0	Monocyt	CXCL3
862	CCL20	0	3.00804258684543	0.474	0.09	0	Monocyt	CCL20
863	LYZ2	0	3.00118268020535	0.956	0.183	0	Monocyt	LYZ
864	SOD26	0	2.99538387753592	0.983	0.558	0	Monocyt	SOD2
865	PTGS23	0	2.94660834842722	0.658	0.13	0	Monocyt	PTGS2
866	IL1RN3	0	2.81535794048853	0.635	0.093	0	Monocyt	IL1RN
867	CTSS3	0	2.58404507733825	0.907	0.247	0	Monocyt	CTSS
868	CXCL25	0	2.57581265193771	0.898	0.296	0	Monocyt	CXCL2
869	BASP13	0	2.50103072952671	0.802	0.217	0	Monocyt	BASP1
870	PPIF2	0	2.47085783761669	0.803	0.195	0	Monocyt	PPIF
871	G0S23	0	2.4649231825029	0.798	0.198	0	Monocyt	G0S2
872	FCER1G3	0	2.45463182993292	0.906	0.152	0	Monocyt	FCER1G
873	MARCKS6	0	2.44978186426655	0.9	0.346	0	Monocyt	MARCKS
874	C15orf482	0	2.44585023738643	0.662	0.133	0	Monocyt	C15orf48
875	ATP2B14	0	2.42790302604997	0.893	0.375	0	Monocyt	ATP2B1
876	SERPINB12	0	2.42670045657624	0.831	0.234	0	Monocyt	SERPINB1
877	TYROBP3	0	2.41436181027979	0.935	0.17	0	Monocyt	TYROBP
878	FTH13	0	2.39258912538597	1	0.983	0	Monocyt	FTH1
879	S100A12	0	2.34939094620001	0.609	0.003	0	Monocyt	S100A12
880	SERPINB93	0	2.3237556894068	0.896	0.38	0	Monocyt	SERPINB9
881	ATP13A32	0	2.31165418928147	0.907	0.291	0	Monocyt	ATP13A3
882	SLC7A111	0	2.21396221833977	0.443	0.048	0	Monocyt	SLC7A11

883	KYNU3	0	2.21250390493512	0.759	0.082	0	Monocyt	KYNU
884	C5AR12	0	2.18467974787192	0.751	0.086	0	Monocyt	C5AR1
885	SLC11A1	0	2.15668751281532	0.651	0.021	0	Monocyt	SLC11A1
886	CD300E1	0	2.15098615615594	0.646	0.027	0	Monocyt	CD300E
887	CD932	0	2.14484119635129	0.669	0.069	0	Monocyt	CD93
888	HIF1A3	0	2.13359261694405	0.865	0.384	0	Monocyt	HIF1A
889	FCN1	0	2.12737992821114	0.65	0.021	0	Monocyt	FCN1
890	SAT13	0	2.12631855254798	0.996	0.843	0	Monocyt	SAT1
891	OLR12	0	2.06035157215161	0.436	0.043	0	Monocyt	OLR1
892	PLAUR3	0	2.04809708177113	0.88	0.318	0	Monocyt	PLAUR
893	CCL31	0	2.03582773653455	0.394	0.092	0	Monocyt	CCL3
894	ANXA55	0	2.02156433358723	0.93	0.542	0	Monocyt	ANXA5
895	GK3	0	2.00481392920054	0.748	0.152	0	Monocyt	GK
896	ITGAX2	0	1.97017013161743	0.71	0.084	0	Monocyt	ITGAX
897	VCAN4	0	1.94983316680396	0.682	0.153	0	Monocyt	VCAN
898	SLC25A374	0	1.94453835850219	0.728	0.21	0	Monocyt	SLC25A37
899	TNFRSF1B4	0	1.93754282281821	0.858	0.369	0	Monocyt	TNFRSF1B
900	FTL3	0	1.92868511329075	0.998	0.962	0	Monocyt	FTL
901	TPSAB1	0	6.08750091431936	0.919	0.035	0	MastC	TPSAB1
902	TPSB2	0	5.26614976944717	0.755	0.032	0	MastC	TPSB2
903	GATA2	0	4.73972855670069	0.823	0.028	0	MastC	GATA2
904	CPA3	0	4.65189792635313	0.767	0.011	0	MastC	CPA3
905	HPGD	0	4.57823593531994	0.789	0.063	0	MastC	HPGD
906	CTSG	0	4.54427223756633	0.646	0.017	0	MastC	CTSG
907	ADCYAP1	0	4.22839070617712	0.511	0.007	0	MastC	ADCYAP1
908	HDC	0	3.81435436234703	0.696	0.008	0	MastC	HDC
909	GLUL5	0	3.60564555925552	0.807	0.384	0	MastC	GLUL
910	KIT	0	3.46683163298305	0.641	0.023	0	MastC	KIT
911	HPGDS	0	3.17551725206878	0.521	0.008	0	MastC	HPGDS
912	IL1RL1	0	3.14782065574649	0.615	0.027	0	MastC	IL1RL1
913	RGS23	0	3.14737145239979	0.843	0.437	0	MastC	RGS2
914	SGK13	0	2.84955227705726	0.663	0.288	0	MastC	SGK1
915	LTC4S	0	2.78720637864792	0.441	0.06	0	MastC	LTC4S
916	GCSAML	0	2.69157323070548	0.441	0.003	0	MastC	GCSAML

917	SLC18A2	0	2.64564183006913	0.447	0.008	0	MastC	SLC18A2
918	FOSB5	0	2.60936572575186	0.715	0.501	0	MastC	FOSB
919	ARHGAP182	0	2.56883473978621	0.543	0.124	0	MastC	ARHGAP18
920	ACSL42	0	2.50027458223343	0.524	0.155	0	MastC	ACSL4
921	MS4A2	0	2.46732861236622	0.408	0.003	0	MastC	MS4A2
922	PTGS24	0	2.41158203932688	0.429	0.137	0	MastC	PTGS2
923	PAG14	0	2.20158799778318	0.496	0.206	0	MastC	PAG1
924	BMP2K1	0	1.98523185254504	0.392	0.094	0	MastC	BMP2K
925	UBB3	0	1.92414785390649	0.877	0.844	0	MastC	UBB
926	VWA5A	0	1.88512965792702	0.321	0.021	0	MastC	VWA5A
927	HSPH14	0	1.85221956235427	0.823	0.682	0	MastC	HSPH1
928	RGS131	0	1.80025368853023	0.302	0.01	0	MastC	RGS13
929	RHEX	0	1.7949308229474	0.267	0.003	0	MastC	RHEX
930	CAVIN2	0	1.78800384543271	0.254	0.022	0	MastC	CAVIN2
931	LEO1	0	1.74312879737252	0.29	0.058	0	MastC	LEO1
932	GALC	0	1.6917309776375	0.291	0.056	0	MastC	GALC
933	HSP90AA15	0	1.64594498260119	0.969	0.959	0	MastC	HSP90AA1
934	ADGRE21	0	1.60177754496876	0.262	0.039	0	MastC	ADGRE2
935	DUSP62	9.3976	1.94661863846943	0.351	0.11	3.1008545	MastC	DUSP6
936	TENT5A5	6.9812	2.2134664052348	0.46	0.186	2.3035342	MastC	TENT5A
937	LAPTM55	9.8278	1.69885709563807	0.655	0.425	3.2428137	MastC	LAPTM5
938	DNAJB16	6.9027	1.87416903150241	0.826	0.752	2.2776176	MastC	DNAJB1
939	CDC42EP35	1.0777	1.96569312957242	0.53	0.283	3.5561779	MastC	CDC42EP3
940	CAPG5	5.8388	1.68423808166319	0.418	0.198	1.9265805	MastC	CAPG
941	CPM3	4.4449	1.61170108482063	0.26	0.081	1.4666440	MastC	CPM
942	RRAD2	4.3859	1.95940906904892	0.281	0.093	1.4471978	MastC	RRAD
943	VEGFA6	1.2418	1.90766115371524	0.484	0.256	4.0975110	MastC	VEGFA
944	PLIN24	2.2322	1.62595867343347	0.606	0.401	7.3655944	MastC	PLIN2
945	SLC44A12	1.0935	1.57710255854991	0.31	0.114	3.6084028	MastC	SLC44A1
946	HSPA1B6	3.9921	1.66643959623204	0.813	0.702	1.3172544	MastC	HSPA1B
947	TNFAIP35	4.8802	1.63467320545886	0.669	0.563	1.6102948	MastC	TNFAIP3
948	NFKBIZ3	3.6685	1.68489810022907	0.55	0.409	1.2104699	MastC	NFKBIZ
949	GNS 2.00	1.8340	1.58163889585433	0.268	0.108	6.0517433	MastC	GNS
950	CPEB45	5.6964	1.63552974168923	0.414	0.256	1.8796115	MastC	CPEB4

951	DCT	0	5.07617280173123	0.786	0.023	0	Melanoc	DCT
952	PMEL	0	4.49240461232203	0.909	0.043	0	Melanoc	PMEL
953	TYRP1	0	4.37836448896651	0.841	0.032	0	Melanoc	TYRP1
954	MITF	0	4.3648775297787	0.884	0.07	0	Melanoc	MITF
955	MLANA	0	3.79419078192113	0.886	0.032	0	Melanoc	MLANA
956	IFI272	0	3.33655224710305	0.783	0.179	0	Melanoc	IFI27
957	PDLIM31	0	2.94848472337057	0.703	0.049	0	Melanoc	PDLIM3
958	CRYAB5	0	2.89701893588777	0.885	0.234	0	Melanoc	CRYAB
959	GCNT2	0	2.71404236228067	0.723	0.02	0	Melanoc	GCNT2
960	PHACTR14	0	2.6410657825678	0.735	0.111	0	Melanoc	PHACTR1
961	TRPM1	0	2.53100824216055	0.649	0.005	0	Melanoc	TRPM1
962	FMN1	0	2.44430142222226	0.706	0.016	0	Melanoc	FMN1
963	CDC42EP36	0	2.21355176034964	0.797	0.277	0	Melanoc	CDC42EP3
964	TFAP2A2	0	2.15729708864752	0.659	0.031	0	Melanoc	TFAP2A
965	GPNMB6	0	2.0607166375331	0.792	0.163	0	Melanoc	GPNMB
966	PLEKHA51	0	2.02151723344576	0.652	0.096	0	Melanoc	PLEKHA5
967	QPCT1	0	2.00193375498247	0.589	0.063	0	Melanoc	QPCT
968	FRZB1	0	1.98461045582056	0.565	0.051	0	Melanoc	FRZB
969	KIT1	0	1.97518105346031	0.618	0.021	0	Melanoc	KIT
970	NSG11	0	1.90256148737118	0.603	0.025	0	Melanoc	NSG1
971	EDNRB4	0	1.8391445008419	0.636	0.093	0	Melanoc	EDNRB
972	SNCA	0	1.83600927776159	0.599	0.04	0	Melanoc	SNCA
973	GPM6B1	0	1.82560659427236	0.648	0.096	0	Melanoc	GPM6B
974	S100A1	0	1.81419760869585	0.382	0.022	0	Melanoc	S100A1
975	PAX3	0	1.79115798797868	0.547	0.019	0	Melanoc	PAX3
976	CPEB2	0	1.78988842798887	0.583	0.094	0	Melanoc	CPEB2
977	TYR	0	1.72642783825426	0.5	0.004	0	Melanoc	TYR
978	OSTM1	0	1.71864052702663	0.594	0.089	0	Melanoc	OSTM1
979	CDH19	0	1.67866936044511	0.468	0.007	0	Melanoc	CDH19
980	SLC7A8	0	1.65872358237281	0.512	0.037	0	Melanoc	SLC7A8
981	MCOLN3	0	1.65340885534431	0.453	0.013	0	Melanoc	MCOLN3
982	TUBB2B	0	1.64357754217643	0.46	0.039	0	Melanoc	TUBB2B
983	APOE7	0	1.63672343628038	0.843	0.3	0	Melanoc	APOE
984	CD595	0	1.63203736807398	0.802	0.331	0	Melanoc	CD59

985	TEX411	0	1.6071700588566	0.491	0.031	0	Melanoc	TEX41
986	SOX10	0	1.60086285482318	0.483	0.004	0	Melanoc	SOX10
987	ZNF1062	0	1.59964394679533	0.665	0.226	0	Melanoc	ZNF106
988	DST5	0	1.58343317713598	0.732	0.215	0	Melanoc	DST
989	MYO10	0	1.58309804275049	0.526	0.046	0	Melanoc	MYO10
990	GSTP17	0	1.57119716058648	0.825	0.482	0	Melanoc	GSTP1
991	CHCHD6	0	1.54443272328548	0.413	0.028	0	Melanoc	CHCHD6
992	PLP1	0	1.52323609950699	0.407	0.018	0	Melanoc	PLP1
993	SPTSSA6	0	1.52037749811934	0.66	0.179	0	Melanoc	SPTSSA
994	C4orf48	0	1.52010039014537	0.573	0.15	0	Melanoc	C4orf48
995	CYB561A3	0	1.50907743409393	0.424	0.056	0	Melanoc	CYB561A3
996	SOX45	0	1.48614545708967	0.789	0.318	0	Melanoc	SOX4
997	ITPR21	0	1.47968813433826	0.48	0.094	0	Melanoc	ITPR2
998	IFI62	0	1.476968982038	0.44	0.129	0	Melanoc	IFI6
999	CYSLTR2	0	1.4230630403481	0.348	0.021	0	Melanoc	CYSLTR2
100	SLCO4A1-A	0	1.4042688045367	0.385	0.004	0	Melanoc	SLCO4A1-A
100	CLU1	0	6.59065712661464	0.789	0.171	0	Chondro	CLU
100	C2orf40	0	5.52143387237343	0.693	0.023	0	Chondro	C2orf40
100	CYTL1	0	5.29737037624442	0.596	0.009	0	Chondro	CYTL1
100	APOD2	0	5.0947020700734	0.802	0.223	0	Chondro	APOD
100	COCH	0	4.65344485343183	0.549	0.023	0	Chondro	COCH
100	RBP4	0	3.85432295356444	0.602	0.007	0	Chondro	RBP4
100	SNORC	0	3.49361395501006	0.591	0.002	0	Chondro	SNORC
100	FGFBP2	0	3.47345562907293	0.588	0.021	0	Chondro	FGFBP2
100	PLA2G2A1	0	3.34482424000627	0.335	0.046	0	Chondro	PLA2G2A
101	SERPINA13	0	3.33663029386008	0.569	0.049	0	Chondro	SERPINA1
101	SOD35	0	3.10261500443301	0.727	0.194	0	Chondro	SOD3
101	MGP4	0	3.09902882466575	0.792	0.295	0	Chondro	MGP
101	MT1X6	0	2.89567199293784	0.762	0.278	0	Chondro	MT1X
101	NNMT5	0	2.72618726335502	0.857	0.238	0	Chondro	NNMT
101	FN14	0	2.56557273337263	0.759	0.131	0	Chondro	FN1
101	MEG33	0	2.49344448209784	0.894	0.148	0	Chondro	MEG3
101	SLPI2	0	2.48103249896551	0.555	0.036	0	Chondro	SLPI
101	COL9A3	0	2.44648325805506	0.576	0.006	0	Chondro	COL9A3

101	S100A15	0	2.35140431221102	0.498	0.021	0	Chondro	S100A1
102	BGN 6.00	0	2.34798659074876	0.636	0.127	0	Chondro	BGN
102	SCRG1	0	2.33557299578784	0.485	0.021	0	Chondro	SCRG1
102	CST34	0	2.33359929037315	0.951	0.514	0	Chondro	CST3
102	WIF1	0	2.27395073244217	0.465	0.015	0	Chondro	WIF1
102	COL2A1	0	2.23460032444127	0.435	0.001	0	Chondro	COL2A1
102	SCGB3A2	0	2.22213789974459	0.259	0	0	Chondro	SCGB3A2
102	TM4SF12	0	2.20097525431534	0.684	0.128	0	Chondro	TM4SF1
102	TSC22D17	0	2.17639137748805	0.772	0.27	0	Chondro	TSC22D1
102	MIA	0	2.14340542465968	0.565	0.005	0	Chondro	MIA
102	CTGF6	0	2.1202630963609	0.649	0.161	0	Chondro	CTGF
103	FGF21	0	2.03398165708235	0.584	0.042	0	Chondro	FGF2
103	MT2A4	0	1.97911610727129	0.858	0.645	0	Chondro	MT2A
103	GPX32	0	1.95115753708751	0.556	0.085	0	Chondro	GPX3
103	RFLNA	0	1.94483901420315	0.529	0.003	0	Chondro	RFLNA
103	COL9A2	0	1.93329749877662	0.526	0.022	0	Chondro	COL9A2
103	S100A135	0	1.89485571188955	0.779	0.196	0	Chondro	S100A13
103	IL64	0	1.88600367774005	0.547	0.131	0	Chondro	IL6
103	CEBPD5	0	1.87811612596779	0.94	0.42	0	Chondro	CEBPD
103	C9orf37	0	1.83888648630203	0.734	0.152	0	Chondro	C9orf3
103	LINC01436	0	1.8226443330038	0.402	0.016	0	Chondro	LINC01436
104	IER310	0	1.80443153691736	0.812	0.461	0	Chondro	IER3
104	TUBB2A6	0	1.70214715856943	0.717	0.256	0	Chondro	TUBB2A
104	ACAN	0	1.67618847556945	0.461	0.007	0	Chondro	ACAN
104	FRZB2	0	1.65151852428904	0.484	0.053	0	Chondro	FRZB
104	MSX13	0	1.62527887788367	0.623	0.092	0	Chondro	MSX1
104	PTX31	0	1.61615366619071	0.368	0.051	0	Chondro	PTX3
104	MT1A3	0	1.59493272857507	0.496	0.128	0	Chondro	MT1A
104	NRP25	0	1.58380410539687	0.632	0.139	0	Chondro	NRP2
104	MT1E7	0	1.58299228765797	0.524	0.162	0	Chondro	MT1E
104	S100B1	0	1.55752826006892	0.522	0.041	0	Chondro	S100B
105	PRELP2	0	1.55416500772835	0.539	0.053	0	Chondro	PRELP
105	HLA-DPB13	0	3.45766458782067	0.999	0.416	0	DC2	HLA-DPB1
105	HLA-DPA13	0	3.42645512396262	0.998	0.407	0	DC2	HLA-DPA1

105	CPVL3	0	3.09220037973741	0.89	0.087	0	DC2	CPVL
105	CD744	0	3.01279421542905	1	0.59	0	DC2	CD74
105	HLA-DRA4	0	2.8036437159205	1	0.496	0	DC2	HLA-DRA
105	TXN6	0	2.76052126769937	0.96	0.666	0	DC2	TXN
105	HLA-DRB13	0	2.75353842727868	0.999	0.455	0	DC2	HLA-DRB1
105	HLA-DRB54	0	2.69109038026844	0.792	0.231	0	DC2	HLA-DRB5
105	HLA-DQA13	0	2.64552326941928	0.988	0.26	0	DC2	HLA-DQA1
106	DNASE1L3	0	2.59302833988216	0.833	0.019	0	DC2	DNASE1L3
106	C1orf542	0	2.59205087508369	0.923	0.08	0	DC2	C1orf54
106	CST35	0	2.52737865239077	0.995	0.516	0	DC2	CST3
106	PPIF3	0	2.46853764998852	0.823	0.201	0	DC2	PPIF
106	S100B2	0	2.45833968861617	0.714	0.042	0	DC2	S100B
106	HLA-DQB13	0	2.4014090732121	0.981	0.318	0	DC2	HLA-DQB1
106	LYZ3	0	2.35982900501939	0.969	0.191	0	DC2	LYZ
106	CTSZ3	0	2.30728383193237	0.939	0.318	0	DC2	CTSZ
106	SGK15	0	2.09462890409546	0.844	0.287	0	DC2	SGK1
106	BASP14	0	2.06845460928201	0.903	0.221	0	DC2	BASP1
107	LGALS22	0	2.05801647969909	0.823	0.06	0	DC2	LGALS2
107	DAPP12	0	1.89991463004229	0.772	0.102	0	DC2	DAPP1
107	IRF84	0	1.83091823808794	0.79	0.103	0	DC2	IRF8
107	SAT15	0	1.78599538690318	0.992	0.844	0	DC2	SAT1
107	CD835	0	1.70671299811018	0.915	0.262	0	DC2	CD83
107	VMO12	0	1.70330630339885	0.641	0.047	0	DC2	VMO1
107	CLEC7A3	0	1.67555550082432	0.813	0.073	0	DC2	CLEC7A
107	IDO1	0	1.64984112964622	0.486	0.012	0	DC2	IDO1
107	RAB11FIP17	0	1.5990962817889	0.859	0.292	0	DC2	RAB11FIP1
107	PLEK4	0	1.56891193309319	0.854	0.16	0	DC2	PLEK
108	CLEC9A	0	1.55253777557474	0.695	0.001	0	DC2	CLEC9A
108	GPR1571	0	1.53451826491332	0.577	0.056	0	DC2	GPR157
108	AC020656.1	0	1.47864074838305	0.594	0.079	0	DC2	AC020656.1
108	CXCL163	0	1.44502225574637	0.761	0.124	0	DC2	CXCL16
108	SNX39	0	1.42194287777961	0.932	0.456	0	DC2	SNX3
108	HLA-DQA23	0	1.40682180487448	0.611	0.094	0	DC2	HLA-DQA2
108	WDFY4	0	1.37160873318215	0.659	0.013	0	DC2	WDFY4

108	HLA-DMA4	0	1.33621215384232	0.857	0.187	0	DC2	HLA-DMA
108	ACTB5	0	1.32400112693133	0.997	0.917	0	DC2	ACTB
108	NRARP4	0	1.31834978033689	0.623	0.107	0	DC2	NRARP
109	CPNE31	0	1.303514204151	0.747	0.167	0	DC2	CPNE3
109	SHTN1	0	1.29400900473765	0.637	0.048	0	DC2	SHTN1
109	MXD13	0	1.2922272110015	0.726	0.227	0	DC2	MXD1
109	RGS105	0	1.28643220484236	0.839	0.287	0	DC2	RGS10
109	S100A105	0	1.23356105489039	0.99	0.806	0	DC2	S100A10
109	C1orf1622	0	1.22900933115	0.697	0.106	0	DC2	C1orf162
109	PLAUR5	0	1.21589381986808	0.849	0.324	0	DC2	PLAUR
109	ID25	4.4946	1.68364820664384	0.885	0.551	1.4830582	DC2	ID2
109	ZFAND2A4	4.0381	1.47705649211156	0.645	0.279	1.3324215	DC2	ZFAND2A
109	IER54	2.2876	1.41097397651781	0.797	0.463	7.5484594	DC2	IER5
110	HSPA67	8.3825	1.2712238809002	0.508	0.293	2.7659076	DC2	HSPA6
110	CCL211	0	6.50694082987718	0.928	0.031	0	LEC	CCL21
110	TFF3	0	4.82875983175065	0.918	0.01	0	LEC	TFF3
110	FABP4	0	3.96977973798143	0.762	0.033	0	LEC	FABP4
110	CLDN51	0	3.51434897228565	0.838	0.03	0	LEC	CLDN5
110	TFPI5	0	3.50129019255492	0.946	0.135	0	LEC	TFPI
110	MMRN1	0	3.48246549988087	0.8	0.005	0	LEC	MMRN1
110	GNG114	0	3.31490209288042	0.895	0.116	0	LEC	GNG11
110	ANGPT23	0	2.93261382174279	0.616	0.068	0	LEC	ANGPT2
110	PPFIBP13	0	2.78161332311526	0.838	0.101	0	LEC	PPFIBP1
111	AKAP126	0	2.66354224486309	0.651	0.106	0	LEC	AKAP12
111	LYVE1	0	2.44133979306039	0.571	0.013	0	LEC	LYVE1
111	RAMP21	0	2.41866052489429	0.708	0.054	0	LEC	RAMP2
111	PROX1	0	2.41751200240576	0.665	0.022	0	LEC	PROX1
111	C2CD4B1	0	2.32623971967264	0.419	0.027	0	LEC	C2CD4B
111	IGFBP75	0	2.31759598881207	0.947	0.348	0	LEC	IGFBP7
111	CAVIN21	0	2.21235370609766	0.491	0.021	0	LEC	CAVIN2
111	ECSCR1	0	2.18512106122603	0.635	0.021	0	LEC	ECSCR
111	SNCG2	0	2.15714858014658	0.589	0.038	0	LEC	SNCG
111	TM4SF13	0	2.08751573087681	0.804	0.132	0	LEC	TM4SF1
112	SOX47	0	2.08324182562505	0.773	0.322	0	LEC	SOX4

112	RHOJ1	0	2.07868858595974	0.594	0.032	0	LEC	RHOJ
112	CD910	0	2.04339218502565	0.857	0.331	0	LEC	CD9
112	EFEMP12	0	1.98230437550914	0.57	0.078	0	LEC	EFEMP1
112	LMO22	0	1.980089660767	0.601	0.06	0	LEC	LMO2
112	ADIRF6	0	1.9264530615374	0.802	0.284	0	LEC	ADIRF
112	EGFL71	0	1.92060299274053	0.562	0.03	0	LEC	EGFL7
112	ARL4A4	0	1.91727137724561	0.739	0.275	0	LEC	ARL4A
112	PDPN1	0	1.8918882485993	0.574	0.04	0	LEC	PDPN
112	FABP55	0	1.87896251118213	0.746	0.296	0	LEC	FABP5
113	CRIP26	0	1.8691208938254	0.7	0.149	0	LEC	CRIP2
113	NR2F1	0	1.84676632696861	0.493	0.033	0	LEC	NR2F1
113	CALCRL1	0	1.82495878795606	0.572	0.058	0	LEC	CALCRL
113	APP7	0	1.80304477858038	0.776	0.256	0	LEC	APP
113	ELK31	0	1.76950691017188	0.671	0.177	0	LEC	ELK3
113	CD596	0	1.75206244475769	0.837	0.334	0	LEC	CD59
113	ARHGAP294	0	1.74691804511318	0.64	0.091	0	LEC	ARHGAP29
113	CNKSR31	0	1.73684189624464	0.51	0.042	0	LEC	CNKSR3
113	CAV19	0	1.67536244228588	0.755	0.24	0	LEC	CAV1
113	TIMP37	0	1.64491515381195	0.755	0.218	0	LEC	TIMP3
114	HYAL21	0	1.64438715528173	0.445	0.028	0	LEC	HYAL2
114	MTUS14	0	1.63605646882241	0.546	0.08	0	LEC	MTUS1
114	NR2F24	0	1.62187801694444	0.649	0.127	0	LEC	NR2F2
114	LAMA46	0	1.61183117313065	0.61	0.13	0	LEC	LAMA4
114	S100A106	0	1.61111440210564	0.974	0.806	0	LEC	S100A10
114	TBX11	0	1.55376836950295	0.445	0.015	0	LEC	TBX1
114	SMAD1	0	1.55336795886331	0.459	0.025	0	LEC	SMAD1
114	FN15	0	1.52889140318767	0.559	0.137	0	LEC	FN1
114	ACKR32	0	1.48471009582679	0.385	0.065	0	LEC	ACKR3
114	HSPB112	3.7883	1.75381148492857	0.974	0.73	1.2500068	LEC	HSPB1
115	PDK45	1.4722	2.17617633715224	0.456	0.122	4.8577650	LEC	PDK4
115	IGKC1	0	8.76118723418525	0.865	0.236	0	PlasmaC	IGKC
115	IGLC2	0	8.37959437410917	0.62	0.135	0	PlasmaC	IGLC2
115	IGLC3	0	8.16711270296258	0.464	0.072	0	PlasmaC	IGLC3
115	IGHG1	0	7.71973041456078	0.7	0.065	0	PlasmaC	IGHG1

115	IGHA1	0	7.33085802090256	0.515	0.056	0	PlasmaC	IGHA1	
115	IGHG4	0	6.78729134194579	0.706	0.039	0	PlasmaC	IGHG4	
115	IGHG2	0	6.53206901330586	0.45	0.021	0	PlasmaC	IGHG2	
115	IGHG3	0	6.30303281837895	0.689	0.04	0	PlasmaC	IGHG3	
115	JCHAIN	0	5.82712633149606	0.556	0.013	0	PlasmaC	JCHAIN	
116	IGHGP	0	5.67063974848476	0.415	0.017	0	PlasmaC	IGHGP	
116	IGHA2	0	4.73443932384808	0.295	0.007	0	PlasmaC	IGHA2	
116	MZB1	0	3.64823133441335	0.919	0.011	0	PlasmaC	MZB1	
116	SSR42	0	3.24592073228867	0.953	0.574	0	PlasmaC	SSR4	
116	HERPUD14	0	2.79692001468341	0.968	0.585	0	PlasmaC	HERPUD1	
116	DERL3	0	2.62466438044901	0.826	0.005	0	PlasmaC	DERL3	
116	CD79A1	0	2.23674573163142	0.743	0.023	0	PlasmaC	CD79A	
116	SEC11C3	0	2.03258706921937	0.861	0.2	0	PlasmaC	SEC11C	
116	CYBA5	0	1.91419985236179	0.932	0.576	0	PlasmaC	CYBA	
116	FKBP11	0	1.88653837443592	0.794	0.132	0	PlasmaC	FKBP11	
117	IGLV3-1	0	1.81946238369264	0.296	0.001	0	PlasmaC	IGLV3-1	
117	PRDX44	0	1.71045767077824	0.764	0.136	0	PlasmaC	PRDX4	
117	DNAAF1	0	1.56370367119108	0.274	0.006	0	PlasmaC	DNAAF1	
117	FKBP25	0	1.39014317689358	0.81	0.264	0	PlasmaC	FKBP2	
117	TRIB15	0	1.37675191985869	0.647	0.146	0	PlasmaC	TRIB1	
117	ITM2C2	0	1.33908727207507	0.624	0.118	0	PlasmaC	ITM2C	
117	MIR155HG3	0	1.33150862292946	0.555	0.134	0	PlasmaC	MIR155HG	
117	RAB301	0	1.31713882624924	0.599	0.072	0	PlasmaC	RAB30	
117	SLAMF71	0	1.17097179815577	0.606	0.053	0	PlasmaC	SLAMF7	
117	SPCS15	0	1.13753897574175	0.817	0.347	0	PlasmaC	SPCS1	
118	RASGRP32	0	1.0965802596283	0.391	0.08	0	PlasmaC	RASGRP3	
118	FCRL5	0	1.07768453034523	0.543	0.007	0	PlasmaC	FCRL5	
118	CD271	0	1.00364580236391	0.566	0.076	0	PlasmaC	CD27	
118	ERLEC11	0	0.967524041788438	0.612	0.126	0	PlasmaC	ERLEC1	
118	JSRP1	0	0.96392107509669	0.376	0.004	0	PlasmaC	JSRP1	
118	NUCB23	0	0.935051584801858	0.633	0.137	0	PlasmaC	NUCB2	
118	SDF2L13	0	0.895415297845267	0.658	0.203	0	PlasmaC	SDF2L1	
118	TNFRSF17	0	0.858283379744261	0.376	0.002	0	PlasmaC	TNFRSF17	
118	BHLHE41	0	0.842548848329289	0.425	0.043	0	PlasmaC	BHLHE41	

118	HSP90B16	3.4979	1.44233305470194	0.917	0.648	1.1541854	PlasmaC	HSP90B1
119	H1FX4	2.5628	1.40557724356424	0.73	0.312	8.4564818	PlasmaC	H1FX
119	SPCS22	2.3611	0.919458092021302	0.836	0.419	7.7908236	PlasmaC	SPCS2
119	ANKRD284	3.8332	0.838958998506439	0.752	0.29	1.2648341	PlasmaC	ANKRD28
119	XBP13	1.0359	1.30193681144399	0.865	0.463	3.4183818	PlasmaC	XBP1
119	CYTOR5	1.8915	1.21734086760486	0.751	0.357	6.2412957	PlasmaC	CYTOR
119	ANKRD371	1.2816	0.977865812438053	0.489	0.169	4.2287965	PlasmaC	ANKRD37
119	ERN 14.00	1.1534	1.01624908759012	0.513	0.196	3.8059341	PlasmaC	ERN 1.00
119	PELI15	9.3193	0.899805205394868	0.624	0.267	3.0750043	PlasmaC	PELI1
119	THAP22	3.0902	1.15760580667888	0.372	0.124	1.0196524	PlasmaC	THAP2
119	HSPA69	1.2315	1.20072228786725	0.403	0.294	4.0635546	PlasmaC	HSPA6
120	CRIP16	1.2532	0.910805115530133	0.586	0.428	4.1353633	PlasmaC	CRIP1
120	COCH1	0	5.51705057464824	0.663	0.026	0	PTGDS+	COCH
120	PTGDS4	0	3.64887914485224	0.875	0.133	0	PTGDS+	PTGDS
120	ASPN1	0	3.34173438037836	0.758	0.041	0	PTGDS+	ASPN
120	LUM4	0	3.03692477873514	0.858	0.168	0	PTGDS+	LUM
120	IGFBP56	0	2.92530479024869	0.782	0.178	0	PTGDS+	IGFBP5
120	OGN2	0	2.92501927596324	0.696	0.053	0	PTGDS+	OGN
120	COL1A24	0	2.6923298080604	0.987	0.281	0	PTGDS+	COL1A2
120	CTSK3	0	2.66057921104993	0.885	0.136	0	PTGDS+	CTSK
120	MFAP43	0	2.5527728817423	0.746	0.13	0	PTGDS+	MFAP4
121	TCF49	0	2.49020953628413	0.9	0.26	0	PTGDS+	TCF4
121	COL1A13	0	2.44216124221075	0.981	0.267	0	PTGDS+	COL1A1
121	DPT3	0	2.4000298346685	0.674	0.098	0	PTGDS+	DPT
121	HTRA15	0	2.36259758494527	0.792	0.133	0	PTGDS+	HTRA1
121	CYR618	0	2.34630601942005	0.803	0.227	0	PTGDS+	CYR61
121	POSTN1	0	2.30981708034088	0.398	0.071	0	PTGDS+	POSTN
121	ID38	0	2.29296528686013	0.81	0.336	0	PTGDS+	ID3
121	CRABP1	0	2.21060205406567	0.537	0.017	0	PTGDS+	CRABP1
121	TNN	0	2.16337580524533	0.526	0.005	0	PTGDS+	TNN
121	SPARCL15	0	2.14448051798028	0.838	0.2	0	PTGDS+	SPARCL1
122	THY14	0	2.08980190851712	0.715	0.105	0	PTGDS+	THY1
122	MEG34	0	2.07998199313156	0.774	0.154	0	PTGDS+	MEG3
122	FIBIN	0	2.03968501591929	0.546	0.026	0	PTGDS+	FIBIN

122	FN16	0	2.01259680179412	0.724	0.136	0	PTGDS+	FN1
122	PRELP3	0	2.00705645206851	0.641	0.055	0	PTGDS+	PRELP
122	SOD36	0	2.00206450914312	0.844	0.197	0	PTGDS+	SOD3
122	DCN4	0	1.98375544325523	0.992	0.323	0	PTGDS+	DCN
122	PCOLCE6	0	1.9589511779945	0.764	0.154	0	PTGDS+	PCOLCE
122	CRABP24	0	1.9509528643101	0.634	0.094	0	PTGDS+	CRABP2
122	TWIST14	0	1.93443140022853	0.654	0.155	0	PTGDS+	TWIST1
123	MXRA51	0	1.90733518451433	0.579	0.05	0	PTGDS+	MXRA5
123	DKK 21.00	0	1.902333045391	0.591	0.029	0	PTGDS+	DKK 2.00
123	PGRMC16	0	1.86915185235	0.718	0.195	0	PTGDS+	PGRMC1
123	EMID1	0	1.84613909799008	0.543	0.02	0	PTGDS+	EMID1
123	ZBTB207	0	1.83437170307278	0.745	0.32	0	PTGDS+	ZBTB20
123	COL6A15	0	1.8332120953268	0.879	0.234	0	PTGDS+	COL6A1
123	TNMD	0	1.77826454370671	0.38	0.01	0	PTGDS+	TNMD
123	CD911	0	1.76395015575103	0.857	0.331	0	PTGDS+	CD9
123	LTBP44	0	1.76357026803286	0.657	0.147	0	PTGDS+	LTBP4
123	COL3A14	0	1.74639168760714	0.803	0.221	0	PTGDS+	COL3A1
124	PTN3	0	1.73645568108167	0.612	0.087	0	PTGDS+	PTN
124	FMOD2	0	1.70242516571441	0.548	0.051	0	PTGDS+	FMOD
124	SFRP12	0	1.68311805649694	0.504	0.045	0	PTGDS+	SFRP1
124	PLXDC1	0	1.67196603336736	0.502	0.05	0	PTGDS+	PLXDC1
124	COL6A34	0	1.63882843650495	0.676	0.149	0	PTGDS+	COL6A3
124	COL6A24	0	1.610887788793	0.922	0.272	0	PTGDS+	COL6A2
124	EID19	0	1.60642067273903	0.844	0.403	0	PTGDS+	EID1
124	APCDD11	0	1.60489088314889	0.498	0.056	0	PTGDS+	APCDD1
124	PMP229	0	1.59935868861568	0.792	0.255	0	PTGDS+	PMP22
124	MXRA84	0	1.5979467202577	0.612	0.092	0	PTGDS+	MXRA8
125	SEPP12	1.851	1.80326546989473	0.29	0.075	6.1081433	PTGDS+	SEPP1
125	CCL17	0	3.89657142078375	0.478	0.012	0	MigDC	CCL17
125	CCL221	0	3.73181797452988	0.665	0.019	0	MigDC	CCL22
125	CCR72	0	3.22990018673112	0.868	0.103	0	MigDC	CCR7
125	BIRC36	0	3.12427514921844	0.992	0.566	0	MigDC	BIRC3
125	G0S26	0	3.04294169778411	0.756	0.207	0	MigDC	G0S2
125	TXN7	0	3.04204961076576	0.977	0.667	0	MigDC	TXN

125	CD836	0	2.98882819112485	0.964	0.265	0	MigDC	CD83
125	GPR1572	0	2.69599700195276	0.798	0.058	0	MigDC	GPR157
125	DAPP13	0	2.68829305995615	0.904	0.105	0	MigDC	DAPP1
126	MARCKSL1	0	2.59464580509343	0.915	0.255	0	MigDC	MARCKSL1
126	FSCN15	0	2.51368353435081	0.847	0.121	0	MigDC	FSCN1
126	HLA-DPB14	0	2.51361613595539	0.968	0.42	0	MigDC	HLA-DPB1
126	POGLUT1	0	2.47188258729292	0.827	0.052	0	MigDC	POGLUT1
126	BASP15	0	2.46816533111286	0.907	0.225	0	MigDC	BASP1
126	IDO11	0	2.42802238884829	0.489	0.015	0	MigDC	IDO1
126	LAMP3	0	2.42643576812917	0.847	0.015	0	MigDC	LAMP3
126	CSF2RA4	0	2.42188078491225	0.85	0.08	0	MigDC	CSF2RA
126	MARCKS10	0	2.29560980218616	0.951	0.355	0	MigDC	MARCKS
126	RASSF41	0	2.27861514864376	0.788	0.061	0	MigDC	RASSF4
127	LGALS23	0	2.20928866633571	0.675	0.065	0	MigDC	LGALS2
127	NUB11	0	2.19009163208445	0.865	0.197	0	MigDC	NUB1
127	NRP28	0	2.02075040541824	0.871	0.143	0	MigDC	NRP2
127	TNFAIP27	0	1.96352490704491	0.819	0.172	0	MigDC	TNFAIP2
127	CD1B	0	1.94812892462276	0.41	0.015	0	MigDC	CD1B
127	CD274	0	1.89107722684992	0.646	0.046	0	MigDC	CD274
127	TYROBP5	0	1.86604441201638	0.885	0.182	0	MigDC	TYROBP
127	HLA-DQA14	0	1.8359298953022	0.926	0.264	0	MigDC	HLA-DQA1
127	LSP13	0	1.77982638882061	0.848	0.247	0	MigDC	LSP1
127	IRF44	0	1.77976767025285	0.73	0.102	0	MigDC	IRF4
128	CERS6	0	1.7380371261724	0.751	0.058	0	MigDC	CERS6
128	KDM2B	0	1.71629279851656	0.728	0.082	0	MigDC	KDM2B
128	TNFSF13B3	0	1.70324127893735	0.583	0.078	0	MigDC	TNFSF13B
128	ALDH25	0	1.70087562400205	0.754	0.121	0	MigDC	ALDH2
128	ETV34	0	1.6816789059037	0.806	0.216	0	MigDC	ETV3
128	CD404	0	1.61460725817192	0.645	0.108	0	MigDC	CD40
128	FAM49A6	0	1.61305981455844	0.73	0.123	0	MigDC	FAM49A
128	TMEM176A	0	1.60499301896074	0.625	0.084	0	MigDC	TMEM176A
128	TBC1D41	0	1.58541182687013	0.716	0.11	0	MigDC	TBC1D4
128	LYZ4	4.075	1.76948989871548	0.774	0.196	1.3446361	MigDC	LYZ
129	CTSZ4	2.367	1.88805735748567	0.888	0.322	7.8114507	MigDC	CTSZ

129	HLA-DPA14	2.7040	1.83829498933293	0.959	0.411	8.9222107	MigDC	HLA-DPA1
129	BCL2A15	2.9641	1.6873164313941	0.748	0.2	9.7804683	MigDC	BCL2A1
129	KIF2A2	1.6473	1.6494112467098	0.721	0.202	5.4357147	MigDC	KIF2A
129	CD745	8.8474	2.00513846557985	0.989	0.592	2.9193005	MigDC	CD74
129	DUSP53	1.1749	2.06086207832019	0.877	0.346	3.8767045	MigDC	DUSP5
129	HLA-DRA5	5.9076	1.9378522840169	0.983	0.499	1.9492953	MigDC	HLA-DRA
129	RGS17	1.0681	1.8168625313952	0.939	0.409	3.5244125	MigDC	RGS1
129	ID26	2.9709	2.00953206568041	0.956	0.552	9.8029536	MigDC	ID2
129	ACTB6	3.0046	1.68003135761616	0.986	0.918	9.9140955	MigDC	ACTB
130	IER55	1.2910	1.68652715013916	0.86	0.465	4.2598518	MigDC	IER5
130	IL8	0	5.07814716870938	0.62	0.041	0	IL8+ DC	IL8
130	IGLL5	0	3.89936374159999	0.383	0.015	0	IL8+ DC	IGLL5
130	IGJ	0	3.47830233076511	0.361	0.013	0	IL8+ DC	IGJ
130	RP11-1143G	0	2.73155873051111	0.476	0.012	0	IL8+ DC	RP11-1143G
130	HLA-DQB23	0	2.60797906948086	0.617	0.042	0	IL8+ DC	HLA-DQB2
130	FCER1A2	0	2.60660641475643	0.678	0.068	0	IL8+ DC	FCER1A
130	HLA-DQA25	0	2.3469264050843	0.622	0.097	0	IL8+ DC	HLA-DQA2
130	AGPAT9	0	2.20975393286655	0.495	0.011	0	IL8+ DC	AGPAT9
130	HLA-DQB15	0	2.20285201731227	0.907	0.322	0	IL8+ DC	HLA-DQB1
131	G0S27	0	2.18884250544599	0.856	0.207	0	IL8+ DC	G0S2
131	SELK2	0	2.17191816228512	0.583	0.082	0	IL8+ DC	SELK
131	LYZ5	0	2.1493594068883	0.874	0.195	0	IL8+ DC	LYZ
131	ATP5E3	0	2.13337702130384	0.626	0.1	0	IL8+ DC	ATP5E
131	IL1R23	0	2.10041900594542	0.709	0.089	0	IL8+ DC	IL1R2
131	CXCL34	0	2.06054812026274	0.87	0.223	0	IL8+ DC	CXCL3
131	C15orf485	0	1.97283664799045	0.72	0.142	0	IL8+ DC	C15orf48
131	S100A22	0	1.93565860030543	0.896	0.215	0	IL8+ DC	S100A2
131	HLA-DQA15	0	1.7915684162794	0.919	0.264	0	IL8+ DC	HLA-DQA1
131	GPX12	0	1.78653154510234	0.571	0.063	0	IL8+ DC	GPX1
132	TYROBP6	0	1.77696799923692	0.851	0.183	0	IL8+ DC	TYROBP
132	LST15	0	1.72183891149443	0.681	0.143	0	IL8+ DC	LST1
132	HLA-DMA6	0	1.68831065615701	0.741	0.191	0	IL8+ DC	HLA-DMA
132	AIF14	0	1.66061670866518	0.753	0.15	0	IL8+ DC	AIF1
132	CLEC10A3	0	1.60842342370983	0.551	0.066	0	IL8+ DC	CLEC10A

132	FCER1G5	0	1.60332572854772	0.812	0.164	0	IL8+ DC	FCER1G
132	KRT142	0	1.5764077632641	0.885	0.224	0	IL8+ DC	KRT14
132	LGALS24	0	1.4756541883585	0.525	0.066	0	IL8+ DC	LGALS2
132	ATP5L3	0	1.44157485158344	0.571	0.099	0	IL8+ DC	ATP5L
132	CD1C1	0	1.4210539289609	0.453	0.038	0	IL8+ DC	CD1C
133	SFN2	0	1.39074562785852	0.81	0.117	0	IL8+ DC	SFN
133	CD1E2	0	1.38726680453387	0.346	0.024	0	IL8+ DC	CD1E
133	MMP93	0	1.32213207888307	0.588	0.114	0	IL8+ DC	MMP9
133	ATP5G23	0	1.29969841542273	0.539	0.089	0	IL8+ DC	ATP5G2
133	HLA-DPB15	1.5990	2.11982327176099	0.943	0.42	5.2762196	IL8+ DC	HLA-DPB1
133	CST36	1.0054	2.46747323161587	0.979	0.519	3.3176162	IL8+ DC	CST3
133	HLA-DPA15	1.9888	1.93630530154163	0.94	0.411	6.5625730	IL8+ DC	HLA-DPA1
133	HLA-DRA6	2.6678	2.40899788048021	0.953	0.499	8.8029435	IL8+ DC	HLA-DRA
133	HLA-DRB15	1.5445	2.05228621081465	0.948	0.458	5.0965328	IL8+ DC	HLA-DRB1
133	GNB2L14	3.2813	1.34379779974233	0.629	0.125	1.0827241	IL8+ DC	GNB2L1
134	CD746	4.9790	2.3277676982778	0.957	0.592	1.6429010	IL8+ DC	CD74
134	CXCL26	1.2396	1.39882370934634	0.884	0.306	4.0902857	IL8+ DC	CXCL2
134	PLAUR6	1.3438	1.53729484037411	0.859	0.327	4.4341091	IL8+ DC	PLAUR
134	HLA-DRB56	2.8020	2.9537023467075	0.663	0.235	9.2457595	IL8+ DC	HLA-DRB5
134	ALDOA3	4.6242	1.33692234755727	0.58	0.148	1.5258062	IL8+ DC	ALDOA
134	FTH16	4.0115	1.46432108448052	1	0.984	1.3236519	IL8+ DC	FTH1
134	INSIG14	5.9380	2.25606935014473	0.842	0.387	1.9593201	IL8+ DC	INSIG1
134	FABP56	2.2641	1.41170856437029	0.767	0.298	7.4706419	IL8+ DC	FABP5
134	SAT17	1.7579	1.62441497658648	0.974	0.845	5.8005510	IL8+ DC	SAT1
134	SGK16	2.3687	1.31182746764935	0.758	0.291	7.8159878	IL8+ DC	SGK1
135	TMSB4X7	3.9970	1.55842254014516	0.998	0.973	1.3188582	IL8+ DC	TMSB4X
135	NRXN1	0	4.02236412858995	0.707	0.001	0	Neurona	NRXN1
135	CDH191	0	4.0001650365123	0.763	0.012	0	Neurona	CDH19
135	CRYAB7	0	3.77019196927296	0.94	0.242	0	Neurona	CRYAB
135	GPM6B3	0	3.57879909971573	0.817	0.102	0	Neurona	GPM6B
135	S100B3	0	3.48318109629984	0.635	0.046	0	Neurona	S100B
135	SCN7A	0	3.40307406072423	0.529	0.01	0	Neurona	SCN7A
135	PMP2210	0	3.26361477826963	0.859	0.257	0	Neurona	PMP22
135	MPZ	0	3.11595835946705	0.386	0.006	0	Neurona	MPZ

135	PLP11	0	3.05022979351115	0.6	0.023	0	Neurona	PLP1
136	ITGB82	0	2.7764355805904	0.481	0.036	0	Neurona	ITGB8
136	NTM	0	2.62084674849791	0.542	0.009	0	Neurona	NTM
136	MATN2	0	2.50026330300495	0.506	0.027	0	Neurona	MATN2
136	CADM11	0	2.47303106156721	0.523	0.038	0	Neurona	CADM1
136	HSPA12A	0	2.42422415810791	0.546	0.029	0	Neurona	HSPA12A
136	ANK3	0	2.40327628617998	0.442	0.043	0	Neurona	ANK3
136	PCSK21	0	2.35612119929135	0.376	0.007	0	Neurona	PCSK2
136	VWA1	0	2.3481672946559	0.51	0.02	0	Neurona	VWA1
136	XKR4	0	2.25665508906551	0.365	0.001	0	Neurona	XKR4
136	SEMA3B	0	2.19726988563412	0.436	0.011	0	Neurona	SEMA3B
137	GFRA3	0	2.14850859234138	0.432	0.001	0	Neurona	GFRA3
137	GAS73	0	2.1422386997773	0.515	0.079	0	Neurona	GAS7
137	SOX2	0	2.02058050109341	0.332	0.003	0	Neurona	SOX2
137	PLAT1	0	1.99337133058727	0.454	0.036	0	Neurona	PLAT
137	SEMA3C1	0	1.99089275798834	0.469	0.042	0	Neurona	SEMA3C
137	L1CAM	0	1.98312357100058	0.429	0.011	0	Neurona	L1CAM
137	PDLIM46	0	1.98258205520451	0.564	0.084	0	Neurona	PDLIM4
137	CHL1	0	1.89309254955798	0.299	0.014	0	Neurona	CHL1
137	FOXD3	0	1.8827038675684	0.324	0.003	0	Neurona	FOXD3
137	COL28A1	0	1.87445889570733	0.305	0.001	0	Neurona	COL28A1
138	PTPRZ1	0	1.87093921664889	0.373	0.012	0	Neurona	PTPRZ1
138	CNN39	8.5636	2.33125595355845	0.774	0.203	2.8256779	Neurona	CNN3
138	DST8	9.0901	2.54955269062491	0.793	0.222	2.9994017	Neurona	DST
138	COL18A17	4.8128	2.17739218301238	0.62	0.135	1.5880546	Neurona	COL18A1
138	ITGA64	1.1003	1.90788028955685	0.417	0.06	3.6306298	Neurona	ITGA6
138	CLIC49	8.9049	2.30455358453596	0.701	0.189	2.9382734	Neurona	CLIC4
138	TCIM3	2.1751	1.85473524701039	0.454	0.069	7.1771265	Neurona	TCIM
138	RCAN13	1.1805	2.46565437708094	0.494	0.088	3.8953028	Neurona	RCAN1
138	SPARC7	6.3090	2.37389065937943	0.83	0.273	2.0817328	Neurona	SPARC
138	NDRG23	5.0409	1.87923717539529	0.452	0.085	1.6633161	Neurona	NDRG2
139	CD913	8.0703	2.52281627632109	0.847	0.333	2.6629043	Neurona	CD9
139	GPR1551	2.4281	1.87562392980922	0.425	0.077	8.0118693	Neurona	GPR155
139	COL4A26	4.8089	1.77749931801543	0.492	0.108	1.5867483	Neurona	COL4A2

139	SPTBN19	1.0682	1.89051336561028	0.689	0.226	3.5249498	Neurona	SPTBN1
139	IGFBP57	9.1923	2.69795640215635	0.598	0.181	3.0331208	Neurona	IGFBP5
139	MARCKS12	1.5479	1.98300132857579	0.788	0.356	5.1074648	Neurona	MARCKS
139	PGM2L13	2.4228	1.88727425222232	0.465	0.128	7.9945508	Neurona	PGM2L1
139	PTN4	6.8819	1.84066229080155	0.384	0.09	2.2707643	Neurona	PTN
139	TUBA1A9	2.5281	1.81805787983802	0.757	0.497	8.3420204	Neurona	TUBA1A
139	PRNP4	1.4719	1.8622355938985	0.724	0.487	4.8569951	Neurona	PRNP
140	PMEPA16	2.8867	1.83629490433844	0.467	0.255	9.5249732	Neurona	PMEPA1
140	DES	0	6.1809828500975	0.97	0.011	0	SMC	DES
140	ACTG2	0	5.60040943954112	0.976	0.011	0	SMC	ACTG2
140	ACTA22	0	5.54210890225559	0.994	0.104	0	SMC	ACTA2
140	MYLK3	0	5.37952021653091	0.988	0.09	0	SMC	MYLK
140	MYH111	0	4.94939996468265	0.988	0.056	0	SMC	MYH11
140	CNN11	0	4.58356614863666	0.994	0.023	0	SMC	CNN1
140	PCP4	0	4.54565290580232	0.88	0.001	0	SMC	PCP4
140	CSRP15	0	4.16644408597815	0.988	0.125	0	SMC	CSRP1
140	PPP1R14A1	0	3.67997606176232	0.964	0.047	0	SMC	PPP1R14A
141	SELM3	0	3.62541419474664	0.843	0.077	0	SMC	SELM
141	ACTA1	0	3.37658633751743	0.614	0.004	0	SMC	ACTA1
141	SMTN	0	3.21730623988112	0.873	0.063	0	SMC	SMTN
141	NRN13	0	3.05328844608668	0.795	0.043	0	SMC	NRN1
141	SYNPO22	0	2.95589517545024	0.783	0.057	0	SMC	SYNPO2
141	PDLIM71	0	2.75161501588726	0.807	0.062	0	SMC	PDLIM7
141	RAMP14	0	2.68334887219594	0.831	0.06	0	SMC	RAMP1
141	LMOD12	0	2.68205042937632	0.861	0.04	0	SMC	LMOD1
141	PDLIM33	0	2.61020088790065	0.831	0.059	0	SMC	PDLIM3
141	NEXN	0	2.55111256329237	0.783	0.028	0	SMC	NEXN
142	PLN 1.00	0	2.32731066120179	0.765	0.025	0	SMC	PLN
142	SYNM2	0	2.26380424023465	0.669	0.044	0	SMC	SYNM
142	PRKCDBP1	0	2.25018529762212	0.699	0.048	0	SMC	PRKCDBP
142	ITGA8	0	2.14117224666801	0.651	0.013	0	SMC	ITGA8
142	MSRB32	2.9633	2.15522292773733	0.735	0.063	9.7777262	SMC	MSRB3
142	KANK24	7.2213	2.25898130923608	0.765	0.07	2.3827661	SMC	KANK2
142	SEPW14	1.5152	2.46166901372801	0.735	0.064	4.9997802	SMC	SEPW1

142	TAGLN4	9.7598	5.5635682077539	0.994	0.152	3.2203675	SMC	TAGLN
142	FHL15	3.7266	2.91819174491353	0.892	0.108	1.2296541	SMC	FHL1
142	TPM22	4.5451	5.51753737769328	1	0.162	1.4997140	SMC	TPM2
143	PTRF3	2.9934	2.26399427548069	0.711	0.065	9.8771126	SMC	PTRF
143	MYL94	4.2985	5.28632992161244	0.994	0.165	1.4183429	SMC	MYL9
143	CKB9	4.9380	2.97978677071222	0.91	0.144	1.6293516	SMC	CKB
143	PALLD6	1.8379	2.48923995039841	0.855	0.121	6.0645370	SMC	PALLD
143	TPM16	8.5687	4.16162141881816	1	0.2	2.8273461	SMC	TPM1
143	MFAP44	1.4917	2.36339859987845	0.861	0.134	4.9220920	SMC	MFAP4
143	SLMAP1	1.7067	2.39527454739305	0.759	0.111	5.6315673	SMC	SLMAP
143	GAS65	4.3154	2.10546188315044	0.789	0.137	1.4239414	SMC	GAS6
143	ALDOA4	4.7557	2.56578588454734	0.795	0.149	1.5692209	SMC	ALDOA
143	CAV111	3.3910	2.80138225411052	0.982	0.243	1.1189236	SMC	CAV1
144	CD1518	8.4660	2.23080099827713	0.819	0.159	2.7934496	SMC	CD151
144	ACTN19	3.4550	2.27585421728358	0.825	0.179	1.1400331	SMC	ACTN1
144	CALD19	1.7986	2.80463462494216	0.982	0.313	5.9348997	SMC	CALD1
144	DSTN10	3.4632	3.08050770617349	1	0.469	1.1427335	SMC	DSTN
144	ADIRF8	1.6052	2.36614336367077	0.97	0.287	5.2965506	SMC	ADIRF
144	LPP2	1.6501	2.33157012512484	0.861	0.248	5.4447200	SMC	LPP
144	NDUFA410	7.6321	2.48535398367769	1	0.565	2.5182883	SMC	NDUFA4
144	FLNA5	2.1565	2.58920300796982	0.952	0.445	7.1158987	SMC	FLNA
144	MYL67	6.5487	2.42711006059938	1	0.887	2.1608094	SMC	MYL6
144	EID111	1.1530	2.24792309145167	0.946	0.405	3.8046071	SMC	EID1
145	ACTB8	9.5773	2.3571971116514	1	0.918	3.1601334	SMC	ACTB
145	ACTA11	0	8.18547545646324	0.969	0.005	0	Skeletal	ACTA1
145	TNNC2	0	7.38558923692975	0.875	0.01	0	Skeletal	TNNC2
145	MYLPF	0	7.05905205042728	0.875	0.014	0	Skeletal	MYLPF
145	MB	0	7.05217681541108	0.969	0.004	0	Skeletal	MB
145	MYL1	0	7.04220814026433	0.969	0.002	0	Skeletal	MYL1
145	CKM	0	7.02109465746987	0.938	0.002	0	Skeletal	CKM
145	TCAP	0	6.95341590386022	0.938	0.006	0	Skeletal	TCAP
145	TNNI2	0	6.93415396684556	0.875	0.009	0	Skeletal	TNNI2
145	TNNT3	0	6.74253382219968	0.844	0.011	0	Skeletal	TNNT3
146	CSRP3	0	6.43417083941691	0.938	0.001	0	Skeletal	CSRP3

146	ENO3	0	6.12097572732393	1	0.007	0	Skeletal	ENO3
146	COX6A2	0	6.09714754726781	0.969	0.001	0	Skeletal	COX6A2
146	DES1	0	5.9965124307846	1	0.012	0	Skeletal	DES
146	MYL2	0	5.46419916570855	0.344	0.001	0	Skeletal	MYL2
146	ANKRD1	0	5.42331525270779	0.812	0.001	0	Skeletal	ANKRD1
146	SLN	0	5.24914562303869	0.906	0.002	0	Skeletal	SLN
146	MYH2	0	5.2387067463363	0.781	0.001	0	Skeletal	MYH2
146	PGAM21	0	4.47180994547504	0.875	0.005	0	Skeletal	PGAM2
146	NEB	0	4.41805123393173	0.75	0.005	0	Skeletal	NEB
147	ACTC1	0	4.40901395068165	0.688	0.002	0	Skeletal	ACTC1
147	KLHL41	0	4.31432034036215	0.781	0.004	0	Skeletal	KLHL41
147	MYOZ1	0	4.18769014501686	0.844	0.003	0	Skeletal	MYOZ1
147	NRAP	0	4.08922278227946	0.812	0	0	Skeletal	NRAP
147	XIRP2	0	4.02802623856966	0.562	0	0	Skeletal	XIRP2
147	PPP1R27	0	3.96197530655106	0.75	0.001	0	Skeletal	PPP1R27
147	MYBPC1	0	3.91783188218555	0.812	0.001	0	Skeletal	MYBPC1
147	SMPX	0	3.90122774051847	0.781	0	0	Skeletal	SMPX
147	XIRP1	0	3.89559093297076	0.562	0.003	0	Skeletal	XIRP1
147	ACTN2	0	3.80561128354285	0.781	0.003	0	Skeletal	ACTN2
148	CA3	0	3.66900574244842	0.438	0.002	0	Skeletal	CA3
148	EEF1A2	0	3.65018989055063	0.781	0.008	0	Skeletal	EEF1A2
148	MYOT1	0	3.44383286233556	0.75	0.001	0	Skeletal	MYOT
148	ATP2A1	0	3.40614175308828	0.625	0.001	0	Skeletal	ATP2A1
148	TNNT1	3.0162	5.13044791062127	0.469	0.007	9.9522906	Skeletal	TNNT1
148	CMYA5	1.2790	4.13847119126757	0.719	0.018	4.2202978	Skeletal	CMYA5
148	S100A17	8.6000	4.58100058140842	0.875	0.027	2.8376742	Skeletal	S100A1
148	TNNC1	1.1095	4.7794995614708	0.344	0.006	3.6611327	Skeletal	TNNC1
148	HSPB61	1.8601	3.8918474881019	0.844	0.038	6.1379104	Skeletal	HSPB6
148	PDLIM34	1.1724	3.7681698956666	0.781	0.06	3.8687488	Skeletal	PDLIM3
149	SLC25A43	5.8124	4.9739352753919	0.969	0.102	1.9178791	Skeletal	SLC25A4
149	COX7A110	1.3480	4.78923841159156	0.938	0.124	4.4479483	Skeletal	COX7A1
149	TTN2	7.5145	4.76406270003762	0.844	0.097	2.4794941	Skeletal	TTN
149	FHL16	9.9205	4.43893375967196	0.875	0.109	3.2733950	Skeletal	FHL1
149	TPM23	7.1916	5.62275034810634	1	0.162	2.3729558	Skeletal	TPM2

149	BIN11	2.5350	3.39292338655167	0.875	0.138	8.3646306	Skeletal	BIN1
149	RRAD3	1.0445	4.12300586229699	0.75	0.095	3.4466980	Skeletal	RRAD
149	LMCD12	6.7612	3.43850379348097	0.75	0.1	2.2309564	Skeletal	LMCD1
149	CRYAB9	5.1119	4.47690680015376	1	0.244	1.6867344	Skeletal	CRYAB
149	TPM17	1.5795	6.26709181297112	0.844	0.201	5.2119970	Skeletal	TPM1
150	CHCHD107	1.7663	3.55640626705163	0.875	0.258	5.8281268	Skeletal	CHCHD10

Supplementary Table 2 : List of custom genes for ISS

Gene full name	Gene Symbol	Gene ID	Aliases	ENSEMBL GeneID	NCBI Accession number
keratin 5	KRT5	3852	K5; CK5; DDD; DDD1; EBS2; KRT5A	ENSG00000186081	NG_008297.1
keratin 14	KRT14	3861	K14; NFJ; CK14; EBS3; EBS4	ENSG00000186847	NG_008624.1
caveolin 1	CAV1	857	CGL3; PPH3; BSCL3; LCCNS; VIP21; MSTP085	ENSG00000105974	NG_012051.1
caveolin 2	CAV2	858	CAV	ENSG00000105971	NG_029920.1
collagen type XVII alpha 1 chain	COL17A1	1308	ERED; BP180; BPA-2; BPAG2; LAD-1; BA16H23.2	ENSG00000065618	NG_007069.1
integrin subunit beta 1	ITGB1	3688	CD29; FNRB; MDF2; VLAB; GPIIA; MSK12; VLA-BETA	ENSG00000150093	NG_029012.1
CD46 molecule	CD46	4179	MCP; TLX; AHUS2; MIC10; TRA2.10	ENSG00000117335	NG_009296.1
delta like canonical Notch ligand 1	DLL1	28514	DL1; Delta; DELTA1; NEDBAS	ENSG00000198719	NG_027940.1
leucine rich repeats and immunoglobulin like domains 1	LRIG1	26018	LIG1; LIG-1	ENSG00000144749	not found
mitogen-activated protein kinase 1	MAPK1	5594	ERK; p38; p40; p41; ERK2; ERT1; NS13; ERK-2; MAPK2; PRKM1; PRKM2; P42MAPK; p41mapk; p42-MAPK	ENSG00000100030	NG_023054.2
keratin 10	KRT10	3858	BIE; EHK; K10; KPP; BCIE; CK10	ENSG00000186395	NG_008405.1
keratin 1	KRT1	3848	CK1, EHK, EHK1, EPPK, K1, KRT1A, NEPPK	ENSG00000167768	NG_008364.2

cyclin dependent kinase 1	CDK1	983	CDC2, CDC28A, P34CDC2	ENSG00000170312	NG_029877.1
proliferating cell nuclear antigen	PCNA	5111	ATLD2	ENSG00000132646	NG_047066.1
intercellular adhesion molecule 1	ICAM1	3383	BB2, CD54, P3.58	ENSG00000090339	NG_012083.1
C-C motif chemokine ligand 20	CCL20	6364	CKb4; LARC; ST38; MIP3A; Exodus; MIP-3a; SCYA20; MIP-3-alpha	ENSG00000115009	not found
lymphocyte antigen 6 family member D	LY6D	8581	E48; Ly-6D	ENSG00000167656	not found
keratin 17	KRT17	3872	PC; K17; PC2; 39.1; CK-17; PCHC1	ENSG00000128422	NG_008625.1
embigin	EMB	133418	GP70	ENSG00000170571	not found
microsomal glutathione S-transferase 1	MGST1	4257	MGST; GST12; MGST-I	ENSG00000008394	not found
Tyrosinase-related protein 1	TRYP1	7306	TRP; CAS2; CATB; GP75; OCA3; TRP1; TYRP; b-PROTEIN	ENSG00000107165	NG_011705.1
premelanosome protein	PMEL	6490	P1; SI; SIL; ME20; P100; SILV; ME20M; gp100; ME20-M; PMEL17; D12S53E	ENSG00000185664	NG_028086.1
formin 1	FMN1	342184	LD; FMN	ENSG00000248905	NG_042863.1
calpain 3	CAPN3	825	p94; CANP3; LGMD2; nCL-1; CANPL3; LGMD2A; LGMDD4; LGMDR1	ENSG00000092529	NG_008660.1
empty spiracles homeobox 2	EMX2	2018		ENSG00000170370	NG_013009.1
major facilitator superfamily domain containing 12	MFSD12	126321	PP3501; C19orf28	ENSG00000161091	not found

myelin protein zero	MPZ	4359	P0; CHM; DSS; MPP; CHN2; CMT1; CMT1B; CMT2I; CMT2J; CMT4E; CMTDI3; CMTDID; HMSNIB	ENSG00000158887	NG_008055.1
dickkopf WNT signaling pathway inhibitor 3	DKK3	27122	RIG; REIC	ENSG00000050165	not found
dermcidin	DCD	117159	PIF; AIDD; DSEP; HCAP; DCD-1	ENSG00000161634	not found
keratin 19	KRT19	3880	K19; CK19; K1CS	ENSG00000171345	NG_012285.1
sodium voltage-gated channel alpha subunit 7	SCN7A	6332	NaG; SCN6A; Nav2.1; Nav2.2	ENSG00000136546	NG_031928.1
myogenic factor 5	MYF5	4617	EORVA; bHLHc2	ENSG00000111049	not found
decorin	DCN	1634	CSCD; PG40; PGII; PGS2; DSPG2; SLRR1B	ENSG00000011465	NG_011672.1
platelet derived growth factor receptor alpha	PDGFRA	5156	CD140A; PDGFR2; PDGFR-2	ENSG00000134853	NG_009250.1
collagen type I alpha 1 chain	COL1A1	1277	OI1; OI2; OI3; OI4; EDSC; CAFYD; EDSARTH1	ENSG00000108821	NG_007400.1
fibroblast growth factor 7	FGF7	2252	KGF; HBGF-7	ENSG00000140285	NG_029159.1
collagen type VI alpha 5 chain	COL6A5	256076	VWA4; COL29A1	ENSG00000172752	NG_021424.1
prostaglandin D2 synthase	PTGDS	5730	PDS; PGD2; PGDS; LPGDS; PGDS2; L-PGDS	ENSG00000107317	not found
APC down-regulated 1	APCDD1	147495	HHS; HTS; B7323; HYPT1; DRAPC1; FP7019	ENSG00000154856	NG_027685.1
C-C motif chemokine ligand 2	CCL2	6347	HC11; MCAF; MCP1; MCP-1; SCYA2; GDCF-2; SMC-CF; HSMCR30	ENSG00000108691	NG_012123.1
apolipoprotein D	APOD	347	none	ENSG00000189058	not found

asporin	ASPN	54829	OS3; PLAP1; PLAP-1; SLRR1C	ENSG00000106819	NG_023430.2
WNT inhibitory factor 1	WIF1	11197	WIF-1	ENSG00000156076	not found
periostin	POSTN	10631	PN; OSF2; OSF-2; PDLPOSTN	ENSG00000133110	not found
secretory leukocyte peptidase inhibitor	SLPI	6590	ALP; MPI; ALK1; BLPI; HUSI; WAP4; WFDC4; HUSI-I	ENSG00000124107	NG_028137.1
cellular communication network factor 5	CCN5	8839	CT58; WISP2; CTGF-L	ENSG00000064205	not found
microfibril associated protein 5	MFAP5	8076	AAT9; MP25; MAGP2; MAGP-2; MFAP-5	ENSG00000197614	not found
prostaglandin-endoperoxide synthase	PTGS2	5743	COX2; COX-2; PHS-2; PGG/HS; PGHS-2; hCox-2; GRIPGHS	ENSG00000073756	NG_028206.2
HIC ZBTB transcriptional repressor 1	HIC1	3090	hic-1; ZBTB29; ZNF901	ENSG00000177374	NG_027689.1
secreted frizzled related protein 1	SFRP1	6422	FRP; FRP1; FrzA; FRP-1; SARP2	ENSG00000104332	not found
platelet and endothelial cell adhesion molecule 1	PECAM1	5175	CD31; PECA1; GPIIA'; PECAM-1; endoCAM; CD31/EndoCAM	ENSG00000261371	NG_047009.1
selectin E	SELE	6401	ELAM; ESEL; CD62E; ELAM1; LECAM2	ENSG00000007908	NG_012124.1
claudin 5	CLDN5	7122	AWAL; BEC1; TMVCF; TMDVCF; CPETRL1	ENSG00000184113	not found
fms related receptor tyrosine kinase 1	FLT1	2321	FLT; FLT-1; VEGFR1; VEGFR-1	ENSG00000102755	NG_012003.1
hes related family bHLH transcription factor with YRPW motif 1	HEY1	23462	CHF2; OAF1; HERP2; HESR1; HRT-1; NERP2; hHRT1; BHLHb31	ENSG00000164683	not found
CD93 molecule	CD93	22918	C1QR1; C1qRP; CDw93;	ENSG00000125810	not found

			ECSM3; MXRA4; C1qR(P); dJ737E23.1		
endomucin	EMCN	51705	EMCN2; MUC14	ENSG00000164035	not found
interferon alpha inducible protein 27	IFI27	3429	P27; ISG12; FAM14D; ISG12A	ENSG00000165949	not found
atypical chemokine receptor 1 (Duffy blood group)	ACKR1	2532	FY; Dfy; GPD; DARC; GpFy; CCBP1; CD234; WBCQ1; DARC/ACKR1	ENSG00000213088	NG_011626.3
synuclein gamma	SNCG	6623	SR; BCSG1	ENSG00000173267	NG_008783.1
lymphatic vessel endothelial hyaluronan receptor 1	LYVE1	10894	HAR; XLKD1; LYVE-1; CRSBP-1	ENSG00000133800	not found
prospero homeobox 1	PROX1	5629	none	ENSG00000117707	not found
C-C motif chemokine ligand 21	CCL21	6366	ECL; SLC; CKb9; TCA4; 6Ckine; SCYA21	ENSG00000137077	not found
caveolae associated protein 2	CAVIN2	8436	SDR; SDPR; PS-p68; cavin-2	ENSG00000168497	not found
multimerin 1	MMRN1	22915	ECM; MMRN; GPIa*; EMILIN4	ENSG00000138722	NG_032895.2
actin alpha 2, smooth muscle	ACTA2	59	ACTSA	ENSG00000107796	NG_011541.1
transgelin	TAGLN	6876	SM22; SMCC; TAGLN1; WS3-10; SM22-alpha	ENSG00000149591	not found
RERG like	RERGL	79785	none	ENSG00000111404	NG_052618.1
myosin heavy chain 11	MYH11	4629	AAT4, FAA4, SMHC, SMMHC	ENSG00000133392	NG_009299.1
metalloreductase	STEAP4	79689	TIARP; STAMP2; SchLAH; TNFAIP9	ENSG00000127954	NG_028313.1
regulator of G protein signaling 5	RGS5	8490	MST092; MST106; MST129; MSTP032; MSTP092; MSTP106; MSTP129	ENSG00000143248	NG_027731.2

desmin	DES	1674	CSM1; CSM2; CDCD3; LGMD1D; LGMD1E; LGMD2R	ENSG00000175084	NG_008043.1
actin gamma 2, smooth muscle	ACTG2	72	ACT; ACTE; VSCM; ACTA3; ACTL3; ACTSG	ENSG00000163017	NG_034140.1
T cell immunoreceptor with Ig and ITIM domains	TIGIT	201633	VSIG9; VSTM3; WUCAM	ENSG00000181847	not found
SPARC (osteonectin), cwcv and kazal like domains proteoglycan 2	SPOCK2	9806	testican-2	ENSG00000107742	not found
C-type lectin domain family 2 member D	CLEC2D	29121	CLAX; LLT1; OCIL	ENSG00000069493	not found
cathepsin W	CTSW	1521	LYPN	ENSG00000172543	not found
RUNX family transcription factor 3	RUNX3	864	AML2; CBFA3; PEBP2aC	ENSG00000020633	not found
serpin family B member 2	SERPINB2	5055	PAI; PAI2; PAI-2; PLANH2; HsT1201	ENSG00000197632	not found
twist family bHLH transcription factor 1	TWIST1	7291	CRS; CSO; SCS; ACS3; CRS1; BPES2; BPES3; SWCOS; TWIST; bHLHa38	ENSG00000122691	NG_008114.2
SRY-box transcription factor 10	SOX10	6663	DOM; WS4; PCWH; WS2E; WS4C	ENSG00000100146	NG_007948.1

Supplementary Table 3: List of predefined Immune General gene panel

Gene full name	Gene symbol	HGNC ID	Alias	ENSEMBL GeneID human	Annotation
protein tyrosine phosphatase receptor type C	PTPRC	HGNC: 9666	B220;CD45R;Cd45;L-CA;Ly-5;Lyt-4;T200;loc;B220;CD45;CD45R;GP180;L-CA;LCA;LY5;T200	ENSG00000081237	hematopoietic lineage
CD34 molecule	CD34	HGNC: 1662	AU040960	ENSG00000174059	hematopoietic stem cells and endothelial cells
CD40 molecule	CD40	HGNC: 11919	AI326936;Bp50;GP39;HIGM1;IGM;IMD3;T-BAM;TRAP;Tnfrsf5;p50;Bp50;CDW40;TNFRSF5;p50	ENSG00000101017	antigen presenting cells (APC)
CD80 molecule	CD80	HGNC: 1700	B71;Cd28l;Ly-53;Ly53;MIC17;TSA1;B7;B7-1;B7.1;BB1;CD28LG;CD28LG1;LAB7	ENSG00000121594	antigen presenting cells (APC)
Fc receptor, IgG, high affinity I			AI323638;AV092959;CD64;FcgammaRI;IGGHAFC		macrophages and monocytes
Fc fragment of IgG receptor Ia	FCGR1A	HGNC: 3613	CD64;CD64A;FCRI;IGFR1	ENSG00000150337	macrophages and monocytes
S100 calcium binding protein A8	S100A8	HGNC: 10498	60B8Ag;AI323541;B8Ag;CFAg;CP-10;Caga;MRP8;p8;60B8AG;CAGA;CFAG;CGLA;CP-10;L1Ag;MA387;MIF;MRP8;NIF;P8	ENSG00000143546	monocytes
CD14 molecule	CD14	HGNC: 1628		ENSG00000170458	CD14+ monocytes
Fc receptor, IgG, low affinity III			CD16		CD16+ monocytes
Fc fragment of IgG receptor IIIa	FCGR3A	HGNC: 3619	CD16;CD16A;FCG3;FCGR3;FCGRIII;FCR-10;FCRIII;FCRIIIA;IGFR3;IMD20	ENSG00000203747	CD16+ monocytes

membrane spanning 4-domains A2	MS4A2	HGNC: 7316	FcRB;Fce1b;Fcer1b;Fcrbeta;Ms4a1;fcERI;APY;ATOPY;FCER1B;FCERI;IGEL;IGER;IGHER;MS4A1	ENSG00000149534	mast cells
tryptase alpha/beta 1	TPSAB1	HGNC: 12019	MMCP-7;Mcp-7;Mcp7;Mcpt7;TPS1;TPS2;TPSB1;TPSB2;Tryptase-2	ENSG00000172236	mast cells
apolipoprotein E	APOE	HGNC: 613	AI255918;Apo-E;AD2;APO-E;ApoE4;LDLCQ5;LPG	ENSG00000130203	macrophages
complement C1q A chain	C1QA	HGNC: 1241	AI255395;Adic;C1q	ENSG00000173372	macrophages
complement C1q B chain	C1QB	HGNC: 1242	Adia	ENSG00000173369	macrophages
CD163 molecule	CD163	HGNC: 1631	CD163v2;CD163v3;M130;MM130;SCARI1	ENSG00000177575	macrophages
CD68 molecule	CD68	HGNC: 1693	Lamp4;Scard1;gp110;GP110;LAMP4;SCARD1	ENSG00000129226	macrophages
folate receptor beta	FOLR2	HGNC: 3793	FBP2;FR-P3;FR-beta;Folbp-2;Folbp2;BETA-HFR;FBP;FBP/PL-1;FOLR1;FR-BETA;FR-P3;FRbeta	ENSG00000165457	resident macrophages
CEA cell adhesion molecule 8	CEACAM8	HGNC: 1820	CD66b;CD67;CGM6;NCA-95	ENSG00000124469	granulocytes
fucosyltransferase 4	FUT4	HGNC: 4015	AI451562;CD15;FAL;FucT-IV;LeX;SSEA-1;Ssea1;CD15;ELFT;FCT3A;FUC-TIV;FUTIV;LeX;SSEA-1	ENSG00000196371	neutrophils and eosinophils
interleukin 3 receptor subunit alpha	IL3RA	08385	CD123;CDw123;SUT-1;CD123;IL3R;IL3RAY;IL3RX;IL3RY;hIL-3Ra	430000	basophils and plasmacytoid dendritic cells (pDC)
CD209 molecule	CD209	HGNC: 1641	CDSIGN;CLEC4L;DC-SIGN;DC-SIGN1	ENSG00000090659	dendritic cells (DC)
CD209a antigen			CD209;CDSIGN;CIRE;DC-SIGN;DC-SIGN1;Dcsign;SIGN-R1;SIGNR5		dendritic cells (DC)
integrin subunit alpha X	ITGAX	HGNC: 6152	AI449405;Cd11c;Cr4;N418;CD11C;SLEB6	ENSG00000140678	dendritic cells (DC)
C-type lectin domain containing 9A	CLEC9A	HGNC: 26705	9830005G06Rik;DNGR-1;CD370;DNGR-1;DNGR1;UNQ9341	ENSG00000197992	type 1 dendritic cells (DC1)
CD1c molecule	CD1C	HGNC: 1636	BDCA1;CD1;CD1A;R7	ENSG00000158481	type 2 dendriti

					c cells (DC2)
C-type lectin domain containing 10A	CLEC10A	HGNC: 16916	CD301a;M-ASGP-BP-1;Mgl;Mgl1;CD301;CLECSF13;CLECSF14;HML;HML2;MGL	ENSG00000132514	type 2 dendritic cells (DC2)
indoleamine 2,3-dioxygenase 1	IDO1	HGNC: 6059	Ido;Indo;IDO;IDO-1;INDO	ENSG00000131203	IDO1+ myeloid dendritic cells (mDC)
leukocyte specific transcript 1	LST1	HGNC: 14189	B144;B144;D6S49E;LST-1	ENSG00000204482	innate lymphoid cells (ILC)
CD7 molecule	CD7	HGNC: 1695	GP40;LEU-9;TP41;Tp40	ENSG00000173762	natural killer (NK) cells
killer cell lectin like receptor C2	KLRC2	HGNC: 6375	NKG2C;CD159c;NKG2-C;NKG2C	ENSG00000205809	natural killer (NK) cells
killer cell lectin like receptor D1	KLRD1	HGNC: 6378	CD94;CD94	ENSG00000134539	natural killer (NK) cells
transmembrane immune signaling adaptor TYROBP	TYROBP	HGNC: 12449	DAP12;KARAP;Ly83;DAP12;KARAP;PLOSL;PLOSL1	ENSG00000011600	natural killer (NK) cells
natural killer cell granule protein 7	NKG7	HGNC: 7830	2500004F03Rik;GIG1;GMP-17;p15-TIA-1	ENSG00000105374	natural killer (NK) cells/ NKT cells
CD3d molecule	CD3D	HGNC: 1673	T3d;CD3-DELTA;IMD19;T3D	ENSG00000167286	T cells
CD3e molecule	CD3E	HGNC: 1674	AI504783;CD3;CD3epsilon;T3e;IMD18;T3E;TCRE	ENSG00000198851	T cells
CD3g molecule	CD3G	HGNC: 1675	Ctg-3;Ctg3;T3g;CD3-GAMMA;IMD17;T3G	ENSG00000160654	T cells
CD28 molecule	CD28	HGNC: 1653	Tp44	ENSG00000178562	T cells, T cell activation
CD4 molecule	CD4	HGNC: 1678	L3T4;Ly-4;CD4mut	ENSG00000010610	CD4+ T cells
CD8a molecule	CD8A	HGNC: 1706	BB154331;Ly-2;Ly-35;Ly-B;Lyt-2;CD8;Leu2;p32	ENSG00000153563	CD8+ T cells
CD8b molecule	CD8B	HGNC: 1707	CD8B1;LEU2;LY3;LYT3;P37	ENSG00000172116	CD8+ T cells

CD8 antigen, beta chain 1			Cd8b;Ly-3;Ly-C;Lyt-3		CD8+ T cells
interleukin 2 receptor subunit alpha	IL2RA	HGNC: 6008	CD25;Il2r;Ly-43;CD25;IDDM10;IL2R;IMD41;TCGFR;p55	ENSG00000134460	mouse regulatory T cells (Treg); human Th1 effector
killer cell lectin like receptor B1	KLRB1	HGNC: 6373	4930431A04Rik;Gm4696;Klrb1g;Klrb6;Ly-55;Ly55;NKR-P1G;Nkrp-1e;Nkrp1g;CD161;CLEC5B;NKR;NKR-P1;NKR-P1A;NKRP1A;hNKR-P1A	ENSG00000111796	mucosal-associated invariant T cells (MAIT)
forkhead box P3	FOXP3	HGNC: 6106	JM2;scurfin;sf;AIID;DIETER;IPEX;JM2;PIDX;XPID	ENSG00000049768	regulatory T cells (Treg)
C-X-C motif chemokine receptor 3	CXCR3	HGNC: 4540	Cd183;Cmkar3;CD182;CD183;CKR-L2;CMKAR3;GPR9;IP10-R;Mig-R;MigR	ENSG00000186810	type 1 T helper cells (Th1)
C-C motif chemokine receptor 4	CCR4	HGNC: 1605	C-C CKR-4;CHEMR1;Cmkbr4;LESTR;Sdf1r;CC-CKR-4;CD194;CKR4;CMKBR4;ChemR13;HGCN:14099;K5-5	ENSG00000183813	type 2 T helper cells (Th2)
joining chain of multimeric IgA and IgM	JCHAIN	HGNC: 5713	9530090F24Rik;AI323815;Igj;Jch;IGCJ;IGJ;JCH	ENSG00000132465	mucosal-targeted plasma cells
G protein-coupled receptor 183	GPR183	HGNC: 3128	Ebi2;EBI2;hEBI2	ENSG00000169508	memory cells
CD19 molecule	CD19	HGNC: 1633	AW495831;B4;CVID3	ENSG00000177455	B cells
CD79a molecule	CD79A	HGNC: 1698	Ig-alpha;Iga;Igalpha;Ly-54;Ly54;mb-1;IGA;MB-1	ENSG00000105369	B cells
CD79b molecule	CD79B	HGNC: 1699	B29;Ig-beta;Igb;Igbeta;AGM6;B29;IGB	ENSG00000007312	B cells
membrane spanning 4-domains A1	MS4A1	HGNC: 7315	AA960661;Cd20;Ly-44;Ms4a2;B1;Bp35;CD20;CVID5;LEU-16;MS4A2;S7	ENSG00000156738	B cells

immunoglobulin kappa constant	IGKC	HGNC: 5716	Igk-C;HCAK1;IGKCD;Km	ENSG00000211592	plasma cells

Supplementary Table 4: List of predefined Immune Regulation gene panel

Gene full name	Gene symbol	HGNC ID	Alias	ENSEMBL GeneID human	Annota-tion
interleukin 7	IL7	HGNC:6023	A630026I06Rik;Il-7;hlb368;IL-7	ENSG00000104432	differentiation of hematopoietic stem cells into lymphoid progenitor cells
C-C motif chemokine receptor 5	CCR5	HGNC:1606	AM4-7;CD195;Cmkbr5;CC-CKR-5;CCCKR5;CCR-5;CD195;CKR-5;CKR5;CMKBR5;IDDM22	ENSG00000160791	granulocyte lineage differentiation
basic leucine zipper ATF-like transcription factor 3	BATF3	HGNC:28915	9130211I03Rik;Snft;JDP1;JUNDM1;SNFT	ENSG00000123685	dendritic cell (DC) development, potential target in immunosupressed tumor
interleukin 7 receptor	IL7R	HGNC:6024	CD127;IL-7Ralpha;CD127;CDW127;IL-7R-alpha;IL7RA;ILRA	ENSG00000168685	V(D)J recombination
lymphoid enhancer binding factor 1	LEF1	HGNC:6551	3000002B05;AI451430;Lef-1;LEF-1;TCF10;TCF1ALPHA;TCF7L3	ENSG00000138795	B and T cell differentiation
C-C motif chemokine receptor 6	CCR6	HGNC:1607	CC-CKR-6;CCR-6;Cmkbr6;KY411;BN-1;C-C CKR-6;CC-CKR-6;CCR-6;CD196;CKR-L3;CKRL3;CMKBR6;DCR2;DRY6;GPR29;GPRCY4;STRL22	ENSG00000112486	B cell maturation and differentation
interleukin 2	IL2	HGNC:6001	Il-2;IL-2;TCGF;lymphokine	ENSG00000109471	T cell differentiation and immune tolerance, Th1 effector

neural cell adhesion molecule 1	NCAM1	HGNC:7656	CD56;E-NCAM;NCAM-1;Ncam;CD56;MSK39;NCAM	ENSG00000149294	T cell expansion, neural development
T-box transcription factor 21	TBX21	HGNC:11599	TBT1;Tbet;Tblym;T-PET;T-bet;TBET;TBLYM	ENSG00000073861	controls expression of Th1 cytokine IFNG
interferon gamma	IFNG	HGNC:5438	IFN-g;Ifg;IFG;IFI	ENSG00000111537	Th1 development, diverse functions
interleukin 5	IL5	HGNC:6016	Il-5;EDF;IL-5;TRF	ENSG00000113525	Th2 development
interleukin 4	IL4	HGNC:6014	BSF-1;Il-4;BCGF-1;BCGF1;BSF-1;BSF1;IL-4	ENSG00000113520	Th2 development, diverse functions
GATA binding protein 3	GATA3	HGNC:4172	Gata-3;jal;HDR;HDRS	ENSG00000107485	Th2 helper cell development
C-C motif chemokine receptor 7	CCR7	HGNC:1608	CC-CKR-7;CCR-7;CD197;Cdw197;Cmkbr7;EBI1;Ebi1h;BLR2;CC-CKR-7;CCR-7;CD197;CDw197;CMKBR7;EBI1	ENSG00000126353	dendritic cell (DC) activation
CD44 molecule (Indian blood group)	CD44	HGNC:1681	AU023126;AW121933;AW146109;HERMES;Ly-24;Pgp-1;CDW44;CSPG8;ECMR-III;HCELL;HUTCH-I;IN;LHR;MC56;MDU2;MDU3;MIC4;Pgp1	ENSG00000026508	lymphocyte activation
peroxisome proliferator activated receptor gamma	PPARG	HGNC:9236	Nr1c3;PPAR-gamma;PPAR-gamma2;PPARgamma;PPARgamma2;CIMT1;GLM1;NR1C3;PPARG1;PPARG2;PPARG5;PPARgamma	ENSG00000132170	M2 macrophage activation, lipotoxicity
icos ligand			AU044799;B7-H2;B7RP-1;B7h;BG071784;Gl50;GL50;GL50-B;ICOS-L;Icoslg;LICOS;Ly115l;mKIAA0653		B and T cell activation
inducible T cell costim	ICOSLG	HGNC:17087	B7-H2;B7H2;B7RP-1;B7RP1;B7h;CD275;GL50;ICOS-L;ICOSL;LICOS	ENSG00000160223	B and T cell activation

ulator ligand					
CD40 ligand	CD40LG	HGNC:11935	CD154;CD40-L;Cd40l;HIGM1;IGM;IMD3;Ly-62;Ly62;T-BAM;TRAP;Tnfsf5;Tnlg8b;gp39;CD154;CD40L;HIGM1;IGM;IMD3;T-BAM;TNFSF5;TRAP;gp39;hCD40L	ENSG00000102245	activated T cells
interleukin 17A	IL17A	HGNC:5981	Ctla-8;Ctla8;IL-17;IL-17A;Il17;CTLA-8;CTLA8;IL-17;IL-17A;IL17	ENSG00000112115	activated T cells
CD86 molecule	CD86	HGNC:1705	B7;B7-2;B7.2;B70;CLS1;Cd28l2;ETC-1;Ly-58;Ly58;MB7;MB7-2;TS/A-2;B7-2;B7.2;B70;CD28LG2;LAB72	ENSG00000114013	T cell activation
C-X-C motif chemokine receptor 5	CXCR5	HGNC:1060	Blr1;CXC-R5;CXCR-5;Gpcr6;MDR15;BLR1;CD185;MDR15	ENSG00000160683	follicular helper T cells
eomesodermin	EOMES	HGNC:3372	C77258;TBR-2;Tbr2;TBR2	ENSG00000163508	T cell exhaustion
CD83 molecule	CD83	HGNC:1703	BL11;HB15	ENSG00000112149	antigen presenting and immune stimulation
triggering receptor expressed on myeloid cells 2	TREM2	HGNC:17761	TREM-2;Trem2a;Trem2b;Trem2c;PLOSL2;TREM-2;Trem2a;Trem2b;Trem2c	ENSG00000095970	anti-inflammation
complement C3	C3	HGNC:1318	AI255234;ASP;HSE-MSF;Plp;AHUS5;ARMD9;ASP;C3a;C3b;CPAMD1;HEL-S-62p	ENSG00000125730	complement system
C-X-C motif chemokine receptor 6	CXCR6	HGNC:16647	BB217514;BONZO;STRL33;BONZO;CD186;STRL33;TYMSTR	ENSG00000172215	induced by inflammatory stimuli, T cell recruiting signal
tumor necrosis factor	TNF	HGNC:11892	DIF;TNF-a;TNF-alpha;TNFSF2;TNFalpha;Tnfa;Tnfsf1a;Tnlg1f;DIF;TNF-alpha;TNFA;TNFSF2;TNLG1F	ENSG00000232810	pro-inflammation, diverse functions
interleukin 22	IL22	HGNC:14900	IL-22;IL-22a;ILTIFa;Iltif;IL-21;IL-22;IL-D110;IL-TIF;ILTIF;TIFIL-23;TIFa;zcyto18	ENSG00000127318	chronic inflammation, effect on stromal and

					epithelial cells
Fc fragment of IgG receptor IIa	FCGR2A	HGNC:3616	CD32;CD32A;CDw32;FCG2;FCGR2;FCGR2A1;FcGR;IGFR2	ENSG00000143226	low affinity inhibitory receptor for immunoglobulin gamma Fc region
Fc fragment of IgG receptor IIb	FCGR2B	HGNC:3618	AI528646;CD32;F630109E10Rik;Fc[g]RII;FcgRII;Fcgr2;Fcgr2a;Fcr-2;Fcr-3;Ly-17;Ly-m20;LyM-1;Lym-1;fcRII;CD32;CD32B;FCG2;FCGR2;FCGR2C;FcRII-c;IGFR2	ENSG00000072694	low affinity inhibitory receptor for immunoglobulin gamma Fc region
C-X-C motif chemokine ligand 5	CXCL5	HGNC:10642	AMCF-II;Cxcl6;ENA-78;GCP-2;LIX;Scyb5;Scyb6;ENA-78;SCYB5	ENSG00000163735	chemotaxis of neutrophils
C-X3-C motif chemokine receptor 1	CX3CR1	HGNC:2558	CCRL1;CMKBRL1;CMKDR1;GPR13;GPRV28;V28	ENSG00000168329	immune cell recruitment
chemokine (C-X-C motif) ligand 15			Il8;Scyb15;lungkine;weche		immune cell recruitment
C-X-C motif chemokine ligand 8	CXCL8	HGNC:6025	GCP-1;GCP1;IL8;LECT;LUCT;LYNAP;MDNCF;MONAP;NAF;NAP-1;NAP1	ENSG00000169429	immune cell recruitment
C-C motif chemokine receptor 2	CCR2	HGNC:1603	Cc-ckr-2;Ccr2a;Ccr2b;Ckr2;Ckr2a;Ckr2b;Cmkbr2;mJe-r;CC-CKR-2;CCR-2;CCR2A;CCR2B;CD192;CKR2;CKR2A;CKR2B;CMKBR2;MCP-1-R	ENSG00000121807	migration of monocytes

Supplementary Table 5 :Top fifty differentially expressed genes in epidermal cell clusters

		p_val	avg_log2FC	pct.1	pct.2	p_val_adj	cluster	gene
1	KRT1	0	4.28809694757089	0.896	0.154	0	IFE spinous K	KRT1
2	KRT10	0	3.5175919811946	0.965	0.712	0	IFE spinous K	KRT10
3	DMKN	0	3.42095305589691	0.952	0.403	0	IFE spinous K	DMKN
4	KRTDAP	0	3.15599488380837	0.808	0.117	0	IFE spinous K	KRTDAP
5	LY6D	0	3.10145316332221	0.916	0.241	0	IFE spinous K	LY6D
6	LYPD3	0	2.58553405616127	0.867	0.3	0	IFE spinous K	LYPD3
7	KRT2	0	2.52876664471233	0.281	0.026	0	IFE spinous K	KRT2
8	LGALS7B	0	2.17349137885373	0.933	0.514	0	IFE spinous K	LGALS7B
9	SFN	0	1.86021255395409	0.98	0.791	0	IFE spinous K	SFN
10	PHLDA2	0	1.63218678200726	0.681	0.25	0	IFE spinous K	PHLDA2
11	GNB2L1	0	1.55483431980857	0.806	0.233	0	IFE spinous K	GNB2L1
12	SBSN	0	1.53313710877213	0.659	0.127	0	IFE spinous K	SBSN
13	AQP3	0	1.53303386626631	0.91	0.484	0	IFE spinous K	AQP3
14	DEFB1	0	1.48084888644785	0.576	0.145	0	IFE spinous K	DEFB1
15	MAL2	0	1.37220466922112	0.544	0.127	0	IFE spinous K	MAL2
16	RHOV	0	1.3629266307234	0.569	0.165	0	IFE spinous K	RHOV
17	S100A14	0	1.3580794304785	0.977	0.827	0	IFE spinous K	S100A14
18	PERP	0	1.35092705145579	0.989	0.919	0	IFE spinous K	PERP
19	TPPP3	0	1.33566102195516	0.542	0.118	0	IFE spinous K	TPPP3
20	ATP5G2	0	1.30000510160783	0.691	0.18	0	IFE spinous K	ATP5G2
21	ATP5E	0	1.280322427823	0.683	0.174	0	IFE spinous K	ATP5E
22	CCL27	0	1.26648963968756	0.697	0.25	0	IFE spinous K	CCL27
23	TACSTD2	0	1.26131576336589	0.902	0.519	0	IFE spinous K	TACSTD2
24	TCEB2	0	1.23110119311982	0.642	0.158	0	IFE spinous K	TCEB2
25	ATP5L	0	1.19135894225817	0.699	0.189	0	IFE spinous K	ATP5L
26	GLTSCR2	0	1.187448057384	0.709	0.203	0	IFE spinous K	GLTSCR2
27	NHP2L1	0	1.16376717760736	0.616	0.165	0	IFE spinous K	NHP2L1
28	RPSAP58	0	1.14462075011701	0.554	0.141	0	IFE spinous K	RPSAP58
29	KLK11	0	1.1198466899915	0.462	0.112	0	IFE spinous K	KLK11
30	SELK	0	1.0867090041099	0.587	0.156	0	IFE spinous K	SELK
31	SF3B14	0	1.07606982623648	0.544	0.137	0	IFE spinous K	SF3B14
32	VIMP	0	0.989252806889191	0.455	0.102	0	IFE spinous K	VIMP
33	C14orf2	0	0.983983732509313	0.487	0.113	0	IFE spinous K	C14orf2
34	ATP5D	0	0.968380579166539	0.552	0.138	0	IFE spinous K	ATP5D
35	SERPINB5	1.17093528842005E-295	1.19025804135528	0.82	0.51	4.28152488210791E-291	IFE spinous K	SERPINB5
36	NEAT1	1.51145499961192E-286	1.3031138437133	0.923	0.662	5.52663520608099E-282	IFE spinous K	NEAT1
37	DSG1	3.4841150977596E-286	1.10315292857255	0.574	0.213	1.2739666854958E-281	IFE spinous K	DSG1
38	ALDOA	6.3304868026916E-284	0.968946424214359	0.727	0.324	2.31474249940418E-279	IFE spinous K	ALDOA
39	EIF4A1	1.43219709538889E-262	0.989834089134385	0.902	0.71	5.23682867928949E-258	IFE spinous K	EIF4A1
40	NBEAL1	1.4076580682825E-253	1.25078440497401	0.782	0.513	5.14710172667495E-249	IFE spinous K	NBEAL1
41	RORA	1.91498595440436E-243	0.990479907034103	0.55	0.213	7.00214614227953E-239	IFE spinous K	RORA
42	FGFBP1	6.61417733409839E-236	1.00370300783653	0.589	0.22	2.41847394221308E-231	IFE spinous K	FGFBP1
43	RNF144B	2.917834767704E-221	1.92107828233096	0.46	0.167	1.06690628281097E-216	IFE spinous K	RNF144B
44	NRARP	7.68915219005607E-209	1.01729721791266	0.486	0.192	2.811538498294E-204	IFE spinous K	NRARP
45	KRT16	1.27870505387583E-205	1.57077948142547	0.508	0.207	4.67558502949698E-201	IFE spinous K	KRT16
46	KLF4	3.20455148825929E-191	1.0425964800299	0.725	0.482	1.17174425168201E-186	IFE spinous K	KLF4

47	MYC	8.95346428888708E-177	1.07756500364773	0.74	0.486	3.27383421723156E-172	IFE spinous K	MYC
48	MPZL2	6.47943935466579E-170	1.05356995333786	0.564	0.302	2.36920700003355E-165	IFE spinous K	MPZL2
49	GLTP	4.43918979468173E-151	0.993972205543968	0.537	0.304	1.62318974842537E-146	IFE spinous K	GLTP
50	LGALS3	9.35477934700372E-113	0.970217046425054	0.799	0.686	3.42057506823191E-108	IFE spinous K	LGALS3
51	SERPINB21	0	2.39127406473373	0.61	0.192	0	IFE basal K	SERPINB2
52	KRT14	0	2.10254893541129	0.989	0.909	0	IFE basal K	KRT14
53	SYT8	3.65248662410128E-299	1.32708825438225	0.33	0.047	1.33553173408489E-294	IFE basal K	SYT8
54	MIR205HG1	9.7907751400683E-294	1.91525299989662	0.639	0.256	3.57999692996597E-289	IFE basal K	MIR205HG
55	S100A2	8.62923675859782E-263	1.59723469949578	0.952	0.762	3.15528042078129E-258	IFE basal K	S100A2
56	ERRFI11	4.37337478188327E-260	1.79394551229648	0.645	0.283	1.59912448899562E-255	IFE basal K	ERRFI1
57	POSTN	5.58519074322894E-257	1.18579266614842	0.457	0.118	2.04222499526166E-252	IFE basal K	POSTN
58	SAT1	3.12944860870619E-221	1.26983768683746	0.861	0.585	1.14428288377342E-216	IFE basal K	SAT1
59	COL17A1	1.26004863372555E-218	1.20102446177665	0.773	0.433	4.60736782921748E-214	IFE basal K	COL17A1
60	LIMA1	3.15788681157659E-213	1.25398904951328	0.699	0.392	1.15468131265298E-208	IFE basal K	LIMA1
61	LDHA1	2.70380443742699E-200	0.997666603048221	0.906	0.749	9.88646092545178E-196	IFE basal K	LDHA
62	IGFBP3	3.49022837419743E-190	1.41244816974942	0.3	0.065	1.27620200502529E-185	IFE basal K	IGFBP3
63	AREG	1.68605116033374E-187	2.21229502736561	0.475	0.182	6.16504606776034E-183	IFE basal K	AREG
64	AQP31	1.17660076008329E-186	1.01603886856943	0.839	0.524	4.30224067924457E-182	IFE basal K	AQP3
65	PHLDB2	2.07515595807245E-185	1.04732830306991	0.354	0.095	7.5878077606919E-181	IFE basal K	PHLDB2
66	SFN1	3.1822607567598E-179	0.936960953318035	0.948	0.808	1.16359364570922E-174	IFE basal K	SFN
67	TNFRSF12A1	5.65832564510179E-178	1.05581437808252	0.709	0.413	2.06896677213147E-173	IFE basal K	TNFRSF12A
68	ADRB21	6.69358385081302E-170	1.05128982472957	0.505	0.205	2.44750893504978E-165	IFE basal K	ADRB2
69	PTRF	9.28791530883414E-166	0.885523320284473	0.346	0.099	3.3961262326752E-161	IFE basal K	PTRF
70	FGFBP11	3.63869347191511E-152	1.22468882583845	0.546	0.25	1.33048826800576E-147	IFE basal K	FGFBP1
71	GPX1	1.34301313637968E-147	0.810903230481067	0.339	0.104	4.91072753317231E-143	IFE basal K	GPX1
72	ANXA1	1.98856575622853E-142	0.829551151385831	0.871	0.691	7.27119068764961E-138	IFE basal K	ANXA1
73	CAV1	4.17521836744069E-139	0.943667571592148	0.83	0.646	1.52666859605469E-134	IFE basal K	CAV1
74	GLTSCR21	7.54372055944648E-139	0.948623661672133	0.567	0.261	2.7583614225616E-134	IFE basal K	GLTSCR2
75	TACSTD21	2.77759783701921E-138	0.825060211260801	0.833	0.555	1.01562864910607E-133	IFE basal K	TACSTD2
76	SDCBP	3.36154250231298E-138	0.877788364587187	0.574	0.309	1.22914801597074E-133	IFE basal K	SDCBP
77	EMP11	1.15952606943981E-136	0.860490382215512	0.711	0.437	4.23980707290668E-132	IFE basal K	EMP1
78	CALM21	3.63802882573954E-135	0.824026335301255	0.899	0.813	1.33024524013166E-130	IFE basal K	CALM2
79	RND3	5.46011175325956E-135	1.0715752793977	0.819	0.624	1.99648986257936E-130	IFE basal K	RND3
80	NFKBIA1	3.21534509084336E-128	1.06769456339323	0.91	0.784	1.17569093246687E-123	IFE basal K	NFKBIA
81	ARID5B	1.78651915630658E-123	1.06929732111002	0.769	0.589	6.53240729503501E-119	IFE basal K	ARID5B
82	GADD45A	1.95160876149306E-122	1.13334290930005	0.605	0.362	7.13605743639937E-118	IFE basal K	GADD45A
83	PFN11	3.71638030498428E-122	0.832367290424064	0.879	0.769	1.3588944585175E-117	IFE basal K	PFN1
84	SELK1	1.50680476743318E-121	0.879193158632981	0.466	0.206	5.50963163211942E-117	IFE basal K	SELK
85	CYR61	5.7676368570684E-121	1.06917432327834	0.689	0.447	2.10893641678706E-116	IFE basal K	CYR61
86	ALDOA1	3.84002392784405E-120	0.88642333697467	0.624	0.368	1.40410474921618E-115	IFE basal K	ALDOA
87	U2AF11	1.3349041186664E-118	0.866862613322644	0.474	0.218	4.8810769099037E-114	IFE basal K	U2AF1
88	PDLIM1	1.90322608261247E-118	0.86974469724352	0.652	0.453	6.95914617107248E-114	IFE basal K	PDLIM1
89	EIF4A11	3.02876750384125E-118	0.853041046557326	0.867	0.728	1.10746883777955E-113	IFE basal K	EIF4A1
90	CCL271	6.81654660066868E-116	1.02450939519693	0.566	0.302	2.4924702645345E-111	IFE basal K	CCL27
91	PKM	2.65065745046374E-111	0.849398320566586	0.735	0.572	9.69212896762067E-107	IFE basal K	PKM
92	SOX151	1.27045905345969E-109	0.841951882282137	0.493	0.256	4.64543352897536E-105	IFE basal K	SOX15
93	TCEB11	1.24116820615345E-108	0.799544421081126	0.367	0.148	4.5383315458001E-104	IFE basal K	TCEB1
94	MYC1	5.57260714881232E-107	0.855007851521906	0.73	0.503	2.03762380396322E-102	IFE basal K	MYC

95	CD551	5.14348209215873E-106	0.924637456949443	0.626	0.393	1.88071422699784E-101	IFE basal K	CD55
96	PMAIP1	3.5811091544009E-104	0.919660185951314	0.756	0.585	1.30943256230669E-99	IFE basal K	PMAIP1
97	CCL2	6.89187020578609E-102	0.992973297308523	0.424	0.195	2.52001234074568E-97	IFE basal K	CCL2
98	PTP4A11	5.10930987316108E-98	0.864896579898651	0.673	0.48	1.86821915512135E-93	IFE basal K	PTP4A1
99	ITGA6	8.74667699236139E-86	0.871297225522565	0.424	0.23	3.19822244225694E-81	IFE basal K	ITGA6
100	CA21	2.13460974090127E-45	0.79888334602027	0.283	0.151	7.8052005176055E-41	IFE basal K	CA2
101	MOXD1	1.86387366628512E-214	0.68593183813381	0.415	0.113	6.81525406077154E-210	Transitional K	MOXD1
102	SAA1	4.40498259500298E-207	0.966809609725388	0.379	0.092	1.61068188586284E-202	Transitional K	SAA1
103	C1QTNF12	8.00179672614379E-198	0.756658961574324	0.338	0.08	2.92585697291448E-193	Transitional K	C1QTNF12
104	FOS	3.40660485936278E-191	1.22124164886282	0.904	0.771	1.245625066826E-186	Transitional K	FOS
105	JUN	5.16666613402553E-177	1.00585408526226	0.958	0.85	1.88919147190643E-172	Transitional K	JUN
106	EGR2	3.08621419110227E-168	0.687107012960899	0.464	0.167	1.12847421897655E-163	Transitional K	EGR2
107	MTRNR2L12	2.25599596020713E-157	0.866161665388709	0.831	0.522	8.24904922849738E-153	Transitional K	MTRNR2L12
108	JUND	6.52478601790207E-146	0.768951155135251	0.886	0.688	2.38578800744589E-141	Transitional K	JUND
109	FOXC1	2.53598867264422E-140	0.659673768276504	0.51	0.218	9.2728425815236E-136	Transitional K	FOXC1
110	IER3	1.66866822460337E-136	0.992975134080944	0.871	0.663	6.1014853632622E-132	Transitional K	IER3
111	IRF1	3.92512409439916E-131	1.06943645049462	0.603	0.313	1.43522162511705E-126	Transitional K	IRF1
112	IER5	1.8202182298917E-122	1.00086254860096	0.68	0.383	6.65562795759899E-118	Transitional K	IER5
113	RACK1	7.04820690160846E-120	0.73298886368978	0.825	0.534	2.57717685357313E-115	Transitional K	RACK1
114	ATF3	8.81576153764752E-117	0.816515862846243	0.731	0.477	3.22348320624082E-112	Transitional K	ATF3
115	FOSB	2.66214630833738E-114	0.939191614114051	0.798	0.571	9.73413797643562E-110	Transitional K	FOSB
116	CTGF	5.08615135044151E-113	0.924970554491371	0.369	0.142	1.85975124128894E-108	Transitional K	CTGF
117	FST	6.88747807473639E-110	0.655429462087396	0.325	0.112	2.51840635802736E-105	Transitional K	FST
118	SOCS31	1.55079326608776E-103	1.00898935027396	0.56	0.291	5.67047557744988E-99	Transitional K	SOCS3
119	PIK3R1	2.13028972977881E-102	0.74893955927469	0.589	0.314	7.78940439693622E-98	Transitional K	PIK3R1
120	IER2	2.6047156597137E-98	0.742395158178486	0.843	0.695	9.52414280974314E-94	Transitional K	IER2
121	EGR1	2.44749990903391E-94	0.764194529533043	0.735	0.506	8.94928341738251E-90	Transitional K	EGR1
122	CCNL1	5.06325431278301E-92	0.820050026414783	0.805	0.607	1.85137893946911E-87	Transitional K	CCNL1
123	NCOA7	4.00187829032833E-90	0.999060021914973	0.491	0.254	1.46328679685855E-85	Transitional K	NCOA7
124	HSPA1A	2.45383883319359E-89	1.01849108309757	0.935	0.769	8.97246169357237E-85	Transitional K	HSPA1A
125	CYR611	5.18913028273286E-89	0.685203884059526	0.705	0.442	1.89740548788127E-84	Transitional K	CYR61
126	CEBPD	1.07898319239403E-82	0.86188810329515	0.804	0.606	3.94530204298877E-78	Transitional K	CEBPD
127	MT-ND3	3.10522864875687E-76	0.663849846639803	0.925	0.86	1.13542685541795E-71	Transitional K	MT-ND3
128	PPP1R15A1	1.46348249148555E-65	0.925547325459459	0.857	0.678	5.3512237301169E-61	Transitional K	PPP1R15A
129	BTG2	5.60779915992684E-62	0.674829955977282	0.723	0.521	2.05049176282725E-57	Transitional K	BTG2
130	VMP1	6.40301225573442E-62	0.7942109303193	0.609	0.395	2.34126143130929E-57	Transitional K	VMP1
131	INTS6	3.67077130441215E-59	0.706981554634191	0.561	0.358	1.3422175274583E-54	Transitional K	INTS6
132	SOX4	9.90354689409924E-58	0.716782600789018	0.762	0.521	3.62123192182739E-53	Transitional K	SOX4
133	HSPA1B	4.8101893541441E-57	0.905756257596731	0.865	0.693	1.75884573734279E-52	Transitional K	HSPA1B
134	STAT3	6.05865115221113E-57	0.668423628609565	0.643	0.434	2.215345793806E-52	Transitional K	STAT3
135	CSKMT	1.19599058646576E-56	0.696245045469325	0.251	0.11	4.37313957941207E-52	Transitional K	CSKMT
136	HES1	3.30715468184488E-56	0.705817289053471	0.667	0.46	1.20926110941658E-51	Transitional K	HES1
137	NR4A1	1.87771820390203E-54	0.673535282481217	0.677	0.479	6.86587661256778E-50	Transitional K	NR4A1
138	HSP90AA1	3.89440536119888E-53	0.80085691060957	0.968	0.919	1.42398932032237E-48	Transitional K	HSP90AA1
139	MALAT1	5.1784176110174E-51	0.811051948324652	0.991	0.983	1.89348839946851E-46	Transitional K	MALAT1
140	TSPYL2	9.2796763487652E-47	0.676238358166486	0.344	0.19	3.39311365692599E-42	Transitional K	TSPYL2
141	THBS1	4.42375161736147E-46	0.66105422462142	0.318	0.17	1.61754477888822E-41	Transitional K	THBS1
142	RASD1	1.04838725229954E-45	0.812725738031706	0.343	0.193	3.83342798803328E-41	Transitional K	RASD1

143	TSC22D11	4.76051775918467E-44	0.649276313864039	0.7	0.526	1.74068331864588E-39	Transitional K	TSC22D1
144	NRP2	1.34526428608476E-41	0.784931519304436	0.284	0.156	4.91895886206894E-37	Transitional K	NRP2
145	HSPH11	5.82840424631901E-40	0.785441533775431	0.623	0.455	2.13115601266655E-35	Transitional K	HSPH1
146	GADD45B	1.39087313448713E-39	0.98333904565412	0.657	0.508	5.0857276162522E-35	Transitional K	GADD45B
147	HNRNPU	3.1713443246862E-36	0.780227560876142	0.803	0.665	1.15960205232151E-31	Transitional K	HNRNPU
148	DNAJB1	1.81653965394439E-35	0.781413282321334	0.857	0.701	6.64217724464766E-31	Transitional K	DNAJB1
149	MT2A1	1.10353821913058E-16	0.681835032044198	0.749	0.63	4.03508749825096E-12	Transitional K	MT2A
150	CCL21	3.97083546527737E-15	1.09357990236354	0.295	0.221	1.45193598787867E-10	Transitional K	CCL2
151	IGKC	0	3.34016550069372	0.496	0.084	0	Outer bulb K	IGKC
152	SFRP1	0	2.37476345597226	0.578	0.115	0	Outer bulb K	SFRP1
153	EDIL3	0	2.12069686878844	0.533	0.161	0	Outer bulb K	EDIL3
154	PTCH1	0	2.0599179342857	0.586	0.122	0	Outer bulb K	PTCH1
155	LMO3	0	1.94462366170756	0.424	0.047	0	Outer bulb K	LMO3
156	CALD1	0	1.84096721307396	0.87	0.535	0	Outer bulb K	CALD1
157	SELENOP	0	1.82137556956982	0.692	0.297	0	Outer bulb K	SELENOP
158	BNC2	0	1.74015440690921	0.713	0.182	0	Outer bulb K	BNC2
159	EPCAM	0	1.65636126495156	0.545	0.143	0	Outer bulb K	EPCAM
160	ABI3BP1	0	1.63266876441708	0.541	0.155	0	Outer bulb K	ABI3BP
161	GJB6	0	1.52897896123769	0.703	0.24	0	Outer bulb K	GJB6
162	BGN	0	1.50387087332417	0.634	0.179	0	Outer bulb K	BGN
163	IGFBP7	0	1.45759987643812	0.686	0.267	0	Outer bulb K	IGFBP7
164	SOX41	0	1.4255440544462	0.847	0.488	0	Outer bulb K	SOX4
165	BCAM	0	1.36181209188633	0.744	0.311	0	Outer bulb K	BCAM
166	DIO2	0	1.31233415256869	0.456	0.095	0	Outer bulb K	DIO2
167	RAMP1	0	1.29780038351867	0.497	0.114	0	Outer bulb K	RAMP1
168	TFAP2B1	0	1.29408081094213	0.613	0.215	0	Outer bulb K	TFAP2B
169	ITM2A	0	1.27098175249057	0.598	0.209	0	Outer bulb K	ITM2A
170	PTCH2	0	1.24041929086675	0.407	0.076	0	Outer bulb K	PTCH2
171	TBX1	0	1.20237082824429	0.498	0.101	0	Outer bulb K	TBX1
172	LHX2	0	1.17210811056919	0.448	0.107	0	Outer bulb K	LHX2
173	LGR5	0	1.1518305709177	0.38	0.059	0	Outer bulb K	LGR5
174	IGF2BP2	1.42922685868253E-298	1.09357545177483	0.476	0.12	5.22596800878069E-294	Outer bulb K	IGF2BP2
175	CXCL141	8.30067827931122E-296	1.87823778451445	0.911	0.7	3.03514301283014E-291	Outer bulb K	CXCL14
176	CREB5	8.16290204280912E-291	1.06748526768888	0.598	0.21	2.98476513195315E-286	Outer bulb K	CREB5
177	MYL9	2.9008167384762E-286	1.83228149944875	0.469	0.14	1.06068364042382E-281	Outer bulb K	MYL9
178	SPON2	8.45420360781774E-279	1.75183678293827	0.534	0.179	3.09127954919856E-274	Outer bulb K	SPON2
179	ADGRL3	6.96072869775375E-276	1.24104169396908	0.519	0.181	2.54519044833366E-271	Outer bulb K	ADGRL3
180	SCD5	1.42899616892398E-275	1.20076931345475	0.379	0.084	5.22512449167054E-271	Outer bulb K	SCD5
181	KRT17	1.06941194575666E-274	1.01583122956196	0.875	0.536	3.91030477965921E-270	Outer bulb K	KRT17
182	SDC2	2.62180808159957E-270	1.12386448607894	0.482	0.146	9.58664125036882E-266	Outer bulb K	SDC2
183	NFIB1	2.22993813514278E-266	1.18768521471735	0.851	0.559	8.15376879114959E-262	Outer bulb K	NFIB
184	MYLK	4.81738218623566E-266	2.00537361330758	0.41	0.107	1.76147579639707E-261	Outer bulb K	MYLK
185	BEX3	4.26923228464952E-257	1.06512063952817	0.603	0.265	1.5610447848821E-252	Outer bulb K	BEX3
186	IGLC2	6.70981381296984E-255	1.12567880547337	0.286	0.042	2.45344342071242E-250	Outer bulb K	IGLC2
187	MDK	9.19033032742843E-250	1.28899878079285	0.499	0.18	3.36044428422421E-245	Outer bulb K	MDK
188	PDGFA	5.58849120013825E-246	1.34789222323785	0.549	0.222	2.04343180733055E-241	Outer bulb K	PDGFA
189	SLC26A2	1.7948968861197E-244	1.13784501371432	0.366	0.086	6.56304046409669E-240	Outer bulb K	SLC26A2
190	PCNT	6.82508223700309E-231	1.42927954534943	0.389	0.108	2.49559131996018E-226	Outer bulb K	PCNT

191	PSD3	2.47678836366963E-223	1.12391938750024	0.446	0.149	9.056376651758E-219	Outer bulb K	PSD3
192	DAPL1	7.20453534654517E-221	1.45930574923578	0.739	0.473	2.63433834946424E-216	Outer bulb K	DAPL1
193	TAGLN	9.20005614745881E-211	1.82698929635635	0.407	0.131	3.36400053031832E-206	Outer bulb K	TAGLN
194	PALLD	2.56240790355105E-207	1.14116936903541	0.632	0.33	9.36944449933441E-203	Outer bulb K	PALLD
195	MARCKSL1	1.58814591516212E-204	1.12971360306706	0.656	0.367	5.80705553879029E-200	Outer bulb K	MARCKSL1
196	KRT151	1.60346368003494E-201	1.10071602696775	0.896	0.606	5.86306494604776E-197	Outer bulb K	KRT15
197	VCAN	8.15108200583403E-194	1.2393982627119	0.368	0.105	2.98044313543321E-189	Outer bulb K	VCAN
198	CHCHD7	2.5960810806177E-172	1.04646974031325	0.518	0.24	9.49257047127863E-168	Outer bulb K	CHCHD7
199	CD24	1.62275001256203E-140	1.0884414865186	0.434	0.194	5.93358542093307E-136	Outer bulb K	CD24
200	ACTA2	8.25571576139511E-134	1.94214731641956	0.254	0.075	3.01870246815412E-129	Outer bulb K	ACTA2
201	S100A7	8.5081766271924E-174	2.92540397911324	0.483	0.097	3.1110147837329E-169	Upper hair folli	S100A7
202	KRT6A	7.37113469900861E-142	2.78399471597031	0.501	0.13	2.6952554026925E-137	Upper hair folli	KRT6A
203	C5orf46	3.72996757994454E-128	1.40433538869858	0.25	0.032	1.36386264560672E-123	Upper hair folli	C5orf46
204	CALML5	3.16122886387548E-97	2.79564981195437	0.584	0.234	1.15590333407607E-92	Upper hair folli	CALML5
205	KRT6B	7.10107896558089E-94	2.3558849418022	0.363	0.092	2.59650952376465E-89	Upper hair folli	KRT6B
206	SBSN1	4.58971290072823E-90	2.34475129419446	0.589	0.222	1.67822852215128E-85	Upper hair folli	SBSN
207	S100A91	4.6131319172531E-90	2.32873571869997	0.51	0.181	1.68679168554359E-85	Upper hair folli	S100A9
208	S100A8	2.60014704794175E-88	2.59845653215363	0.497	0.182	9.50743768079901E-84	Upper hair folli	S100A8
209	TMEM45A1	5.11690100148467E-86	1.57100968832653	0.654	0.316	1.87099485119287E-81	Upper hair folli	TMEM45A
210	ZNF750	2.03435417230525E-81	0.717534099278498	0.379	0.105	7.43861603103416E-77	Upper hair folli	ZNF750
211	CARD181	7.78087016873587E-72	0.920771690699661	0.366	0.112	2.84507517719827E-67	Upper hair folli	CARD18
212	CALML3	7.86398148651655E-59	1.74547670415504	0.637	0.4	2.87546483054477E-54	Upper hair folli	CALML3
213	CSTB1	3.63557636552827E-57	1.35852250473483	0.748	0.681	1.32934849805541E-52	Upper hair folli	CSTB
214	APOC1	2.54762648298196E-52	0.729635914051637	0.416	0.169	9.31539623502355E-48	Upper hair folli	APOC1
215	KRT80	7.33215856552045E-52	0.634182742713157	0.32	0.105	2.68100377948255E-47	Upper hair folli	KRT80
216	CHI3L2	5.78802489934727E-51	0.861477494550503	0.256	0.076	2.11639130444633E-46	Upper hair folli	CHI3L2
217	SPINK5	1.50506158560767E-49	1.94280470030395	0.442	0.211	5.50325768777446E-45	Upper hair folli	SPINK5
218	CREG1	2.47857737493497E-47	1.13772929925101	0.512	0.27	9.06291817144971E-43	Upper hair folli	CREG1
219	METRNL	1.01728812752711E-39	0.642429896224931	0.293	0.106	3.71971403830287E-35	Upper hair folli	METRNL
220	RBP1	3.22929096008129E-36	0.818985134287087	0.567	0.346	1.18079023955372E-31	Upper hair folli	RBP1
221	CHCHD101	1.3955565955405E-35	0.74550526360031	0.481	0.269	5.10285269159386E-31	Upper hair folli	CHCHD10
222	FABP51	2.25114019315325E-34	1.5122411146107	0.444	0.246	8.23129411626484E-30	Upper hair folli	FABP5
223	APOE1	2.42516874241779E-33	0.89631670534732	0.773	0.771	8.86762950665063E-29	Upper hair folli	APOE
224	TMEM401	1.52288307005608E-32	0.79590775023217	0.311	0.127	5.56842194566005E-28	Upper hair folli	TMEM40
225	CSTA1	6.31277189494127E-30	1.02819496457791	0.457	0.244	2.30826504338527E-25	Upper hair folli	CSTA
226	CRABP21	9.11726825407291E-29	1.16962511214418	0.335	0.164	3.33372913710176E-24	Upper hair folli	CRABP2
227	LGALS71	8.25037024511209E-27	1.17379110655294	0.584	0.469	3.01674788012524E-22	Upper hair folli	LGALS7
228	GSTP11	1.33302271059759E-26	0.732302536846962	0.794	0.82	4.87419754130008E-22	Upper hair folli	GSTP1
229	TYMP1	3.31139283585753E-23	0.618312465596153	0.446	0.261	1.2108107904313E-18	Upper hair folli	TYMP
230	NDUFA4	8.72620865531752E-23	0.662770121799869	0.761	0.759	3.19073819481685E-18	Upper hair folli	NDUFA4
231	S100A161	2.39836364220479E-22	0.734989053228519	0.709	0.699	8.76961665772183E-18	Upper hair folli	S100A16
232	ATP5ME1	5.27496559281527E-20	0.72404533419505	0.543	0.449	1.9287911690129E-15	Upper hair folli	ATP5ME
233	COX7B	1.38626053531164E-19	0.610969563896056	0.707	0.67	5.06886164736702E-15	Upper hair folli	COX7B
234	JUP1	1.54930036647971E-19	0.73217371713082	0.65	0.537	5.66501679003307E-15	Upper hair folli	JUP
235	UCHL3	1.15746930871383E-16	1.09267184151364	0.435	0.279	4.23228652731212E-12	Upper hair folli	UCHL3
236	CTSB	3.92890229035586E-16	0.625940547790164	0.438	0.281	1.43660312246862E-11	Upper hair folli	CTSB
237	ATP5F1B2	5.44455721091208E-16	0.692444985107823	0.512	0.406	1.99080234417E-11	Upper hair folli	ATP5F1B
238	PTN2	1.65669585407139E-15	0.661705786032234	0.28	0.161	6.05770839041205E-11	Upper hair folli	PTN

239	ENO1	3.19710556087726E-13	0.600745617827862	0.698	0.686	1.16902164833477E-08	Upper hair folli	ENO1
240	KRTDAP1	5.61636679990442E-13	0.952438582281532	0.418	0.258	2.05362452038505E-08	Upper hair folli	KRTDAP
241	TEX264	8.03078471165827E-12	0.642475960780359	0.341	0.213	2.93645642981785E-07	Upper hair folli	TEX264
242	CTNNBIP11	1.45135235500168E-09	2.04164756527081	0.462	0.332	5.30686988606364E-05	Upper hair folli	CTNNBIP1
243	ATP5MC32	6.24264825399222E-09	0.600072622970562	0.479	0.431	0.000228262433407226	Upper hair folli	ATP5MC3
244	LAP3	1.75309407531437E-08	0.984690341360185	0.33	0.22	0.0006410188486387	Upper hair folli	LAP3
245	H2AFJ	1.48600657643125E-07	0.910572093245889	0.449	0.344	0.00543358304672087	Upper hair folli	H2AFJ
246	KRT171	1.61391080225709E-06	0.679796837532566	0.683	0.602	0.0590126484845305	Upper hair folli	KRT17
247	LYPLA1	2.54894470615323E-06	0.667794281248108	0.361	0.267	0.093202163180493	Upper hair folli	LYPLA1
248	PLD31	2.86064769714051E-06	0.615434317003787	0.383	0.286	0.104599583045943	Upper hair folli	PLD3
249	GJB21	2.76973267616302E-05	0.664828761430709	0.331	0.265	1	Upper hair folli	GJB2
250	BNIP3L	0.00679985793438567	0.710321384667937	0.389	0.33	1	Upper hair folli	BNIP3L
251	CENPF	0	1.82084746717405	0.449	0.028	0	Dividing K	CENPF
252	TOP2A	0	1.71802146355506	0.471	0.017	0	Dividing K	TOP2A
253	NUSAP1	0	1.58274885868456	0.566	0.07	0	Dividing K	NUSAP1
254	UBE2C	0	1.51905186970249	0.412	0.008	0	Dividing K	UBE2C
255	CDK1	0	1.41338282230401	0.456	0.017	0	Dividing K	CDK1
256	TK1	0	1.34570398335693	0.465	0.021	0	Dividing K	TK1
257	MKI67	0	1.29083492368348	0.405	0.009	0	Dividing K	MKI67
258	ASPM	0	1.18708330791772	0.332	0.008	0	Dividing K	ASPM
259	TYMS	0	1.11223586229119	0.443	0.024	0	Dividing K	TYMS
260	BIRC5	0	1.04871673738232	0.372	0.014	0	Dividing K	BIRC5
261	MAD2L1	0	1.03900765026305	0.46	0.038	0	Dividing K	MAD2L1
262	ZWINT	0	0.966862569532931	0.451	0.03	0	Dividing K	ZWINT
263	CDKN3	0	0.962283334003879	0.313	0.012	0	Dividing K	CDKN3
264	PBK	0	0.865114515899389	0.33	0.004	0	Dividing K	PBK
265	CCNB2	0	0.836784267737643	0.295	0.011	0	Dividing K	CCNB2
266	CENPK	0	0.772747974913993	0.427	0.035	0	Dividing K	CENPK
267	PCLAF	7.7184783370319E-301	1.14855119773401	0.337	0.022	2.82226160822081E-296	Dividing K	PCLAF
268	TPX2	7.47499088610514E-294	0.726312913121201	0.308	0.018	2.73323041750434E-289	Dividing K	TPX2
269	CDC20	1.92559518349338E-252	0.830465884290181	0.289	0.019	7.04093878844353E-248	Dividing K	CDC20
270	CENPW	1.99748961923111E-249	1.07558110488663	0.529	0.083	7.30382079271854E-245	Dividing K	CENPW
271	CCNB1	1.86561323786096E-168	1.0202030648768	0.315	0.04	6.8216148042386E-164	Dividing K	CCNB1
272	HMGB2	6.3824295673858E-161	2.21644182031673	0.782	0.347	2.33373537131462E-156	Dividing K	HMGB2
273	STMN11	1.0072976504217E-150	1.78566860915549	0.874	0.449	3.68318385876693E-146	Dividing K	STMN1
274	PTTG11	3.86509346746262E-147	1.71231060129692	0.65	0.21	1.41327142637771E-142	Dividing K	PTTG1
275	SMC4	5.6387874360677E-141	1.00794132746525	0.571	0.16	2.06182262599815E-136	Dividing K	SMC4
276	TUBA1B	3.28655648221672E-134	1.98528911275249	0.905	0.663	1.20172937772254E-129	Dividing K	TUBA1B
277	SMC2	2.96601031320035E-130	0.754824074556526	0.46	0.112	1.08452167102171E-125	Dividing K	SMC2
278	HELLS	8.54720423072615E-126	0.846670511247828	0.469	0.116	3.12528522696502E-121	Dividing K	HELLS
279	HMGB11	3.56339246024359E-121	1.25138896927697	0.98	0.88	1.30295445308807E-116	Dividing K	HMGB1
280	GAPDH	6.61930312713406E-96	0.892910505665604	0.958	0.894	2.42034818843657E-91	Dividing K	GAPDH
281	H2AFZ2	7.29017790143253E-96	1.18204556985606	0.932	0.764	2.66565354965881E-91	Dividing K	H2AFZ
282	DUT	8.59101999188207E-87	1.23185688450747	0.727	0.412	3.14130646003168E-82	Dividing K	DUT
283	PCNA	3.69253351039535E-85	1.12242020591019	0.549	0.231	1.35017487807606E-80	Dividing K	PCNA
284	CKS1B	2.67540896061166E-82	1.03119659354098	0.723	0.387	9.78263286447654E-78	Dividing K	CKS1B
285	RRM1	4.33066471415353E-82	0.750465098123021	0.491	0.179	1.58350755273024E-77	Dividing K	RRM1
286	TUBB1	8.39142336244951E-74	1.10739678923573	0.888	0.697	3.06832395247966E-69	Dividing K	TUBB

287	TMPO	2.220537117003E-70	0.696391193249575	0.484	0.181	8.11939396832146E-66	Dividing K	TMPO
288	DEK1	9.39745508676489E-67	0.940408170093251	0.83	0.567	3.43617945247558E-62	Dividing K	DEK
289	CKS22	3.2598623248537E-66	0.905820048715155	0.672	0.321	1.19196865908276E-61	Dividing K	CKS2
290	H2AFV	3.11975427915518E-59	0.776189909339403	0.668	0.358	1.14073815217309E-54	Dividing K	H2AFV
291	HIST1H4C	7.00107112944344E-59	2.10976976651992	0.679	0.409	2.55994165848099E-54	Dividing K	HIST1H4C
292	RANBP1	1.20980120731434E-58	0.771878353789865	0.771	0.518	4.4236381145449E-54	Dividing K	RANBP1
293	H2AFX	8.06826947136962E-58	0.711324998527626	0.489	0.211	2.9501627322063E-53	Dividing K	H2AFX
294	LDHB1	6.37352185140994E-53	0.753673001815108	0.625	0.35	2.33047826496804E-48	Dividing K	LDHB
295	HIST1H1C	1.45493865570873E-51	0.854940127100673	0.344	0.123	5.31998319459898E-47	Dividing K	HIST1H1C
296	HMGN21	1.43061836107437E-49	1.20183157226071	0.76	0.565	5.23105603726844E-45	Dividing K	HMGN2
297	MT2A2	1.20531861993858E-48	0.848377812629325	0.864	0.638	4.40724753380542E-44	Dividing K	MT2A
298	NUCKS11	2.37804930028368E-44	0.734676736384817	0.866	0.668	8.69533726648729E-40	Dividing K	NUCKS1
299	UBE2S	1.70528310457489E-37	0.712977486134549	0.601	0.355	6.23536767187808E-33	Dividing K	UBE2S
300	LGALS11	8.27164043283491E-10	1.00036999603766	0.595	0.466	3.02452532426609E-05	Dividing K	LGALS1
301	SOSTDC1	0	2.19069299279612	0.752	0.151	0	Inner bulb K	SOSTDC1
302	KRT75	1.35464004990674E-239	1.81154360860622	0.323	0.031	4.953241342484E-235	Inner bulb K	KRT75
303	KRT172	4.23943494091165E-223	2.56778066809131	0.953	0.583	1.55014938614435E-218	Inner bulb K	KRT17
304	NECTIN41	2.86834277095092E-203	0.90125399131126	0.443	0.076	1.0488095341982E-198	Inner bulb K	NECTIN4
305	GJB61	5.6776925804285E-176	1.74671197570852	0.787	0.304	2.07604829203368E-171	Inner bulb K	GJB6
306	KRT51	3.94114032877471E-175	1.44168210384579	0.997	0.874	1.44107796121647E-170	Inner bulb K	KRT5
307	SULF2	4.08409894426511E-171	0.86084387251238	0.416	0.077	1.49335077897054E-166	Inner bulb K	SULF2
308	KRT6B1	7.92427865799423E-153	1.93973283825811	0.412	0.086	2.89751249129559E-148	Inner bulb K	KRT6B
309	HES41	2.05042382025095E-146	1.09070124751901	0.508	0.14	7.49737469874761E-142	Inner bulb K	HES4
310	GJB22	2.34236937564294E-131	1.31780224489281	0.655	0.242	8.56487362203841E-127	Inner bulb K	GJB2
311	RBP11	9.29248521606806E-122	1.32403136293824	0.705	0.335	3.39779721925529E-117	Inner bulb K	RBP1
312	APOE2	3.03664163769715E-121	1.38463143038904	0.953	0.758	1.11034801482396E-116	Inner bulb K	APOE
313	CD242	1.12456888038824E-118	1.32204575952335	0.599	0.219	4.1119861111396E-114	Inner bulb K	CD24
314	GJA11	6.80300697413814E-118	1.28889056297105	0.855	0.514	2.48751950009361E-113	Inner bulb K	GJA1
315	TM4SF1	6.3635333041032E-111	1.31249242394999	0.789	0.447	2.32682595264534E-106	Inner bulb K	TM4SF1
316	CTSV1	9.19040049812548E-107	1.24088643473069	0.445	0.134	3.36046994213958E-102	Inner bulb K	CTSV
317	MTSS11	4.91103841220055E-104	0.960907523548239	0.631	0.259	1.79572119542113E-99	Inner bulb K	MTSS1
318	TMSB101	1.8025855661261E-99	1.0009121260705	0.976	0.928	6.59115412254007E-95	Inner bulb K	TMSB10
319	ID41	6.00094388130368E-97	1.36758707686197	0.636	0.29	2.19424513019869E-92	Inner bulb K	ID4
320	DSP1	8.97433936750272E-95	1.18604795149019	0.953	0.848	3.28146718972737E-90	Inner bulb K	DSP
321	MARCKSL11	3.15487237618396E-93	0.912285054217907	0.768	0.403	1.15357908435166E-88	Inner bulb K	MARCKSL1
322	CD1091	1.54027955476837E-92	0.941700513193925	0.562	0.236	5.63203219201056E-88	Inner bulb K	CD109
323	SOX93	2.19579818184478E-92	0.885392660736361	0.705	0.343	8.02893605191545E-88	Inner bulb K	SOX9
324	PKP12	2.82995328405572E-92	1.04160758319385	0.727	0.387	1.03477241831497E-87	Inner bulb K	PKP1
325	DSC22	1.40286943282091E-91	1.08523123745087	0.615	0.276	5.12959208110966E-87	Inner bulb K	DSC2
326	ACTN11	1.32660291517136E-86	1.05906669235195	0.666	0.349	4.85072355932408E-82	Inner bulb K	ACTN1
327	ALCAM1	3.55484002040686E-86	1.16276974229871	0.464	0.169	1.29982725346177E-81	Inner bulb K	ALCAM
328	TIAM11	1.25972624517145E-82	0.820038488474651	0.584	0.245	4.6061890154694E-78	Inner bulb K	TIAM1
329	CALML31	1.32107569609607E-77	1.24683104475639	0.692	0.394	4.83051328277526E-73	Inner bulb K	CALML3
330	FZD71	2.37714781547281E-75	0.824338964981011	0.544	0.246	8.69204098727632E-71	Inner bulb K	FZD7
331	PALLD1	2.95452470116275E-73	0.891360107068697	0.691	0.372	1.08032195698016E-68	Inner bulb K	PALLD
332	DIO21	4.88263556512462E-73	0.821078997609181	0.432	0.151	1.78533569438782E-68	Inner bulb K	DIO2
333	COPS92	4.05118970645377E-71	0.823603636477555	0.678	0.385	1.48131751616482E-66	Inner bulb K	COPS9
334	FLNB	1.20635740321373E-69	0.83019868605212	0.476	0.205	4.411045844851E-65	Inner bulb K	FLNB

335	SOX42	5.71388333794842E-68	0.873963480650708	0.831	0.544	2.08928144252084E-63	Inner bulb K	SOX4
336	TUBA1A2	5.25164553607442E-67	0.799170436219463	0.762	0.483	1.92026419026561E-62	Inner bulb K	TUBA1A
337	CHCHD71	1.70886901392579E-65	0.832104673532414	0.574	0.278	6.24847954941966E-61	Inner bulb K	CHCHD7
338	SPON21	1.1991572712592E-60	0.964320606671044	0.525	0.233	4.38471856235928E-56	Inner bulb K	SPON2
339	IGFBP71	2.2031521684282E-60	0.823268925755156	0.655	0.332	8.05582590385771E-56	Inner bulb K	IGFBP7
340	APOC12	1.09067120560543E-57	0.849089082370312	0.402	0.167	3.98803926329626E-53	Inner bulb K	APOC1
341	LMO41	1.31763048730282E-56	0.856078640046698	0.773	0.535	4.81791587682277E-52	Inner bulb K	LMO4
342	SDC11	3.59909203464449E-55	0.86200215789997	0.656	0.411	1.31600800246776E-50	Inner bulb K	SDC1
343	MGP	2.00043361974682E-50	1.27134722972679	0.379	0.157	7.31458553060424E-46	Inner bulb K	MGP
344	LGALS72	1.33698393194925E-40	0.973323406742686	0.65	0.463	4.88868174717245E-36	Inner bulb K	LGALS7
345	HES11	1.46554986275283E-33	0.97476500656939	0.683	0.483	5.35878307315573E-29	Inner bulb K	HES1
346	TAGLN1	4.06706651915466E-30	1.04998343512293	0.353	0.176	1.4871228727289E-25	Inner bulb K	TAGLN
347	GADD45G2	5.8149261774603E-27	0.811416773020575	0.421	0.247	2.12622775678836E-22	Inner bulb K	GADD45G
348	IGFBP51	1.16408835686402E-22	0.896417120495974	0.364	0.211	4.25648907687327E-18	Inner bulb K	IGFBP5
349	IER31	2.73371261626419E-15	0.952545321395753	0.771	0.694	9.99582018137002E-11	Inner bulb K	IER3
350	FABP53	1.65659859618664E-09	1.15500883384614	0.342	0.251	6.05735276695644E-05	Inner bulb K	FABP5
351	MGST1	0	2.61128492635944	0.976	0.24	0	Sebocytes	MGST1
352	SAA11	0	2.42655604926543	0.869	0.107	0	Sebocytes	SAA1
353	IL1R2	0	2.05127673628346	0.778	0.126	0	Sebocytes	IL1R2
354	KRT7	0	1.65991302205426	0.742	0.084	0	Sebocytes	KRT7
355	ACSBG1	0	1.28506216782444	0.749	0.068	0	Sebocytes	ACSBG1
356	NNAT	0	1.24146983433505	0.462	0.019	0	Sebocytes	NNAT
357	PLA2G16	0	1.16121814604345	0.691	0.037	0	Sebocytes	PLA2G16
358	PPARG	0	1.13089346650668	0.678	0.029	0	Sebocytes	PPARG
359	TINAGL11	0	1.04888537391603	0.731	0.093	0	Sebocytes	TINAGL1
360	CLMP	0	0.908521392125929	0.589	0.027	0	Sebocytes	CLMP
361	TGFBR3	0	0.774929701327533	0.618	0.069	0	Sebocytes	TGFBR3
362	CIDEA	1.25021440791093E-273	0.879646612009095	0.411	0.035	4.5714089825263E-269	Sebocytes	CIDEA
363	SERPINF1	9.60600445579719E-271	1.15353158903664	0.802	0.155	3.51243552926224E-266	Sebocytes	SERPINF1
364	HPGD	2.5046714344303E-240	0.781314666349457	0.573	0.081	9.15833109999441E-236	Sebocytes	HPGD
365	CHCHD102	8.79010318770988E-229	1.43956326472494	0.891	0.252	3.21410123058612E-224	Sebocytes	CHCHD10
366	LY6E1	1.44885484627397E-228	1.94592452038816	0.936	0.384	5.29773774540077E-224	Sebocytes	LY6E
367	WFDC2	1.16813149933502E-210	1.29128432015818	0.72	0.154	4.27127282731851E-206	Sebocytes	WFDC2
368	TKT1	8.1096029114866E-208	1.62137639094408	0.931	0.37	2.96527630458508E-203	Sebocytes	TKT
369	RPL281	1.60458083286075E-199	1.16083283769942	1	0.976	5.86714981535533E-195	Sebocytes	RPL28
370	RPL12	2.35740911393525E-187	1.14367927105483	0.998	0.966	8.61986642510426E-183	Sebocytes	RPL12
371	SAMM50	9.49772503964544E-182	0.800724471735192	0.758	0.19	3.47284316074636E-177	Sebocytes	SAMM50
372	APMAP	1.10799346321247E-163	1.02856115476103	0.689	0.165	4.0513780982364E-159	Sebocytes	APMAP
373	RPL7A	3.77959438517275E-150	0.876707990413309	0.998	0.958	1.38200868693842E-145	Sebocytes	RPL7A
374	RPL5	1.03583130952772E-146	0.874115151470238	0.998	0.953	3.78751718328811E-142	Sebocytes	RPL5
375	RPL291	1.40876958511662E-135	0.806446794648871	0.996	0.962	5.15116598797893E-131	Sebocytes	RPL29
376	GPX41	2.06071895049085E-122	1.09389564847149	0.971	0.666	7.53501884246978E-118	Sebocytes	GPX4
377	ACADVL1	7.98016675470785E-116	0.785447922671604	0.851	0.325	2.91794797385892E-111	Sebocytes	ACADVL
378	TPT11	1.0621204664726E-114	0.883348174668502	0.998	0.978	3.88364348565706E-110	Sebocytes	TPT1
379	CYB5A3	2.68604244834965E-107	1.2730436931457	0.862	0.372	9.82151421239048E-103	Sebocytes	CYB5A
380	EEF1B21	2.82175301341976E-107	0.821501503016143	0.987	0.875	1.03177398935693E-102	Sebocytes	EEF1B2
381	APOC13	1.30237048555255E-106	1.4815178438823	0.576	0.163	4.76211768042288E-102	Sebocytes	APOC1
382	PLIN21	7.66658700160345E-103	0.964548498763074	0.829	0.317	2.8032875371363E-98	Sebocytes	PLIN2

383	RACK14	5.33653468315227E-102	1.24537314864574	0.82	0.573	1.95130390689463E-97	Sebocytes	RACK1
384	RBM3	5.78268930994286E-98	0.89242633634374	0.971	0.753	2.11444034618061E-93	Sebocytes	RBM3
385	RPS22	7.67545910282217E-98	0.991262172549583	1	0.981	2.80653162094693E-93	Sebocytes	RPS2
386	NSA21	1.26326609212673E-88	0.884257115602837	0.867	0.508	4.61913246586139E-84	Sebocytes	NSA2
387	EEF1A11	2.36484098469191E-88	0.792589597595923	0.991	0.989	8.64704106052595E-84	Sebocytes	EEF1A1
388	RPL372	6.06944316962677E-81	0.787211084325433	0.987	0.976	2.21929189497403E-76	Sebocytes	RPL37
389	PMVK	5.30121669438681E-77	0.828858822645662	0.809	0.342	1.93838988430254E-72	Sebocytes	PMVK
390	MT1X1	5.8764451847765E-77	1.08780610172054	0.929	0.696	2.14872218181353E-72	Sebocytes	MT1X
391	ECH1	1.97083561886085E-76	0.794926960976325	0.86	0.381	7.20636044036469E-72	Sebocytes	ECH1
392	RPS211	3.82729664436382E-76	0.777523908814776	0.982	0.944	1.39945101801163E-71	Sebocytes	RPS21
393	SLC25A61	9.29197349011034E-76	1.00313818640423	0.984	0.772	3.39761010665885E-71	Sebocytes	SLC25A6
394	COMT	1.17164633477118E-75	0.814542373850254	0.853	0.419	4.28412482309083E-71	Sebocytes	COMT
395	NOP532	8.67141007675893E-74	0.839691121089069	0.793	0.426	3.1707010945669E-69	Sebocytes	NOP53
396	MT-ND11	1.71815264126358E-66	0.794149616653162	0.967	0.931	6.28242513278028E-62	Sebocytes	MT-ND1
397	ATP5MC35	4.3005343382012E-63	0.797920870180705	0.793	0.417	1.57249038076327E-58	Sebocytes	ATP5MC3
398	RPS26	1.20030459860317E-58	0.824086333398792	0.96	0.875	4.38891376479249E-54	Sebocytes	RPS26
399	APOE3	1.99170234992758E-41	1.11033353713514	0.98	0.761	7.2826596425102E-37	Sebocytes	APOE
400	FDPS2	1.68862090296195E-33	0.922806509515722	0.631	0.292	6.17444233168039E-29	Sebocytes	FDPS
401	SLURP1	1.29781821588569E-307	5.27221270631946	0.71	0.02	4.74547230638601E-303	Granular K	SLURP1
402	CLIC3	4.33719076185809E-245	3.48220751856837	0.913	0.046	1.58589380207341E-240	Granular K	CLIC3
403	IVL	3.80929039908079E-240	2.40652163513874	0.768	0.031	1.39286703442389E-235	Granular K	IVL
404	C1orf68	2.18624735003331E-231	2.41095777563337	0.493	0.012	7.9940134353968E-227	Granular K	C1orf68
405	SERPINA12	1.63111301231236E-229	2.93398897298241	0.594	0.019	5.96416472952016E-225	Granular K	SERPINA12
406	LYNX1	8.1233548759513E-211	3.33182588205018	0.623	0.023	2.9703047103916E-206	Granular K	LYNX1
407	KRT27	3.71982932276807E-147	2.79843166519159	0.29	0.006	1.36015559187015E-142	Granular K	KRT27
408	KLK7	1.05575375057656E-143	1.72600755550161	0.652	0.038	3.8603635889832E-139	Granular K	KLK7
409	KRT71	1.27015137516719E-138	3.36209188805997	0.29	0.007	4.64430850329884E-134	Granular K	KRT71
410	SULT2B11	1.29433577505507E-128	2.55212398121793	0.899	0.088	4.73273876148887E-124	Granular K	SULT2B1
411	LY6G6C	7.79342699988995E-121	2.47185941050284	0.797	0.069	2.84966658250976E-116	Granular K	LY6G6C
412	SPRR2D	6.95037515030553E-100	1.82268073945548	0.275	0.009	2.54140467370922E-95	Granular K	SPRR2D
413	CNFN	7.78028161251655E-98	3.15333161056454	0.71	0.07	2.84485997161668E-93	Granular K	CNFN
414	KRT21	4.0626150675001E-87	5.20870260204517	0.696	0.077	1.48549519943141E-82	Granular K	KRT2
415	SCEL1	1.5242005475027E-86	2.85042383350122	0.87	0.128	5.57323930194361E-82	Granular K	SCEL
416	KRT25	7.02207466648775E-69	4.78747328166215	0.333	0.021	2.56762160180125E-64	Granular K	KRT25
417	KRT28	8.82900306903671E-68	3.17979955921891	0.275	0.014	3.22832497219327E-63	Granular K	KRT28
418	SPRR1A	3.27482525282848E-67	1.65353839153139	0.348	0.023	1.19743985369673E-62	Granular K	SPRR1A
419	SPINK51	1.42907909380037E-66	3.1247184527014	0.957	0.218	5.22542770648106E-62	Granular K	SPINK5
420	SBSN2	3.18038132144398E-63	3.8662377517207	0.971	0.237	1.16290643018599E-58	Granular K	SBSN
421	CALML51	4.06162348066625E-60	4.97600052195469	0.971	0.248	1.48513262570561E-55	Granular K	CALML5
422	SPRR1B	2.17754325825005E-58	2.56164278073095	0.522	0.06	7.9621869237913E-54	Granular K	SPRR1B
423	FABP54	5.84515209736086E-58	3.93490175907081	0.928	0.252	2.1372798644E-53	Granular K	FABP5
424	TMEM45A2	4.9442056383408E-55	2.87057889682317	1	0.33	1.80784879165931E-50	Granular K	TMEM45A
425	DSC11	3.00726808489222E-53	1.74183571156948	0.783	0.152	1.09960757524084E-48	Granular K	DSC1
426	CLDN42	1.26792961353207E-51	2.12245545542106	0.754	0.137	4.63618463188E-47	Granular K	CLDN4
427	KRTDAP2	1.79906634358368E-50	3.8372118389413	0.899	0.262	6.57828608531373E-46	Granular K	KRTDAP
428	GGH1	2.34681083196918E-45	1.93217514436073	0.681	0.136	8.5811138070953E-41	Granular K	GGH
429	CRABP22	7.47139661546314E-43	2.39994183836902	0.739	0.169	2.7319161724441E-38	Granular K	CRABP2
430	SLPI1	2.29777836502045E-41	2.04283047183511	0.609	0.113	8.40182659169728E-37	Granular K	SLPI

431	SPTSSB	7.35738153046489E-40	1.72123753913082	0.464	0.068	2.69022655661449E-35	Granular K	SPTSSB
432	DSG12	1.16633499028753E-38	1.63354506428147	0.884	0.287	4.26470389198634E-34	Granular K	DSG1
433	DMKN2	2.37174975020344E-36	3.25887350029894	1	0.519	8.67230296161887E-32	Granular K	DMKN
434	ITM2B1	8.79620275104419E-36	1.94243112767435	1	0.838	3.21633153591931E-31	Granular K	ITM2B
435	KLK111	9.356014865504E-36	1.88097558532781	0.739	0.184	3.42102683557154E-31	Granular K	KLK11
436	SDR16C51	2.48243763417601E-35	2.24998986898331	0.652	0.154	9.07703320936457E-31	Granular K	SDR16C5
437	LYPD31	4.85758292436153E-34	2.26684539876472	1	0.419	1.77617519629279E-29	Granular K	LYPD3
438	MAL21	9.25497147766224E-33	1.64210031294273	0.768	0.213	3.3840803208072E-28	Granular K	MAL2
439	CTNNBIP12	2.71857927415548E-32	1.98395505739901	0.899	0.336	9.94048511594952E-28	Granular K	CTNNBIP1
440	DBI3	5.77102805131471E-32	1.93632803793166	0.986	0.548	2.11017640696323E-27	Granular K	DBI
441	HOPX1	1.98960357060095E-30	3.85264197636628	0.928	0.454	7.27498545590237E-26	Granular K	HOPX
442	TACSTD23	7.34698919018367E-27	2.02154520746505	0.971	0.599	2.68642659739066E-22	Granular K	TACSTD2
443	TMEM99	1.25108562543005E-26	1.66678203705598	0.536	0.13	4.57459458938497E-22	Granular K	TMEM99
444	PERP1	2.52517084324885E-24	1.65030215018665	1	0.934	9.23328718833941E-20	Granular K	PERP
445	CALML32	3.33564822065253E-22	1.6463544453432	0.855	0.41	1.2196797718816E-17	Granular K	CALML3
446	KRT101	1.4885087156803E-20	2.90342670730136	1	0.765	5.44273211888501E-16	Granular K	KRT10
447	PHLDA22	6.44534325740238E-20	1.63566724407563	0.841	0.34	2.35673976206918E-15	Granular K	PHLDA2
448	CSTA2	3.64069931403387E-18	2.48202400969197	0.609	0.253	1.33122170417649E-13	Granular K	CSTA
449	LGALS7B2	5.06388694574109E-18	2.02296215497044	0.971	0.602	1.85161026171023E-13	Granular K	LGALS7B
450	CTSC1	0.00369723922866379	2.06903424606501	0.304	0.208	1	Granular K	CTSC
451	SCGB1B2P	0	7.04256727096565	0.481	0.008	0	Secretory lumir	SCGB1B2P
452	PIP	0	6.48314675854116	0.491	0.004	0	Secretory lumir	PIP
453	SCGB1D2	0	6.31862091186273	0.528	0.008	0	Secretory lumir	SCGB1D2
454	AZGP1	0	4.04233407818835	0.546	0.008	0	Secretory lumir	AZGP1
455	AQP5	0	3.52572586228568	0.741	0.002	0	Secretory lumir	AQP5
456	CA6	0	2.39804865624863	0.37	0.002	0	Secretory lumir	CA6
457	C2orf82	0	2.3437537086262	0.296	0.002	0	Secretory lumir	C2orf82
458	LRRC26	0	2.01912580143571	0.481	0.001	0	Secretory lumir	LRRC26
459	KRT19	2.75424497551738E-280	4.63249377636059	0.861	0.055	1.00708967529793E-275	Secretory lumir	KRT19
460	ROPN1B	1.14908883059789E-271	1.88342939869581	0.315	0.005	4.20164330908117E-267	Secretory lumir	ROPN1B
461	KRT18	2.39848007990313E-262	3.64178848896066	0.815	0.053	8.77004241216577E-258	Secretory lumir	KRT18
462	PPP1R1B	1.78563790718306E-240	2.81003864631583	0.648	0.034	6.52918500761486E-236	Secretory lumir	PPP1R1B
463	PRR4	4.14027203391634E-233	2.3429807990239	0.306	0.006	1.51389046920151E-228	Secretory lumir	PRR4
464	MUCL1	1.19355465115101E-224	7.89473506173733	0.481	0.019	4.36423258193369E-220	Secretory lumir	MUCL1
465	SCGB2A2	3.64710690876983E-217	8.06716394707759	0.5	0.021	1.33356464119169E-212	Secretory lumir	SCGB2A2
466	CLDN10	1.96760975019957E-209	2.06757321747424	0.259	0.005	7.19456505160473E-205	Secretory lumir	CLDN10
467	PLA2G2A	1.22097063842715E-149	2.34065108752988	0.352	0.015	4.46447913940886E-145	Secretory lumir	PLA2G2A
468	KRT72	2.34675239017194E-143	3.05398211563546	0.824	0.106	8.58090011466371E-139	Secretory lumir	KRT7
469	DCD	2.05131445540196E-133	10.6406621163572	0.528	0.042	7.50063130617726E-129	Secretory lumir	DCD
470	NCALD	7.32991943211406E-128	2.011406316058	0.315	0.014	2.6801850403525E-123	Secretory lumir	NCALD
471	S100A1	1.70949679055336E-116	2.87617979198539	0.435	0.032	6.25077501465836E-112	Secretory lumir	S100A1
472	COX7A1	3.47945621678884E-108	2.56361141438901	0.361	0.023	1.27226316566884E-103	Secretory lumir	COX7A1
473	TPD52L12	5.52792264582079E-69	2.49084033618506	0.685	0.149	2.02128491544437E-64	Secretory lumir	TPD52L1
474	DBI4	4.11778578867049E-53	2.84692394770382	0.954	0.547	1.50566837362736E-48	Secretory lumir	DBI
475	KRT81	1.27049757892292E-51	2.31399329608061	0.667	0.169	4.64557439733165E-47	Secretory lumir	KRT8
476	ATP5G32	1.30275146457741E-44	2.59598370343085	0.704	0.22	4.76351073022731E-40	Secretory lumir	ATP5G3
477	ATP5J4	9.1944760268553E-33	2.03848446630877	0.593	0.194	3.36196015921964E-28	Secretory lumir	ATP5J
478	PEBP13	1.49880429851323E-32	1.93651818690341	0.944	0.702	5.48037791751363E-28	Secretory lumir	PEBP1

479	ATP5A13	3.39640901849879E-29	2.16669738705431	0.611	0.212	1.24189695761408E-24	Secretory lumir	ATP5A1
480	CALM23	4.26006245729716E-29	2.00235270232903	0.954	0.826	1.5576918375107E-24	Secretory lumir	CALM2
481	CHCHD103	6.24875656608386E-29	2.42361237949637	0.667	0.277	2.28485783838856E-24	Secretory lumir	CHCHD10
482	ATP5C12	3.1352416563122E-28	1.99406073469697	0.519	0.165	1.14640111163056E-23	Secretory lumir	ATP5C1
483	NDRG22	3.92521928084599E-28	2.5942133145151	0.546	0.199	1.43525643004134E-23	Secretory lumir	NDRG2
484	ATP5G23	8.32029165527562E-28	1.85790122635522	0.676	0.287	3.04231464375153E-23	Secretory lumir	ATP5G2
485	TCEB23	5.58376133548713E-26	2.49955023787043	0.648	0.258	2.04170233232087E-21	Secretory lumir	TCEB2
486	ATP5B3	2.34858943797755E-25	2.08637250690837	0.602	0.228	8.58761727996491E-21	Secretory lumir	ATP5B
487	ATP5O3	9.49192331853958E-24	1.89315551697294	0.565	0.21	3.470721761424E-19	Secretory lumir	ATP5O
488	PDLIM4	1.47012850987825E-23	1.83309567891775	0.62	0.292	5.3755248963698E-19	Secretory lumir	PDLIM4
489	ATP5E3	7.53584431714528E-22	2.08056546694326	0.63	0.281	2.75548147456417E-17	Secretory lumir	ATP5E
490	ATP5L3	1.73670686243132E-20	2.3561308396688	0.648	0.296	6.35026864248013E-16	Secretory lumir	ATP5L
491	USMG53	5.60100213738896E-19	2.29305762977531	0.481	0.189	2.04800643153627E-14	Secretory lumir	USMG5
492	ETFB1	8.77104474803422E-15	1.97726293020263	0.62	0.398	3.20713251211871E-10	Secretory lumir	ETFB
493	PDCD41	3.20269915790197E-13	1.89732133809318	0.648	0.436	1.17106694708685E-08	Secretory lumir	PDCD4
494	ALDOA2	5.59224102174212E-13	1.93391498519457	0.648	0.409	2.04480292960001E-08	Secretory lumir	ALDOA
495	UQCR11.11	6.29444798040949E-11	2.11941739993314	0.25	0.089	2.30156490403673E-06	Secretory lumir	UQCR11.1
496	XBP12	1.11646404964869E-10	1.84593284891319	0.519	0.356	4.08235079754042E-06	Secretory lumir	XBP1
497	ZG16B	1.21441049487154E-10	2.15413043534061	0.296	0.12	4.44049197449779E-06	Secretory lumir	ZG16B
498	ATP5I3	1.90214170211019E-10	1.80624510686599	0.398	0.19	6.95518113376589E-06	Secretory lumir	ATP5I
499	GAPDH1	8.17717280004263E-09	2.93183528740535	0.954	0.896	0.000298998323433559	Secretory lumir	GAPDH
500	UQCRB2	0.000410788600614742	1.86194387377169	0.657	0.676	1	Secretory lumir	UQCRB
